# Assessing the Health and Economic Outcomes of a 9-Valent HPV Vaccination Program in the United Kingdom

**DOI:** 10.36469/001c.34721

**Published:** 2022-06-06

**Authors:** Kwame Owusu-Edusei, Cody Palmer, Olga Ovcinnikova, Giampiero Favato, Vincent Daniels

**Affiliations:** 1 Merck & Co., Inc.; 2 MSD (UK) Limited; 3 Kingston University

**Keywords:** HPV, universal vaccination, cost-effectiveness analysis, 4-valent HPV vaccine, 9-valent HPV vaccine

## Abstract

**Background:** The United Kingdom (UK) switched from using the 4-valent human papillomavirus (HPV) vaccine (Gardasil®) to the 9-valent vaccine (Gardasil 9®) in 2021.

**Objective:** To estimate and compare the health and economic outcomes of 2 HPV vaccination programs in the UK targeting girls and boys aged 12-13 years from the perspective of the UK National Health Service. The 2 vaccination strategies were (1) universal vaccination 4-valent (UV4V), using the 4-valent HPV vaccine (4vHPV), and (2) universal vaccination 9-valent (UV9V), using the 9-valent HPV vaccine (9vHPV).

**Methods:** A deterministic heterosexual compartmental disease transmission model was used to track health and economic outcomes over a 100-year time horizon. Outcomes were discounted at an annual rate of 3.5% and 1.5%. All costs were adjusted to 2020 British pounds (£). Health outcomes were measured in quality-adjusted life-years (QALYs), and the summary results were presented as incremental cost-effectiveness ratios (£/QALY gained) when comparing UV4V with UV9V.

**Results:** Using the same vaccine coverage for both programs, the total cumulative cases of HPV-related health outcomes tracked over the 100-year horizon indicated that the relative number of cases averted (UV9V vs UV4V) ranged from 4% (anal male cancers and deaths) to 56% (cervical intraepithelial neoplasia [CIN1]). Assuming that 9vHPV cost £15.18 more than 4vHPV (a cost differential based on discounted list prices), the estimated incremental cost-effectiveness ratio was £8600/QALY gained when discounted at 3.5%, and £3300/QALY gained when discounted at 1.5%. The estimated incremental cost-effectiveness ratios from the sensitivity analyses remained <£28000/QALY over a wide range of parameter inputs and demonstrated that disease utilities, discount rate, and vaccine efficacy were the 3 most influential parameters.

**Discussion:** Consistent with other published studies, the results from this study found that the 9vHPV vaccine prevented a substantial number of cases when compared with the 4vHPV vaccine and was highly cost-effective.

**Conclusions:** These results demonstrate that replacing universal 4vHPV with 9vHPV can prevent a substantial additional number of HPV-related cases/deaths (in both women and men) and remain cost-effective over a range of 9vHPV price premiums.

## BACKGROUND

Known to infect approximately 8 out of every 10 people at some point in their lives, human papillomavirus (HPV) is one of the most prevalent sexually transmitted infections (STIs) in the world.^1,2^ HPV is transmitted through sexual contact (vaginal, anal, and oral), although there is evidence of vertical transmission from mother to child during pregnancy or delivery.^3–5^ HPV infects the epithelial tissues of the cervix, anus, penis, mouth, and throat.^6–8^ Over 100 types of HPV have been identified. Among the 9 types (6, 11, 16, 18, 31, 33, 45, 52, and 58) against which vaccines are directed, 7 (16, 18, 31, 33, 45, 52, and 58) are considered high-risk HPV types because they are known to increase the risk of developing cancers. Types 16 and 18 are known to account for 70% of high-grade cervical intraepithelial neoplasia (CIN) and invasive cervical cancer. Types 6 and 11 affect the genital area and cause 85% of genital warts cases^9,10^ but do not cause cervical cancer. As a result, they are considered low-risk HPV among the 9 vaccine types. A recently published report indicated that HPV causes virtually all cervical cancers, over 90% of anal cancers, 70% of oropharyngeal cancers (OPC), 75% of vaginal cancers, 70% of vulvar cancers, and 60% of penile cancers.^11^

In the United Kingdom (UK), estimates from 2018 indicated that cervical cancer ranks as the second most common female cancer among women aged 15-44 years (13th among women of all ages), with an annual incidence of 3430 new cases and over 1000 cervical cancer–associated deaths.^12^ In addition, there were 3049 new cases of OPC and 872 OPC-associated deaths, although the majority of OPC cases are likely associated with high tobacco and alcohol consumption.^12^

One effective prevention and control strategy is the use of HPV vaccines designed to prevent HPV infection and HPV-related diseases. As a result, in 2008 the Joint Committee on Vaccination and Immunisation (JCVI) recommended that routine immunization against HPV should be introduced for girls aged 12-13 years in the UK. These recommendations were revised in March 2014 (3-dose to 2-dose schedule) and November 2015 (including men who have sex with men).^13,14^ In July 2018, the JCVI concluded that extending the HPV vaccination program to boys aged 12-13 years is “highly likely to be cost-effective.”^15^ Consequently, for the 2019-2020 school year, boys and girls aged 12-13 years in school year 8 were eligible for HPV vaccination.^16^ In July 2021, Public Health England released a statement announcing the switch from the 4-valent HPV vaccine (Gardasil®, [Merck & Co, Inc; 4vHPV]) to the 9-valent HPV vaccine (Gardasil 9® [Merck & Co, Inc; 9vHPV]) during the 2021-2022 academic year.^17^

Several studies have assessed the cost-effectiveness of HPV vaccination in the UK.^18–22^ However, the most recent that examined the 9-valent vaccine concluded that vaccinating girls is cost-effective but less so for both girls and boys, because boys are already protected due to herd effect conferred by the vaccinated girls. Furthermore, their results confirmed that almost all the health gains would be erased within 15 years if vaccination were halted.^23^ Their results were consistent with studies conducted for other settings.^24–26^

### Study Objectives

Given the recent developments in the vaccine used in the UK HPV vaccination program, it is important to assess the health and economic impact at the national level. Specifically, this study focused on the switch from 4vHPV to 9vHPV. Therefore, the objective of this study is twofold:

1. Estimate the cumulative population-level HPV-related health outcomes for 2 vaccination strategies, ie, universal use of 4vHPV and 9vHPV.
2. Compare the health and economic outcomes of the 2 vaccination strategies (universal use of 4vHPV vs. 9vHPV).

The qualitative and quantitative results from this study can provide important information on the assessment of current and future HPV vaccination programs in the UK. In addition, they can provide relevant information that can be used in the decision-making process regarding current and/or future changes to HPV vaccination programs in the UK.

## METHODS AND DATA

### Analytical Design and Scope

To account for herd effects associated with infectious diseases, an age-structured, deterministic, heterosexual, compartmental disease transmission model was used. The disease transmission model was adapted from the most recent model,^27,28^ which is an updated version of the original model developed by Elbasha et al^29^ and Dasbach et al.^18^ In the updated version of the original model, infections and diseases attributable to HPV genotypes 31, 33, 45, 52, and 58 have been added in the form of additional compartments and associated differential equations. In all, the model accounted for the transmission dynamics of all 9 HPV types covered by the 9vHPV vaccine. Additional information is provided in the **Supplementary Online Material, Section 3**.

Due to a lack of data (on HPV genotype coinfections and associated HPV comorbidities) and to keep the model simple, the known HPV-related cancers and precancers (cervical, penile, vaginal, vulvar, anal, and oropharyngeal) and HPV genotypes were modeled separately and independently. In addition, separate models were used for recurrent respiratory papillomavirus (RRP), genital warts, and premalignant cervical lesions due to HPV-6 and 11. Each disease model included HPV acquisition, transmission, persistent infection, reinfection, natural immunity, progression to precancerous lesions, and cancer, as well as treatment, regression, screening (where applicable), and vaccination. Full details of the model structure can be found in the **Supplementary Online Material, Section 3**.^30,31^

### Strategies Examined

For the purpose of this study, 2 national vaccination programs were examined: a universal vaccination (girls and boys) 4-valent (4vHPV, protective against HPV types 6, 11, 16, and 18) vaccination program, and a universal 9-valent (9vHPV, protective against HPV types 6, 11, 16, 18, 31, 33, 45, 52, and 58) vaccination program. We assumed the same vaccination coverage for both scenarios, that both scenarios included the impact of the historical vaccination program in the UK from 2008 through 2019, and that the health and economic comparisons between scenarios begun in 2019. Hereafter, the 2 strategies are referred to as follows:

1. Universal vaccination 4-valent (UV4V), using the 4-valent HPV vaccine (4vHPV)
2. Universal vaccination 9-valent (UV9V), using the 9-valent HPV vaccine (9vHPV)

### Target Population, Time Horizon, Discounting, and Study Perspective

Individuals 12-13 years of age were the target of the vaccination programs. As a result, those who turned 12-13 years old each year were vaccinated and followed over a 100-year time horizon starting in 2019. The model assumed a closed UK population, ie, individuals entered and exited the model population as a result of births and deaths only, respectively. Cost and effects were discounted at a rate of 3.5% and 1.5% based on the JCVI recommendation^15^ and determined from the UK National Health Service perspective.

### Data

The input parameter values used in the models were obtained from the published literature. Table 1 shows the overall all-cause mortality rates by gender used in the model. Sexual activity was categorized as low, medium, and high. The distribution and associated mean number of partners for each category, by age group and gender, together with the mixing proportions among members of different age cohorts, are presented in Table 1. The type-specific vaccine efficacies and assumptions for all the HPV-related cancers and/or infections are presented in Table 2. Table 3 shows the vaccine coverage rate used in the model for both genders. Total costs associated with diagnosing and treating diseases caused by HPV infections and their utilities are presented in Table 4. A comprehensive list of the inputs can be found in the **Supplementary Online Material, Section 3.3**. To be consistent and comparable, all costs (obtained from different sources and from different cost-years) were adjusted to 2020 British pounds (£).

**Table 1.** Mortality Rate, Sexual Activity, and Mixing Parameters

	**All-Cause Mortality Rates for the General Public (%)**	
**Age Group (Years)**	**Males**	**Females**
<1	0.00441	0.00349
1-8	0.00014	0.00012
9-11	0.00008	0.00008
12	0.00009	0.00007
13	0.00013	0.00010
14-17	0.00021	0.00012
18	0.00039	0.00018
19	0.00046	0.00017
20-24	0.00046	0.00021
25-26	0.00058	0.00025
27-29	0.00061	0.00031
30-34	0.00079	0.00043
35-39	0.00119	0.00066
40-44	0.00172	0.00103
45-49	0.00248	0.00156
50-54	0.00368	0.00248
55-59	0.00592	0.00396
60-64	0.00961	0.00614
65-69	0.01434	0.00944
70-74	0.02448	0.01605
75-79	0.04074	0.02809
80-84	0.07318	0.05326
>85	0.16238	0.14371
**Mean No. of sexual partners by age group^32^**		
13-14*	0.0001	0.0001
15*-19	1.70	1.40
20-24	2.00	1.60
25-29	1.70	1.30
30-34	1.50	1.20
35-39	1.20	1.00
40-44	1.10	1.50
45-49	1.10	1.00
50-54	1.10	0.90
55-59	1.00	0.70
60-64	0.90	0.60
65-69	0.80	0.50
70-74	0.50	0.30
75+*	0.5	0.30
**Sexual activity categories and percent population sizes (mean number of sexual partners)^32^**		
Low (mean number of sexual partners/year, ≤1)	85.1 (0.79)	90.7 (0.75)
Medium (mean number of sexual partners/year, 2-4)	11.9 (2.54)	7.6 (2.52)
High (mean number of sexual partners/year, ≥5)	3 (9.80)	1.7 (9.66)
**Sexual mixing parameters^31^**		
Between debut and cessation	0.40	
After cessation	0.10	
Among members of different sexual activity groups	0.50	

**Table 2.** Vaccine Efficacy Estimates and Assumptions Used in the Model

**Vaccine Assumptions**	**HPV-16**	**HPV-18**	**HPV-31, 33, 45, 52, and 58**
Cervical cancer			
Male^a^	0.411	0.411	0.411
Female	0.76	0.76	0.76
Protection against cervical HPV infections becoming persistent	0.988	0.988	0.988
Protection against HPV-related CIN	0.97	0.97	0.97
Vaginal and vulvar cancers			
Male^a^	0.411	0.621	0.621
Female	0.76	0.963	0.963
Protection against vaginal/vulvar HPV infections becoming persistent	0.988	0.984	0.984
Protection against HPV-related /VaIN/VIN	1	1	1
Anal cancers			
Male	0.762	1	0.762
Female	0.762	1	0.762
Protection against anal HPV infections becoming persistent			
Male	0.938	0.999	0.938
Female	0.938	0.999	0.938
Protection against HPV-related AIN	0.655	1	0.655
H&N cancers			
Male	0.411	0.621	0.621
Female	0.760	0.963	0.963
Protection against H&N infections becoming persistent			
Male	0.787	0.96	0.96
Female	0.988	0.984	0.984
Protection against HPV-related H&N neoplasia	0	0	0
Penile cancer			
Male	0.411	0.621	0.621
Female^b^	0.760	0.963	0.963
Protection against penile HPV-16/18 infections becoming persistent			
Protection against HPV-related PIN	0.787	0.960	0.960
Vaccine efficacy against HPV-6/11 infection	HPV-6	HPV-11	
Females	0.761	0.761	
Males	0.49	0.57	
Protection against HPV-6/11–related genital warts			
Females	0.989	1	
Males	0.843	0.909	
Protection against HPV-6/11–related CIN1	1	1	

Abbreviations: AIN, anal intraepithelial neoplasia; CIN, cervical intraepithelial neoplasia; H&N, head and neck; HPV, human papillomavirus; PIN, penile intraepithelial neoplasia; VaIN, vaginal intraepithelial neoplasia; VIN, vulval intraepithelial neoplasia.

^a^Preventing male genital infections through male vaccination is assumed to prevent transmission of genital infections to females.

^b^Preventing female genital infections through vaccination is assumed to prevent transmission of genital infections to males.

Giuliano et al^33^ for males; Garland et al^34^ and Palefsky et al^35^ for females.

**Table 3.** Vaccine Coverage Assumption for Boys and Girls

			**Age (years)**				
**Year**	**Vaccine**	**Routine Uptake (%)**	**13**	**14**	**15**	**16**	**17**
2008-2009	Bivalent	80.9	0	0	0	0	47.4
2009-2010	Bivalent	77.5	0	68.5	68.6	41.7	38.9
2010-2011	Bivalent	83.8	4.5	0.3	7.2	2.2	6.4
2011-2012	Quadrivalent	87.0	0	0	0	0	0
2012-2013	Quadrivalent	85.8	0	0	0	0	0
2013-2014	Quadrivalent	88.1	0	0	0	0	0
2014-2015	Quadrivalent	87.5	0	0	0	0	0
2015-2016	Quadrivalent	85.1	0	0	0	0	0

**Table 4.** Costs and Utility Values Used in the Model

	**Costs (£)**	
	**Male**	**Female**
Disease		
CIN1/2/3 and CIS^21,36,37^		406
Cervical cancer, local disease^38^		20 701
Cervical cancer, regional disease^38^		25 594
Cervical cancer, distant disease^38^		27 239
VaIN1/2/3 and CIS^21,36^		406
Vaginal cancer, all stages^21^		15 893
Vulval cancer, all stages^21^		15 893
Penile cancer, all stages^39^	10 482	
Anal cancer, all stages^40^	18 292	
H&N cancer,^a^ all stages^21^	17 465	
Genital warts^41^	292	
Recurrent respiratory papillomatosis^42^	18 981	
Age-specific healthy state utilities		
1-17	0.93	0.93
18-34	0.92	0.91
35-44	0.90	0.89
45-54	0.87	0.86
55-64	0.81	0.80
65-74	0.76	0.78
75+	0.69	0.70
HPV-related disease utilities		
CIN1, VaIN1, VIN1		0.91
CIN2+, CIS, VaIN2+, VIN2+		0.87
Cervical/vaginal/vulvar/anal/H&N^a^/penile cancer, local	0.76	
Cervical/vaginal/vulvar/anal/H&N^a^/penile cancer, regional	0.67	
Cervical/vaginal/vulvar/anal/H&N^a^/penile cancer, distant	0.48	
Cervical/vaginal/vulvar/anal/H&N^a^/penile cancer, survivor	0.76	
Genital warts	0.91	
RRP	0.79	

Abbreviations: CIN, cervical intraepithelial neoplasia; CIS, carcinoma in situ; H&N, head and neck; HPV, human papillomavirus; RRP, recurrent respiratory papillomatosis; VaIN, vaginal intraepithelial neoplasia.

^a^Comprises cancers of the oral cavity, oropharynx, and larynx.

### Model Verification and Validation

Model validity was assessed and confirmed by comparing its structure with previously validated and published models of HPV infections.^18,29^ In addition, a number of tests were built into the model to ensure internal validity and verify the results.^43^ In particular, tests were used to check for all 3 major types of mathematical disease modeling errors: logic, mechanical, and omission.^43^ For example, to verify the model logic, the total number of persons in each compartment for each age group was ensured to be equal to the size of the population for that age group, after accounting for deaths. Given the large number of inputs, mechanical tests were also included to ensure that the correct inputs (eg, from the right tables, rows, and/or columns) were used in all calculations. Omission tests were used to ensure that the theoretical model structure and its components/compartments were fully represented in the model equations and calculations.^43^

### Model Calibration

Model calibration was performed using the maximum likelihood estimation approach with the assumption that the observed incidence and/or prevalence have reached equilibrium, and the errors were normally distributed around the true burden (incidence/prevalence). The calibration targets were type-specific HPV prevalence, and incidence of HPV-related precancers, cancers, and genital warts. Details on the calibration process and results can be found in the **Supplementary Online Material, Section 10**.

### Software Used

The models were expressed as systems of ordinary differential equations representing the contents and continuous movements from and to applicable compartments to simulate the natural history of HPV infection and disease progression over the time horizon (100 years). The “NDSolve” function in Mathematica® 12 (Wolfram Research, Champaign, Illinois) was used to derive the numerical solutions.

### Outcome Measures of Interest

Based on the specified objectives of this study, the outcomes of interest were as follows:

1. The cumulative health outcomes (ie, diseases and deaths) resulting from each of the 2 strategies listed above.
2. Discounted health outcomes (in quality-adjusted life-years [QALYs]) and costs compared between the strategies and presented as incremental cost-effectiveness ratios (ICERs), reported as pounds per QALYs gained.

### Sensitivity Analyses

One-way sensitivity analyses using the extreme values (upper and lower bounds) were conducted on select inputs: vaccine efficacy, duration of protection, disease and healthy utilities, discount rate, and costs. However, due to the substantial number of inputs for some of the categories (utilities and costs), a modified one-way (one-category) sensitivity analysis was adopted in which the changes in the values were applied to all the inputs in that category. In particular, keeping all other inputs at their base case values, inputs for costs of disease, disease utility, healthy utility categories were varied (ie, all inputs for each category, one category at a time) from low (-20%) to high (+20%); all vaccine efficacies were varied from low (25% reduction) to full (100%); discount rate was varied from 1.5% to 5%; duration of protection was reduced to 30 years; and vaccine coverage rate was varied from low (75%) to high (95%).

Finally, a comprehensive threshold analysis was conducted on the relative price of 9vHPV vs 4vHPV using the estimated UV9V-UV4V ICERs. Then, using the estimated price premiums from the one-way (one-category) sensitivity analysis, tornado diagrams were used to depict the changes in the estimated base case price premium for the specified ranges by setting the willingness-to-pay (WTP) thresholds to £20 000 and then repeated for £30 000.^44^

## RESULTS

### Base-Case Health Outcomes

Table 5 shows the base case results for the health outcomes. Overall, the number of cases estimated for the 2 strategies showed that a substantial number of cases (ranging from 4% to 56%) were prevented when comparing the UV9V with the UV4V strategy based on the inputs and assumptions used in the model. The estimated number of cases for the 2 interventions over a 100-year time horizon indicated 4.1 million and 2.0 million cases of intraepithelial neoplasia (CIN1, CIN2/3, VaIN1, VaIN2/3) for the UV4V and UV9V strategies, respectively (a 53% reduction). The numbers of estimated cancer cases were 314 709 (female, 220 173; male, 94 537) for UV4V and 274 301 (female, 185 661; male, 88 640) for UV9V, representing a 13% reduction in the number of cases (Table 5). However, the percent reduction was higher for females than for males (16% vs 6%).

**Table 5.** Estimated Cumulative HPV-Related Health Outcomes for the UV4V and UV9V Vaccination Strategies Over a 100-Year Time Horizon^a^

**Clinical Outcomes**	**UV4V**	**UV9V**	**Cases Averted, No.^b^ (%)**
CIN1	2 386 845	1 057 643	1 329 202 (56)
CIN2/3	1 615 718	808 398	807 320 (50)
VaIN1	46 928	33 590	13 338 (28)
VaIN2/3	67 449	50 556	16 894 (25)
Total cancers	314 709	274 301	40 409 (13)
Female cancers	220 173	185 661	34 512 (16)
Cervical	106 963	82 500	24 463 (16)
Other	113 209	103 161	10 048 (9)
Anal	34 819	32 932	1887 (5)
Vaginal	14 311	12 873	1437 (10)
Vulvar	54 000	48 069	5931 (11)
H&N^c^	10 080	9286	794 (8)
Male cancers	94 537	88 640	5897 (6)
Anal	27 280	26 104	1176 (4)
H&N^c^	22 932	21 212	1720 (7)
Penile	44 325	41 323	3001 (7)
Total deaths	59 149	53 440	5708 (9)
Female deaths	38 346	33 862	4483 (11)
Cervical	22 821	19 568	3253 (14)
Vaginal	3433	3146	287 (8)
Vulvar	6742	6120	621 (9)
Anal	2505	2376	129 (5)
H&N^c^	2845	2652	193 (7)
Male deaths	20 803	19 578	1225 (6)
Penile	11 133	10 478	654 (6)
Anal	2538	2438	100 (4)
H&N^c^	7132	6662	470 (7)

Abbreviations: CIN, cervical intraepithelial neoplasia; H&N, head and neck; HPV, human papillomavirus; RRP, recurrent respiratory papillomatosis; UV4V, universal vaccination 4-valent; UV9V, universal vaccination 9-valent; VaIN, vaginal intraepithelial neoplasia.

^a^Due to rounding, some of the row/column totals may not match the presented numbers.

^b^UV4V cases minus UV9V cases.

^c^Comprises cancers of the oral cavity, oropharynx, and larynx. The details of how this was derived can be found in the **Supplementary Online Material, Section 10.1.**

The UV9V strategy prevented approximately 9% more of the HPV-attributable deaths when compared with the UV4V strategy (53 440 vs 59 149), although most of the deaths in both strategies (>63%) occurred in females (Table 5). The model estimated that among females, the leading cause of HPV-attributable deaths was cervical cancer (>57%), followed by vulvar cancer (>17%), in both strategies. On the other hand, among males, the model estimated that penile cancer was the leading cause of HPV-attributable deaths (>53%) followed by head and neck, which caused about a third (>34%) of the HPV-attributable deaths in males.

### Base Case Cost-Effectiveness Analyses

**Figure 1.**
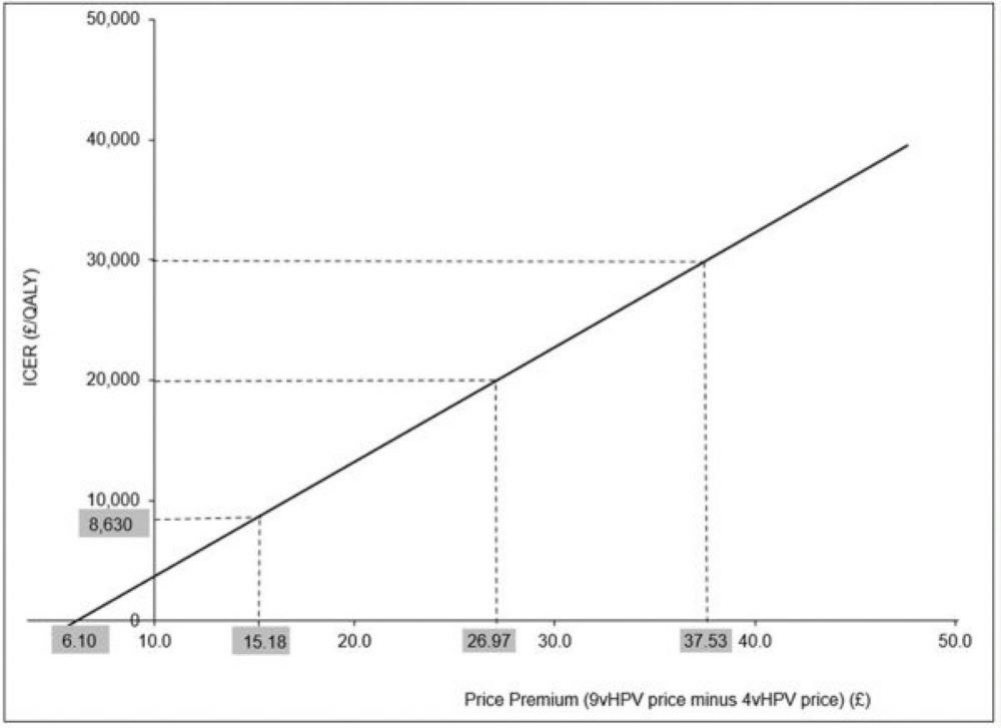
Base Case ICER and Associated Premium Prices With £20 000 and £30 000 Willingness-to-Pay Thresholds Abbreviations: ICER, incremental cost-effectiveness ratio; QALY, quality-adjusted life-year.

Table 6 shows the total discounted QALYs and costs for each strategy examined accumulated over 100 years. When applying a discount rate of 3.5%, the total QALYs estimated were 2 412 029 and 2 412 093 for the UV4V and GNV69 strategies respectively, and the associated costs using a base case price differential of £15.18 (9vHPV over 4vHPV) were estimated at £17.3 million (UV4V) and **£**17.8 million (UV9V). Based on these estimated QALYs and costs, the ICER was **£**8600/QALY gained when comparing the UV9V to the UV4V strategy. However, when applying a discount rate of 1.5%, the total QALYs estimated were 4 459 040 and 4 459 242 for the UV4V and GNV69 strategies, respectively, and the associated costs were estimated at £30.5 (GVNG4) million and £31.2 million (UV9V), respectively. Based on these estimated QALYs and costs, the ICER was £3300/QALY gained when comparing the UV9V with the UV4V strategy. Figure 1 depicts the price-ICER chart for the base case estimates when a 3.5% discount rate was applied and shows that the value-based price premiums were ≈£27 and ≈£37.5 when the WTP thresholds were set to £20 000 and £30 000, respectively. When a 1.5% discount rate was applied, the value-based price premiums were ≈£45 and ≈£63 using the **£**20 000 and £30 000 WTP thresholds, respectively (not shown in chart). In addition, the cost-neutral price premium was £6.10.

**Table 6.** Summary Costs, Effectiveness, and ICERs^a^

**Vaccination Strategy**	**QALYs**	**Cost (£)^b^**	**Incremental QALY**	**Incremental Cost (£)^b^**	**ICER (£/QALY)^b^**
Discounted at 3.5%					
UV4V	2 412 029	17 285 700			
UV9V	2 412 093	17 834 600	64	548 900	8 600
Discounted at 1.5%					
UV4V	4 459 040	30 514 200			
UV9V	4 459 242	31 184 000	202	669 800	3 300

Abbreviations: ICER, incremental cost-effectiveness ratio; QALYs, quality-adjusted life years; UV4V, universal vaccination 4-valent; UV9V, universal vaccination 9-valent.

^a^All costs are reported in 2020 British pounds (£). All costs and QALYs are for the whole population and accumulated over 100 years.

^b^Rounded to the nearest 100.

## Sensitivity Analyses (ICER)

**Figure 2.**
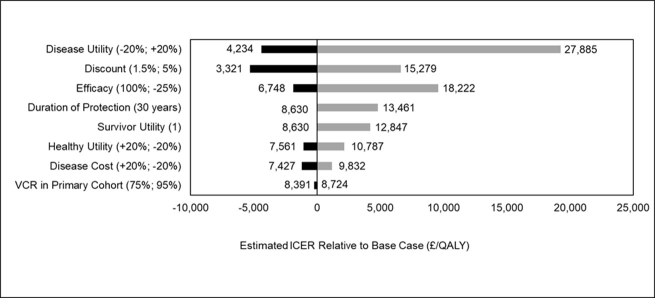
Tornado Diagram Showing Impact of Key Input Parameters on Base Case ICER^a^ Abbreviations: ICER, incremental cost-effectiveness ratio; QALY, quality-adjusted life-year; VCR, vaccine coverage rate. ^a^The labels associated with each bar are the actual absolute estimated ICERs.

Figure 2 shows the estimated ICERs for each of the inputs (or categories of inputs) used in the one-way (one-category) sensitivity analysis. The ICERs ranged from a low of ≈£3300 (when discount rate was 1.5%) to a high of ≈£27 900 (when disease utility was +20%). Based on the estimated ICERs for the low and high inputs, the change in the base case ICER ranged from ≈-£5300 to ≈£19 300. Figure 2 depicts the base case ICER for the select inputs (and the ranges used) in a tornado diagram. As illustrated in Figure 2, the 3 most influential parameters were disease utility, followed by discount rate and vaccine efficacy.

**Figure 3.**
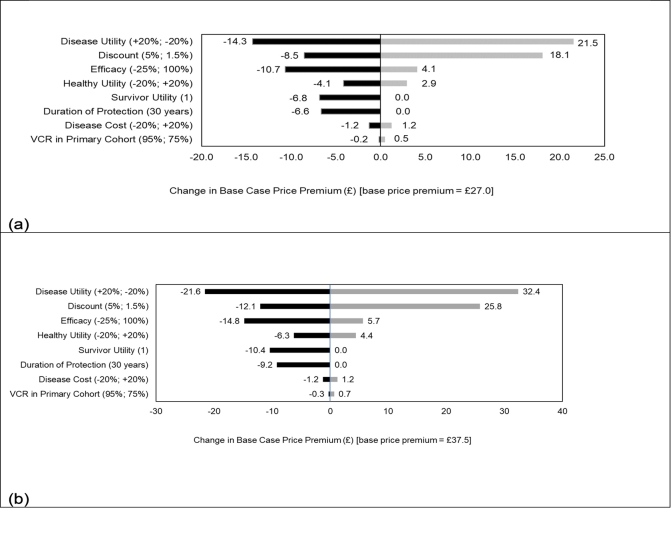
Tornado Diagram Showing Change in Price Premium With Willingness-to-Pay Thresholds of (**a**) £20 000 and (**b**) £30 000 Abbreviation: VCR, vaccine coverage rate.

Using the estimated ICERs from the sensitivity analyses presented in **Figure 2**, the associated changes in the value-based price premium were estimated and presented in tornado diagrams (Figures 3a and 3b). When the WTP is £20 000, the price premium ranged from £12.7 (ie, £27.0 – £14.3) to £48.5 (ie, £27 + £21.5). For a WTP threshold of £30 000, the price premium ranged from £15.9 (ie, £37.5 – £21.6) to £69.9 (ie, £37.5 + £32.4).

## DISCUSSION

In this study, an age-structured, deterministic, heterosexual compartmental disease transmission model was used to determine and compare the health and economic outcomes of 2 HPV vaccination programs targeting individuals aged 12-13 years in the UK: universal vaccination 4-valent (UV4V), using the 4vHPV vaccine, and universal vaccination 9-valent (UV9V), using the 9vHPV vaccine. The model predicted a substantial reduction (up to 56%) in the overall total number of clinical outcomes (cancers, precancers, genital warts, and RRP cases) over a 100-year time horizon when comparing the UV9V with the UV4V program. There were substantial reductions in the burden of HPV-related diseases and deaths (attributable to HPV) among males and females, although the relative reduction in females was substantially higher. Based on the health and economic outcomes, the estimated base case ICER was highly favorable for the UV9V program as the ICERs are low (<£9000/QALY gained). These results were robust when the uncertainties around influential input parameters were incorporated: all the estimated ICERs from the sensitivity analyses were <£28 000. In addition, this study determined that the price premium of 9vHPV (over 4vHPV) can be as high as £70 per dose and remain cost-effective.

The results reported in this study are generally consistent with those of previously published studies on the relative health and economic outcomes of the 9-valent vaccine when compared with the 4-valent vaccines in different settings.^24–26^ Datta et al^23^ used an individual-based model to assess girls only and universal vaccination strategies using 2vHPV (bivalent HPV vaccine), 4vHPV, and 9vHPV vaccines in the UK setting. Although these investigators^23^ included and examined threshold prices for both 4vHPV and 9vHPV vaccines, their results were reported relative to “halted” and “girls-only” vaccination programs. As a result, the direct comparison of 9vHPV with 4vHPV price was not assessed and reported. Thus, comparing the price premium estimates obtained from this study to that of Datta et al is not possible.

### Limitations

There are several notable limitations of this study. First, being a mathematical model, it has all the applicable inherent limitations of models in general, which are simplifications of real-world events. Because of the complex nature of HPV infections and lack of data, several simplifying assumptions were made. For example, dependencies between and among HPV genotype infections and/or diseases were ignored as the 7 disease areas and 9 types were modeled individually (and independent of) each other. Consequently, the potential impact of coinfection and comorbidity was not captured. However, because the impact of coinfection and comorbidity is largely dependent on the type of interaction between and among the infections/diseases (either synergistic or competitive) and there are no data on these phenomena, it is difficult to assess how these omissions impacted the final results. Additional model limitations are discussed elsewhere.^29–31^ Besides the lack of data, there were substantial uncertainties around the available data. However, the comprehensive sensitivity analyses conducted and presented on key data inputs addressed some of the uncertainties around the inputs and their qualitative and quantitative impact on the results.

This study focused on heterosexual transmission. Thus, specific information on the impact of vaccination on same-sex transmission and burden was ignored. Including same-sex transmission dynamics would have introduced additional dimensions of complexity. Nonetheless, given that the model was calibrated to the overall HPV burden (which included the transmission and burden of HPV for the same-sex subpopulations), the population-level impact from same-sex transmission was captured in the overall results. It is expected that because of the higher burden in certain subpopulations, the impact of vaccination would be substantially higher than in the general population. Given the unique transmission dynamics and burden of STIs among the same-sex population (particularly among men who have sex with men), more analysis focusing on this population is recommended. For most STI prevention and control efforts, there is the potential for perverse effect from the intervention on sexual risk behavior (ie, increased sexual activity and/or reduced preventive measures) as a result of perceived risk reduction. However, recently published studies on vaccination and sexual behavior from different settings suggest that vaccination does not affect sexual behavior.^45–47^ In addition, it is difficult to predict the time, direction, and magnitude of the changes in sexual behavior, if any. For these reasons, sexual behavior was assumed to be constant over time.

### Strengths

Despite the above limitations, there are numerous noteworthy strengths in this study. First, dynamic transmission models that account for herd effects are preferred to cohort or static models when assessing the impact of interventions for infectious diseases, particularly vaccination programs.^48–51^ Second, this model was adapted from earlier validated and published modeling efforts in the HPV area.^18,27,29^ The model accounted for all 9 vaccine HPV types (6, 11, 16, 18, 31, 33, 45, 52, and 58) and their associated health outcomes (cervical, penile, vaginal, vulvar, anal, head and neck, RRP, genital warts, premalignant cervical lesions, and deaths).

Because of the complexity of the model, with its increased potential for errors, several tests and checks were built in to ensure internal and external validity and verify the model components and associated calculations. For the purpose of complete transparency and replicability of this study and its results, all the associated study information (ie, model equations, assumptions, parameter inputs, and their sources) have been made publicly available. Finally, to our knowledge, this is the first study that has directly compared the health and economic outcomes of using 9vHPV with that of 4vHPV in the UK setting.

## CONCLUSION

An analysis of the health and economic outcomes of 2 universal vaccination strategies, comparing the 9vHPV with the 4vHPV vaccine, targeting young girls and boys in the UK, showed that using 9vHPV can prevent as much as 56% of the number of cases predicted under the 4vHPV program and was highly cost-effective. In addition, the results remained favorable under a wide range of uncertainties around the major drivers (influential parameter inputs) of the results. These results suggest that switching from using 4vHPV to 9vHPV vaccine in the national vaccination programs can have substantial health and economic benefits. Given the high incremental health benefits and associated treatment cost savings, including the 9vHPV vaccine in the decision-making process for current and future HPV vaccination programs can result in substantial individual- and population-level benefits.

### Disclosures

This study was performed by Merck Sharp & Dohme Corp., a subsidiary of Merck & Co, Inc, Kenilworth, New Jersey. KOE, CP, OO, and VD are employees of Merck Sharp & Dohme Corp, a subsidiary of Merck & Co, Inc, Kenilworth, New Jersey, and may hold stock or stock options in Merck & Co, Inc, Kenilworth, New Jersey.

### Author Contributions

KOE, CP, VD, OO, and GF conceptualized and determined the scope of the study. CP and VD performed the model calibration and study analyses. KOE prepared the first draft. KOE, CP, VD, OO, and GF reviewed the data, results and the interpretation of the results. KOE, CP, VD, OO, and GF critically reviewed and revised the manuscript for intellectual content.

## Supplementary Material

Online Supplementary Material

Online Supplementary Material
