# Supplementary material for: Assessing the Health and Economic Outcomes of a 9-Valent HPV Vaccination Program in the United Kingdom
Source: J Health Econ Outcomes Res. 2022 Jun 6;9(1):140–50. doi: 10.36469/001c.34721 (PMC9170517; doi:10.36469/001c.34721)
Supplement: Online Supplementary Material [file jheor_2022_9_1_34721_91736.pdf]

Journal of Health Economics and Outcomes Research

Online Supplementary Material

Assessing the Health and Economic Outcomes of a 9-Valent HPV Vaccination Program in the United Kingdom.
JHEOR. 2022;9(1):140-150. doi:10.36469/jheor.2022.34721

1. Table of Contents

2. Abbreviations

3. Methods

4. Treatment Patterns

5. Cancer Mortality

6. Vaccine Properties

7. Vaccination Strategy

8. Costs

9. Health-Related Quality of Life

10. Calibration Process

11. Calibration Results

12. References

This supplementary material has been provided by the authors to give readers additional information about their work.

This is an open-access article distributed under the terms of the Creative Commons Attribution 4.0 International License (CCBY-4.0). View this license's legal deed at http://creativecommons.org/licenses/by/4.0 and legal code at http://creativecommons.org/licenses/by/4.0/legalcode for more information.

1 Table of Contents

1.1 Text

Contents

1 Table of Contents ... 1
1.1 Text ... 1
1.2 Tables ... 2
1.3 Figures ... 3
2 Abbreviations ... 4
3 Methods ... 6
3.1 Model description ... 6
3.2 Model outcomes ... 7
3.3 Data sources ... 8
3.3.1 Previous economic evaluations ... 8
3.3.2 Gardasil 9® trial ... 8
3.4 Model parameters ... 8
3.4.1 Demographics ... 8
3.4.2 Sexual behavioral data ... 9
3.5 Screening parameters ... 10
3.5.1 Cervical cancer screening information ... 10
3.6 Natural history of disease ... 12
4 Treatment patterns ... 15
5 Cancer mortality ... 17
6 Vaccine properties ... 20
6.1 Efficacy against infection vs efficacy against disease ... 20
6.2 Efficacy against non-cervical cancers ... 20
6.3 Efficacy for less than 3 doses ... 20
7 Vaccination strategy ... 22
8 Costs ... 23
8.1 Cost of vaccine administration ... 23
8.2 Cost of vaccination ... 23
8.3 Cost per episode of care ... 23
8.4 Cost of screening and diagnostic tests ... 24
9 Health-related quality of life ... 24
10 Calibration process ... 25
10.1 Data sources ... 25
11 Calibration results ... 28
11.1 Anal ... 28
11.2 Cervical ... 35
11.3 Genital warts ... 46
11.4 Head and neck ... 50
11.5 Penile ... 57
11.6 Vaginal ... 60
11.7 Vulvar ... 64
12 References ... 68

1.2 Tables

Table 1: Annual all-cause mortality rates for the general population 9
Table 2: Percent of the population in each of the following sexual activity categories 9
Table 3: Mean number of sexual partners per year by activity category and gender 9
Table 4: Mean number of sexual partners per year by age group and gender 10
Table 5: Sexual mixing 10
Table 6: Cervical cancer screening information Cervical 11
Table 7: Percentage of females screened for cervical cancer in the past year 11
Table 8: Diagnostic performance of screening tests by cervical disease (International) 12
Table 9: Probability of transmitting HPV infection per sexual partnership, by anatomical site, and HPV genotype 12
Table 10: Recurrence rate of treated CIN by stage 12
Table 11: Rate of cancer progression (Same for all diseases) 13
Table 12: Parameters of natural history of cervical disease 13
Table 13:Parameters for Natural History of Vaginal Disease 13
Table 14: Parameters for Natural History of Vulvar Disease 14
Table 15: Natural History Parameters for Anal Disease 14
Table 16: Natural History Parameters for Head and Neck Cancer 15
Table 17: Natural history Parameters for Penile Cancer 15
Table 18: Women receiving hysterectomy over the course of 1 year 16
Table 19: Treatment patterns of all stages of diseases 17
Table 20:Cervical cancer survival by age (2007-2011) 18
Table 21: Five-year relative survival by stage (2002-2006) 18
Table 22: Correspondence between TNM and summary stage 18
Table 23: Annual cancer-associated mortality by site, age and stage 19
Table 24: Vaccine efficacy assumptions (international) for HPV-related cancers 21
Table 25: Vaccine efficacy assumption (international) for genital warts 22
Table 26: Coverage of HPV vaccination programme for girls (from Datta et.al (2019)[78]) 22
Table 27: Percentage of individuals receiving two-or three-dose vaccine 22
Table 28: Costs of diagnosing and treating diseases caused by HPV infection 23
Table 29: Screening and diagnostic tests for cervical and vaginal cancers 24
Table 30: Age-specific utilities in healthy population 24
Table 31: Utilities in population with HPV-related diseases 24
Table 32: Cancer Incidence (cases per 100,000) Data for Females 25
Table 33: Cancer Incidence (cases per 100,000) Data for Males 26
Table 34: HPV Type Attributions for Male Disease 26
Table 35: HPV Type Attribution for Female Disease 27
Table 36: Prevalence of HPV in Britain 27
Table 37: Genital Warts Incidence (cases per 100,000) in the UK 27

1.3 Figures

Figure 1: A simplified schematic diagram of the pre-vaccination model compartments for HPV infection and disease 7
Figure 2. HPV 16 male anal cancer incidence 28
Figure 3. HPV 18 male anal cancer incidence 29
Figure 4. HPV 31 male anal cancer incidence 29
Figure 5. HPV 33 male anal cancer incidence 29
Figure 6. HPV 45 male anal cancer incidence 30
Figure 7. HPV 52 male anal cancer incidence 30
Figure 8. HPV 58 male anal cancer incidence 31
Figure 9. HPV 16 female anal cancer incidence 31
Figure 10. HPV 18 female anal cancer incidence 32
Figure 11. HPV 31 female anal cancer incidence 33
Figure 12. HPV 33 female anal cancer incidence 33
Figure 13. HPV 45 female anal cancer incidence 33
Figure 14. HPV 52 female anal cancer incidence 34
Figure 15. HPV 58 female anal cancer incidence 34
Figure 16. HPV 16 cervical cancer incidence 35
Figure 17. HPV 18 cervical cancer incidence 36
Figure 18. HPV 31 cervical cancer incidence 36
Figure 19 HPV 33 cervical cancer incidence 37
Figure 20. HPV 45 cervical cancer incidence 37
Figure 21. HPV 52 cervical cancer incidence 38
Figure 22. HPV 58 cervical cancer incidence 38
Figure 23. HPV 16 CIN1 incidence 39
Figure 24. HPV 18 CIN1 incidence 39
Figure 25. HPV 31 CIN1 incidence 40
Figure 26. HPV 33 CIN1 incidence 40
Figure 27. HPV 45 CIN1 incidence 41
Figure 28. HPV 52 CIN1 incidence 41
Figure 29. HPV 58 CIN1 incidence 42
Figure 30. HPV 16 CIN2/3 incidence 42
Figure 31. HPV 18 CIN2/3 incidence 42
Figure 32. HPV 31 CIN2/3 incidence 43
Figure 33. HPV 33 CIN2/3 incidence 44
Figure 34. HPV 45 CIN2/3 incidence 44
Figure 35. HPV 52 CIN2/3 incidence 45
Figure 36. HPV 58 CIN2/3 incidence 45
Figure 37. Cervical infection prevalence by HPV type 45
Figure 38. HPV 6 male genital warts incidence 46
Figure 39. HPV 11 male genital warts incidence 47
Figure 40. HPV 6 female genital warts incidence 47
Figure 41. HPV 11 male genital warts incidence 48
Figure 42. HPV 6 CIN1 incidence 48
Figure 43. HPV 11 CIN1 incidence 49

Figure 44. HPV 6 CIN2/3 incidence 49
Figure 45. HPV 11 CIN2/3 incidence 49
Figure 46. HPV 16 male head and neck cancer incidence 50
Figure 47. HPV 18 male head and neck cancer incidence 51
Figure 48. HPV 31 male head and neck cancer incidence 51
Figure 49. HPV 33 male head and neck cancer incidence 51
Figure 50. HPV 45 male head and neck cancer incidence 52
Figure 51. HPV 52 male head and neck cancer incidence 53
Figure 52. HPV 58 male head and neck cancer incidence 53
Figure 53. HPV 16 female head and neck cancer incidence 54
Figure 54. HPV 18 female head and neck cancer incidence 54
Figure 55. HPV 31 female head and neck cancer incidence 55
Figure 56. HPV 33 female head and neck cancer incidence 55
Figure 57. HPV 45 female head and neck cancer incidence 56
Figure 58. HPV 52 female head and neck cancer incidence 56
Figure 59. HPV 58 female head and neck cancer incidence 56
Figure 60. HPV 16 penile cancer incidence 57
Figure 61. HPV 18 penile cancer incidence 57
Figure 62. HPV 31 penile cancer incidence 58
Figure 63. HPV 33 penile cancer incidence 59
Figure 64. HPV 45 penile cancer incidence 59
Figure 65. HPV 52 penile cancer incidence 59
Figure 66. HPV 58 penile cancer incidence 60
Figure 67. HPV 16 vaginal cancer incidence 60
Figure 68. HPV 18 penile cancer incidence 61
Figure 69. HPV 31 vaginal cancer incidence 62
Figure 70. HPV 33 vaginal cancer incidence 62
Figure 71. HPV 45 vaginal cancer incidence 63
Figure 72. HPV 52 vaginal cancer incidence 63
Figure 73. HPV 58 vaginal cancer incidence 63
Figure 74. Vaginal infection prevalence by HPV type 64
Figure 75. HPV 16 vulvar cancer incidence 65
Figure 76. HPV 18 vulvar cancer incidence 65
Figure 77. HPV 31 vulvar cancer incidence 66
Figure 78. HPV 33 vulvar cancer incidence 66
Figure 79. HPV 45 vulvar cancer incidence 67
Figure 80. HPV 52 vulvar cancer incidence 67
Figure 81. HPV 58 vulvar cancer incidence 67
Figure 82. Vulvar infection prevalence by HPV type 68

2 Abbreviations

AC	Anal cancer
ACIP	Advisory Committee on Immunization Practices
CC	Cervical cancer
C-E	Cost-effective
CEA	Cost-effectiveness analysis

CHMP	Committee for Medicinal Products for Human Use
CIN	Cervical intraepithelial neoplasia
CIS	Carcinoma In Situ
EMA	European Medicine Agency
EU	Europe
EUROCARE	European Cancer Registry
EUROGIN	European Research Organisation on Genital Infection and Neoplasia
GUM	Genito-urinary Medicine
GW	Genital warts (condyloma acuminata)
FDA	Food and Drug Administration of the United States
H&N	Head and neck
HEOR	Health Economics and Outcomes Research
HES	Hospital Episodes Statistics
HIV	Human immunodeficiency virus
HPA	Health Protection Agency
HPV	Human papillomavirus
ICER	Incremental cost-effectiveness ratio
IPV	International Papillomavirus Conference
JCVI	Joint Committee on Vaccination and Immunisation
MSM	Men who have sex with men
NATSAL	National Survey of Sexual Attitudes and Lifestyles
NHS	National Health Service
NICE	National Institute for Health and Care Excellence
ONS	Office for National Statistics
OR	Odds ratio
Pap	Papanicolaou smear
PC	Penile cancer
QALY	Quality-adjusted life-year
QoL	Quality of life
RRP	Recurrent respiratory papillomatosis
SPMSD	Sanofi Pasteur MSD
TNM	Tumor-node-metastasis
TTO	Time trade-off
UBC	United BioSource Corporation
US	United States
VaC	Vaginal cancer
VaIN	Vaginal intraepithelial neoplasia
VC	Vulvar cancer
VIN	Vulvar intraepithelial neoplasia

3 Methods

3.1 Model description

A detailed description of the original model including the model structure (relating to the natural history of HPV-related infections), stratifications, input parameters (demographic and epidemiological), model calibration and validation, impact of vaccination and economic model was published by Elbasha et al (2007).[1,2] The original model was updated in 2019 to include all HPV-related diseases (i.e., cervical cancer, vaginal cancer, vulvar cancer, anal cancer, penile cancer, and the associated precancerous lesions, head and neck cancer, genital warts, and recurrent respiratory papillomatosis) and the option to vaccinate boys.[3] It was also extended to investigate the 9-valent HPV vaccine, to evaluate the cost-effectiveness of vaccination to provide protection against HPV types 6, 11, 16, and 18 and 5 new HPV types: 31, 33, 45, 52, and 58.[3]

A simplified model schema is depicted in **Figure 1**. The model was formulated as a system of ordinary differential equations. These equations were programmed and numerically solved using the NDSolve function in Mathematica® 12 (Wolfram Research, Champaign, IL). To extend the original model, compartments and differential equations were added to account for infections and diseases attributable to HPV genotypes 31, 33, 45, 52, and 58. Altogether, the model accounted for the transmission dynamics of all 9 HPV types covered by the 9-valent HPV vaccine.

The model includes several modules that can be summarized as follows:

- Population characteristics: demographics (i.e., population size), and behavior (i.e., sexual activity through sexual partnership)
- Screening strategies: screening coverage rate, cytological test and colposcopy characteristics (for cervical and vaginal cancers only)
- Natural history of the disease/infection: herd immunity or transmission of infection
- Disease and patterns: disease management and/or clinical pathways (from diagnosis to treatment)
- Vaccination assumptions: such as vaccine efficacy, duration of protection, degree of protection, compliance/adherence
- Vaccination strategies: gender type, uptake/coverage rate
- The model allows to assess the impact of a bivalent, quadrivalent and nonavalent HPV vaccines
- Costs: cost information on diagnosis and treatment, defined as an "episode of care" for a specific disease state. An "episode of care" is defined to begin with the diagnosis and end with the resolution of the case. It entails the costs of diagnosing and treating the case. Cost of vaccination including dose and administration
- Health utilities: utility values for the general population and for each disease stage of the different HPV-related diseases

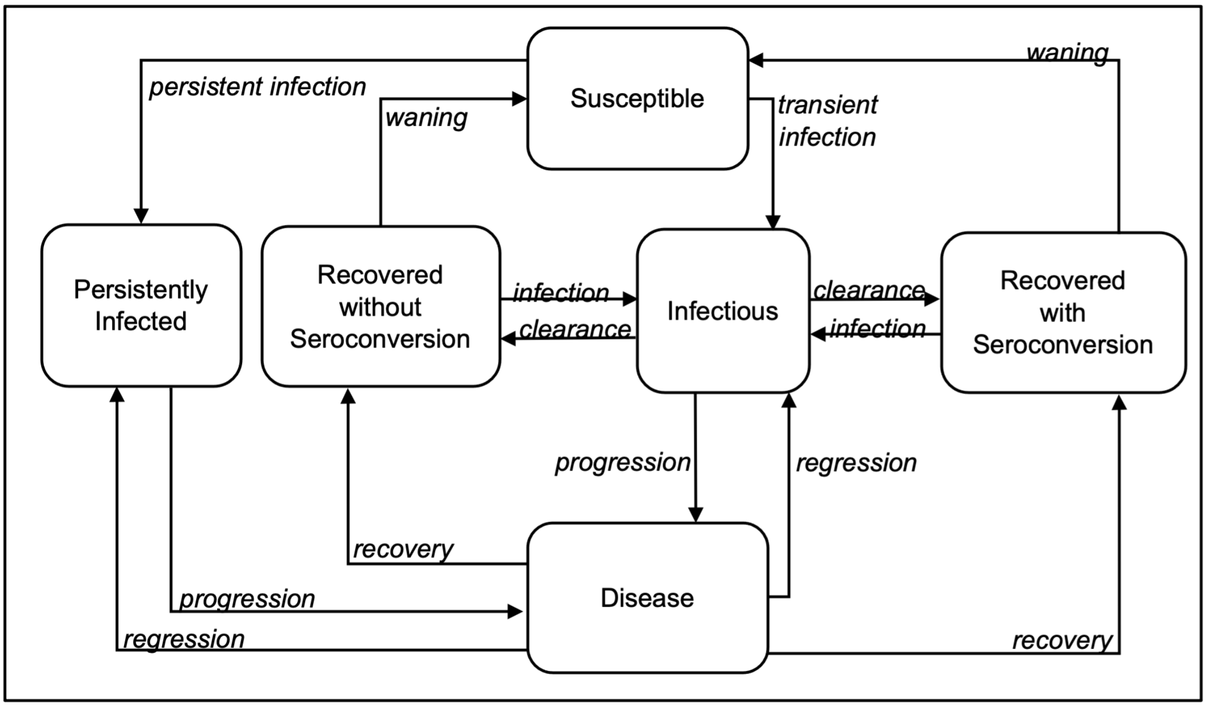

Figure 1: A simplified schematic diagram of the pre-vaccination model compartments for HPV infection and disease

3.2 Model outcomes

Several epidemiological and economic output measures were used to assess the epidemiological impact and cost-effectiveness of the vaccination strategy:

- Diseases included in the model:
  - Cervical cancer
  - CIN 1, 2, and 3
  - Vaginal cancer
  - Vulvar cancer
  - Anal cancer
  - Genital warts
  - Penile cancer (HPV-associated disease not included in the label)
  - H&N cancer (HPV-associated disease not included in the label)
  - RRP (HPV-associated disease not included in the label)
- Epidemiological outputs included:
  - Estimated HPV-16/18/31/33/45/52/58–related HPV infection prevalence among females and males
  - Estimated HPV-6/11–related HPV infection prevalence among females and males
  - Cases of HPV-16/18/31/33/45/52/58–related CIN, cervical cancers and cervical cancer deaths
  - Cases of HPV-16/18–related vaginal, vulvar, penile, anal, and H&N cancers and cancer deaths
  - Cases of HPV-6/11–related genital warts cases, RRP and RRP deaths
- Economic outputs included total costs, survival, quality-adjusted survival, and incremental cost per quality-adjusted life-year (QALY) ratios;

The total costs of each strategy included the cost of vaccination, cost of cytology screening, cost of following false-positive results, and total cost of managing detected precancerous lesions (CIN, VaIN, VIN), cervical cancer, vaginal and vulvar cancers, and genital warts.

The quality-adjusted survival time (i.e., QALYs) was measured by weighting the survival time by the health-utility weights (or quality-of-life adjustment weights) associated with each health state and then integrating the sum of all these adjusted health states over the planning horizon (i.e., 100 years). The incremental cost-effectiveness ratio (ICER) was measured as the incremental cost difference between the 2 strategies divided by the incremental QALY difference between the 2 strategies.

3.3 Data sources

3.3.1 Previous economic evaluations

Previous economic evaluations published on HPV-related diseases in the UK were searched. Input parameters used in these studies were collected and were used to inform the current analysis where appropriate. Publications from Dasbach et al (2008), Choi et al (2010), Jit et al (2008), Jit et al (2011), Jit et al (2015), and Datta et al (2019) were used.[4-9]

3.3.2 Gardasil 9® trial

Efficacy and/or immunogenicity of Gardasil 9® were assessed in 7 clinical studies. Clinical studies evaluating the efficacy of Gardasil 9® against placebo were not acceptable because HPV vaccination is recommended and implemented in many countries for protection against HPV infection and disease. Therefore, the pivotal clinical study (Protocol 001) evaluated the efficacy of Gardasil 9® using the quadrivalent HPV vaccine (Gardasil®) as a comparator. Efficacy against HPV types 6, 11, 16, and 18 was primarily assessed using a bridging strategy that demonstrated comparable immunogenicity (as measured by Geometric Mean Titers [GMT]) of Gardasil 9® compared with Gardasil® (Protocol 001 and GDS01C/Protocol 009).

In the pivotal study Protocol 001, the efficacy of Gardasil 9® against HPV types 31, 33, 45, 52, and 58 was evaluated compared to Gardasil® in women 16 to 26 years of age (N=14,204: 7,099 receiving Gardasil 9®; 7,105 receiving Gardasil®).[10-13]

Protocol 002 evaluated immunogenicity of Gardasil 9® in girls and boys 9 to 15 years of age and women 16 to 26 years of age (N=3,066: 1,932 girls; 666 boys; and 468 women receiving Gardasil 9®). A 36-month study extension (Protocol 002-010) was performed.[14]

Protocol 003 evaluated immunogenicity of Gardasil 9® in men 16 to 26 years of age and women 16 to 26 years of age (1,103 Heterosexual Men [HM]; 313 men who have sex with men [MSM]; and 1,099 women receiving Gardasil 9®).[15]

Protocols 005 and 007 evaluated Gardasil 9® concomitantly administered with vaccines recommended routinely in girls and boys 11 to 15 years of age (N=2,295).[16-18]

Protocol 006 evaluated administration of Gardasil 9® to girls and women 12 to 26 years of age previously vaccinated with Gardasil® (N=921; 615 receiving Gardasil 9® and 306 receiving placebo).[19] GDS01C/Protocol 009 evaluated immunogenicity of Gardasil 9® in girls 9 to 15 years of age (N=600; 300 receiving Gardasil 9® and 300 receiving Gardasil®).[20] Clinical studies for Gardasil 9® are described in the Summary of Product Characteristics.[21]

3.4 Model parameters

3.4.1 Demographics

The population of the UK in mid-2018, 66,435,550, was extracted from the National Records of Scotland, Northern Ireland Statistics, and Research Agency provided by the Office for National Statistics (ONS). The gender and age distribution is shown in **Table 1**.

Annual all-cause mortality rates for the general population entered in the model were obtained from the death rates by age and sex from 2013 in the UK. Where the age groups in the model did not match the age groups given in the data, the death rate was computed as follows:

$$deathrate = \frac{\sum deathrate_i * population_i}{\sum population_i}$$

Table 1: Annual all-cause mortality rates for the general population

Age group (years)	Males (percent)	Females (percent)
<1	0.00441	0.00349
1-8	0.00014	0.00012
9-11	0.00008	0.00008
12	0.00009	0.00007
13	0.00013	0.00010
14-17	0.00021	0.00012
18	0.00039	0.00018
19	0.00046	0.00017
20-24	0.00046	0.00021
25-26	0.00058	0.00025
27-29	0.00061	0.00031
30-34	0.00079	0.00043
35-39	0.00119	0.00066
40-44	0.00172	0.00103
45-49	0.00248	0.00156
50-54	0.00368	0.00248
55-59	0.00592	0.00396
60-64	0.00961	0.00614
65-69	0.01434	0.00944
70-74	0.02448	0.01605
75-79	0.04074	0.02809
80-84	0.07318	0.05326
>85	0.16238	0.14371

3.4.2 Sexual behavioral data

The sexual behavioral data was extracted from NATSAL III (the third National Survey of Sexual Attitudes and Lifestyles) study.[22] NATSAL III is a stratified probability sample survey of 15,162 men and women aged 16-74 years in 2010. Among a range of questions about respondents' sexual lifestyles and attitudes were items about their history of diagnosed sexually transmitted infections. As results were not reported the way the model required it (e.g., mean number of partners over lifetime and not last year), NATSAL authors were contacted, and they provided additional data to allow us to inform the model. Results are reported in **Table 2**, **Table 3,** and **Table 4**.

Table 2: Percent of the population in each of the following sexual activity categories

Category	Male	Female
Low (mean number of sexual partners/year: ≤ 1)	85.1	90.7
Medium (mean number of sexual partners/year: 2-4)	11.9	7.6
High (mean number of sexual partners/year: 5+)	3	1.7

Table 3: Mean number of sexual partners per year by activity category and gender

Sexual activity category	Males (number)	Females (number)
Low (mean number of sexual partners per year: 0-1)	0.79	0.75
Medium (mean number of sexual partners per year: 2-4)	2.54	2.52
High (mean number of sexual partners per year: 5+)	9.80	9.66

Table 4: Mean number of sexual partners per year by age group and gender

Age group	Male	Female
13-14*	0.0001	0.0001
15*-19	1.70	1.40
20-24	2.00	1.60
25-29	1.70	1.30
30-34	1.50	1.20
35-39	1.20	1.00
40-44	1.10	1.50
45-49	1.10	1.00
50-54	1.10	0.90
55-59	1.00	0.70
60-64	0.90	0.60
65-69	0.80	0.50
70-74	0.50	0.30
75+*	0.5	0.30

The amount of sexual mixing among members of different age cohorts (a value between 0 and 1 with 0 representing no mixing, and 1 representing maximum mixing) and the amount of sexual mixing among members of different sexual activity groups required in the model were extracted from the technical report accompanying the Manuscript "Impact of Vaccinating Boys and Men against HPV in the United States".[23] Sexual mixing values are reported in **Table 5**.

Table 5: Sexual mixing

Ages	Sexual mixing
Among members of different age cohort	
Between debut and cessation	0.40
After cessation	0.10
Among members of different sexual activity groups	0.50

3.5 Screening parameters

3.5.1 Cervical cancer screening information

The percentage of females receiving a follow-up screening test after an abnormal cytology result was estimated from the cervical screening programme 2018-2019 report published by the Health and Social Care Information Centre using the following formula:

Percentage of females receiving a follow-up screening after an abnormal cytology result

$$= \frac{\textit{Women tested again for abnormality + Women referred to colposcopy for abnormal result}}{\textit{Number of women with abnormal test}}$$

The number of women referred to colposcopy for abnormal results was estimated by multiplying the number of women referred to colposcopy for a first attendance in 2013-14 (188,817) by the percentage of referrals for colposcopy reported as being triggered by an abnormal result of screening test (67.6%). Results are reported in **Table 6**. An estimate of the percentage of females who never received gynecological cancer screening in their lifetime was directly retrieved from cervical screening program 2013-2014 report and is also reported in **Table 6**. [24,25]

Table 6: Cervical cancer screening information

Cancer screening information	Value females (percent)
Percent of females receiving a follow-up screening test after abnormal cytology result	70.52
Percent of females never receiving a gynaecological screening in their lifetime.	7.0

The percentage of females screened for cervical cancer in the past year was calculated using the numbers of women tested in the year 2013-2014 reported by the Health and Social Care Information Centre and mid-2013 women estimates for England by age from the ONS, which were compared with 2019 screening data to confirm that there were no significant changes in the screening rates.[24,25]

Results are shown in **Table 7**.

Table 7: Percentage of females screened for cervical cancer in the past year

Age group (years)	Value females (percent)
<1	0.01
1-8	0.01
9-11	0.01
12	0.01
13	0.01
14-17	0.01
18	0.01
19	0.01
20-24	2.48
25-26	31.05
27-29	31.05
30-34	27.20
35-39	26.80
40-44	25.27
45-49	24.28
50-54	18.74
55-59	16.28
60-64	8.88
65-69	1.65
70-74	0.25
75-79	0.04
80-84	0.04
>85	0.04

Table 8 summarises the values of the parameter related to the diagnostic performance of cytological screening and colposcopy for cervical disease and/or CIN stage. The specificity and sensitivity of cytological screening and colposcopy for cervical disease are kept the same as the US data.

Table 8: Diagnostic performance of screening tests by cervical disease (international)

Parameter	Cervical disease	CIN 1	CIN 2	CIN 3
Cytology specificity	0.94	Not considered		
Colposcopy sensitivity	0.96			
Colposcopy specificity	0.48			
Cytology sensitivity	Not considered	0.28	0.59	0.59

Source: Elbasha and Dasbach[26]

Because the UK has no vaginal cancer screening program, the percentage of females receiving regular vaginal cancer screening will be set to 0.

3.6 Natural history of disease

Tables 9-17 show the calibrated values of model parameters, in particular those that are most relevant to the dynamics of HPV infection, e.g., transmission and HPV infection progress, recovery, and natural immunity. Values of these different parameters are similar to US values or were estimated through a calibration process.

Table 9: Probability of transmitting HPV infection per sexual partnership, by anatomical site and HPV genotype

Site	Transmission	HPV 6	HPV 11	HPV 16	HPV 18	HPV 31	HPV 33	HPV 45	HPV 52	HPV 58
Cervical	Same	-	-	0.6576	0.7095	0.8602	0.7947	0.7477	0.5806	0.8057
Vaginal	To females	-	-	0.5473	0.2410	0.2769	0.3008	0.2796	0.2138	0.2248
	To males	-	-	0.2262	0.1525	0.1630	0.1539	0.0529	0.2138	0.1848
Vulvar	Same	-	-	0.4399	0.0711	0.1562	0.1562	0.0989	0.1013	0.0988
Anal	To females	-	-	0.1511	0.1421	0.0546	0.0773	0.0804	0.0700	0.0917
	To males	-	-	0.1502	0.1709	0.0600	0.0889	0.0832	0.0713	0.0800
Head and neck	To females	-	-	0.0606	0.0878	0.0878	0.0878	0.0878	0.0453	0.0878
	To males	-	-	0.0358	0.0335	0.0335	0.0335	0.0338	0.0346	0.0342
Penile	To females	-	-	0.8112	0.7632	0.1749	0.1709	0.2145	0.1363	0.1470
	To males	-	-	0.5947	0.5848	0.1072	0.2614	0.2352	0.1831	0.1943
Genital warts	To females	0.30	0.12	-	-	-	-	-	-	-
	To males	0.34	0.09	-	-	-	-	-	-	-

Source: Model calibration.

Table 10: Recurrence rate of treated CIN by stage

Stage	Rate
CIN 1	0.05
CIN 2	0.05
CIN 3	0.05

Table 11: Rate of cancer progression (same for all diseases)

Progression	Rate
Local to regional	0.1
Regional to distant	0.3

Table 12: Parameters of natural history of cervical disease

Parameters (references)	HPV 16	HPV 18	HPV 31	HPV 33	HPV 45	HPV 52	HPV 58
Fraction of persistent cervical HPV infections*	0.0812	0.0166	0.0274	0.0119	0.0125	0.0266	0.0115
Clearance rate of cervical HPV infections*							
Male	0.5780	0.5191	0.5962	0.3656	0.3656	0.3656	0.3656
Female	0.7722	0.6711	0.7159	0.9438	0.9336	0.9221	0.9491
Fraction of people seroconvert following a cervical HPV infection**							
Male	0.0866	0.0661	0.0941	0.0900	0.0900	0.0900	0.0900
Female	0.5928	0.5076	0.8169	0.5000	0.5000	0.5000	0.5000
Degree of protection against cervical HPV infections provided by natural immunity following seroconversion**							
Male	0.1775	0.1664	0.1408	0.1664	0.1664	0.1664	0.1664
Female	0.3708	0.4968	0.3048	0.5000	0.5000	0.5000	0.5000

Table 13: Parameters for natural history of vaginal disease

Parameters (references)	HPV 16	HPV 18	HPV 31	HPV 33	HPV 45	HPV 52	HPV 58
Fraction of persistent vaginal HPV infections*	0.0301	0.1441	0.0206	0.0301	0.1048	0.0177	0.0186
Clearance rate of vaginal HPV infections**							
Male	0.6667	0.6667	0.6667	0.6667	0.6667	0.6667	0.6667
Female	0.5034	0.5034	0.5034	0.5034	0.5034	0.5034	0.5034
Fraction of people seroconvert following a vaginal HPV infection*							
Male	0.0405	0.0599	0.0594	0.0658	0.0694	0.0289	0.0134
Female	0.5438	0.6082	0.5960	0.6201	0.5835	0.5300	0.5205
Degree of protection against vaginal HPV infections provided by natural immunity following seroconversion*							
Male	0.4112	0.3363	0.3537	0.3021	0.3405	0.1126	0.1591
Female	0.1013	0.1089	0.2282	0.2555	0.2578	0.3099	0.3141

*From model calibration.
** From model calibration for the US.

Table 14: Parameters for natural history of vulvar disease

Parameters (references)	HPV 16	HPV 18	HPV 31	HPV 33	HPV 45	HPV 52	HPV 58
Fraction of persistent vulvar HPV infections*	0.0185	0.0290	0.0100	0.0100	0.0283	0.0163	0.0228
Clearance rate of vulvar HPV infections*							
Male	0.6585	0.1200	0.1494	0.1494	0.1673	0.1548	0.1639
Female	0.7162	0.1217	0.3200	0.3200	0.2641	0.3005	0.2972
Fraction of people seroconvert following a vulvar HPV infection**							
Male	0.0239	0.0239	0.0239	0.0239	0.0239	0.0239	0.0239
Female	0.3569	0.3569	0.3569	0.3569	0.3569	0.3569	0.3569
Degree of protection against vulvar HPV infections provided by natural immunity following seroconversion**							
Male	0.3486	0.3486	0.3486	0.3486	0.3486	0.3486	0.3486
Female	0.3277	0.3277	0.3277	0.3277	0.3277	0.3277	0.3277

*From model calibration.
** From model calibration for the US.

Table 15: Natural history parameters for anal disease

Parameters (references)	HPV 16	HPV 18	HPV 31	HPV 33	HPV 45	HPV 52	HPV 58
Fraction of persistent anal HPV infections*							
Male	0.1272	0.1340	0.1434	0.1189	0.0968	0.0861	0.1448
Female	0.1273	0.1137	0.1128	0.1300	0.0950	0.0830	0.1176
Clearance rate of anal HPV infections*							
Male	0.3000	0.3500	0.4824	0.4960	0.5042	0.4627	0.4983
Female	0.2500	0.4891	0.7738	0.6124	0.7006	0.7040	0.7197
Fraction of people seroconvert following an anal HPV infection**							
Male	0.2500	0.2500	0.2500	0.2500	0.2500	0.2500	0.2500
Female	0.6000	0.6000	0.6000	0.6000	0.6000	0.6000	0.6000
Degree of protection against anal HPV infections provided by natural immunity following seroconversion**							
Male	0.0496	0.0496	0.0496	0.0496	0.0496	0.0496	0.0496
Female	0.3938	0.3938	0.3938	0.3938	0.3938	0.3938	0.3938

*From model calibration.
** From model calibration for the US.

Table 16: Natural history parameters for head and neck cancer

Parameters (references)	HPV 16	HPV 18	HPV 31	HPV 33	HPV 45	HPV 52	HPV 58
Fraction of persistent head and neck HPV infections*							
Male	0.0860	0.0193	0.0193	0.0193	0.0193	0.0836	0.0193
Female	0.0860	0.0121	0.0121	0.0121	0.0121	0.0686	0.0121
Clearance rate of head and neck HPV infections*							
Male	0.3230	0.3433	0.3588	0.3588	0.3588	0.3213	0.3581
Female	0.3632	0.3848	0.3848	0.3810	0.3734	0.4264	0.3867
Fraction of people seroconvert following a head and neck HPV infection**							
Male	0.1040	0.1040	0.1040	0.1040	0.1040	0.1040	0.1040
Female	0.6048	0.6048	0.6048	0.6048	0.6048	0.6048	0.6048
Degree of protection against head and neck HPV infections provided by natural immunity following seroconversion*							
Male	0.1159	0.1159	0.1159	0.1159	0.1159	0.1159	0.1159
Female	0.2012	0.2012	0.2012	0.2012	0.2012	0.2012	0.2012

*From model calibration.
** From model calibration for the US.

Table 17: Natural history parameters for penile cancer

Parameters (references)	HPV 16	HPV 18	HPV 31	HPV 33	HPV 45	HPV 52	HPV 58
Fraction of persistent penile HPV infections*							
Male	0.2667	0.1933	0.1971	0.1967	0.1572	0.0804	0.1869
Clearance rate of penile HPV infections**							
Male	0.2386	0.2335	0.2900	0.4171	0.5723	0.4090	0.4460
Female	0.6303	0.2747	0.5247	0.5387	0.5039	0.5116	0.6297
Fraction of people seroconvert following a penile HPV infection**							
Male	0.1040	0.1040	0.1040	0.1040	0.1040	0.1040	0.1040
Female	0.6048	0.6048	0.6048	0.6048	0.6048	0.6048	0.6048
Degree of protection against penile HPV infections provided by natural immunity following seroconversion**							
Male	0.1159	0.1159	0.1159	0.1159	0.1159	0.1159	0.1159
Female	0.2012	0.2012	0.2012	0.2012	0.2012	0.2012	0.2012

*From model calibration.
** From model calibration for the US.

4 Treatment Patterns

The female population receiving hysterectomy over the course of 1 year was estimated from Hospital episodes statistics (HES) records 2018 published by Health & Social Care Information Centre.[27] The following procedure codes were used:

Q07.1: Abdominal hysterocolpectomy and excision of periuterine tissue
Q07.2: Abdominal hysterectomy and excision of periuterine tissue NEC
Q07.3: Abdominal hysterocolpectomy NEC
Q07.4: Total abdominal hysterectomy NEC
Q07.8: Vaginal hysterocolpectomy and excision of periuterine tissue
Q08.1 Vaginal hysterocolpectomy and excision of periuterine tissue
Q08.2 Vaginal hysterectomy and excision of periuterine tissue NEC
Q08.3 Vaginal hysterocolpectomy NEC
Q08.8: Other specified vaginal excision of uterus
Q08.9: Unspecified vaginal excision of uterus

R25.1: Caesarean hysterectomy
X14.1: Total exenteration of pelvis
X14.2: Anterior exenteration of pelvis
X14.3: Posterior exenteration of pelvis
X14.8: Other specified clearance of pelvis
X14.9: Unspecified clearance of pelvis

The total number of hysterectomies by age obtained from NHS Hospital episode statistics for admitted patient care in 2013-2014 was divided by the sum of women by age group in England. Results are reported in **Table 18**.

Table 18: Women receiving hysterectomy over the course of 1 year

Age group (years)	Value females (%)
<1	0.0000
1-8	0.0001
9-10	0.0001
11-12	0.0001
13-14	0.0001
15-17	0.0002
18	0.0003
19	0.0012
20-24	0.0032
25-26	0.0193
27-29	0.0193
30-34	0.0587
35-39	0.1384
40-44	0.2910
45-49	0.3794
50-54	0.2504
55-59	0.1796
60-64	0.1744
65-69	0.1876
70-74	0.1761
75-79	0.1569
80-84	0.1088
>85	0.0435

The parameters related to the percent of precancerous lesions (Cervical Intraepithelial Neoplasia (CIN), Vaginal Intraepithelial Neoplasia (VaIN) and Vulval Intraepithelial Neoplasia (VIN)) as well as Carcinoma In Situ (CIS) which are treated were estimated through model calibration. This also applies to the parameters related to the percentage of females with cancer recognising their symptoms and seeking treatment. Table 19 summarises the values of the parameter related to the percent of precancerous lesions (CIN and VaIN) as well as CIS which are treated by stage. It was estimated through model calibration that 66.4% of genital warts were treated in males and 51.4% in females.

Table 19: Treatment patterns of all stages of diseases

**Cervical cancer stage**	**Percent seeking treatment**	**Vaginal/vulvar cancer stage**	**Percent seeking treatment**	**Anal cancer stage**	**Value males (%)**	**Value females (%)**
Local disease	4.0	Local disease	3.8	Local disease	3.0	5.0
Regional disease	18.0	Regional disease	18.0	Regional disease	95	95
Distant disease	95.0	Distant disease	90.0	Distant disease	95	95

**Penile cancer stage**	**Value males (%)**	**Head and neck cancer stage**	**Value males (%)**	**Value females (%)**
Local disease	5.0	Local disease	5.0	5.0
Regional disease	25.0	Regional disease	25.0	25.0
Distant disease	90.0	Distant disease	90.0	90.0

**Cervical disease stage**	**Percent treated**	**Vaginal disease stage**	**Percent treated**
CIN 1	50	VaIN 1	30
CIN 2	100	VaIN 2/3	100
CIN 3	100	CIS	100
CIS	100		

5 Cancer Mortality

The model requires HPV-related cancers associated mortality rates stratified by age and stage (local, regional, distant). To estimate HPV-related cancer-associated mortality rates, we used survival data from the Cancer Research UK website and Eurocare 5 database.[28,29] EUROCARE (European Cancer Registry) is a collaborative research project on cancer survival in Europe. EUROCARE-5 includes data on more than 21 million cancer diagnoses provided by 116 Cancer Registries in 30 European countries over the period 1999-2007. Summary results of the EUROCARE-5 study of cancer survival in Europe were published in 2013.137 Survival data by age from this study were extracted for the UK and Ireland together for the following cancers: vaginal, vulval, anal, penile, and H&N (data for the UK alone was not available).

Since data from EUROCARE 5 was only available from the age of 15, cancer-associated mortality rates were assumed to be 0 for the population below 15.

Since survival data were available either by age or by stage, an extrapolation to reconcile the age-stratified and stage-stratified data into one table as required by the model has been performed.

The calculation of cervical cancer mortality is described below for illustrative purposes.
As a first step, the following two tables (**Tables 20 and 21**) were extracted from the Cancer Research UK website.[30]

Table 20:Cervical cancer survival by age (2007-2011)

Age range	5-year net survival (%)
15-39	89.6
40-49	78.9
50-59	67.7
60-69	54.7
70-79	38.5
80-99	25.4

Table 21: Five-year relative survival by stage (2002-2006)

Age range	5-year relative survival (%)
Stage I	95.9
Stage II	54.4
Stage III	37.9
Stage IV	5.3

Summary cancer stages and TNM stages were matched as follows:

Table 22: Correspondence between TNM and summary stage

Summary stage	TNM stage
Local	Stage I and II (50%/50%)
Regional	Stage III
Distant	Stage IV

From Tables 20 and 21, the five-year mortality was estimated using the formula:

$$\textit{5year mortality} = \frac{\textit{100-5year survival}}{100}$$

Then, the annual mortality was estimated, assuming an exponential survival curve, using the formula:

$$\textit{1year mortality} = \textit{1-EXP}\left(\frac{\textit{LN(1-5year mortality}}{5}\right)$$

From the mortality by stage, a relative risk of each stage was calculated using regional cancer mortality as the reference case. It was assumed that the average age-stratified mortality represents the situation of regional cancer and the relative risk was applied to extrapolate the mortality data by age and stage. The final table is given below (Table 23). A similar methodology was adopted to calculate the mortality rates of vulval, vaginal, anal, head and neck, and penile cancer. Head and neck cancer consist of a variety of different types and due to data availability, oral and larynx cancer mortality were used in the calculation. UK-specific age-stratified mortality for penile cancer was not available, and testis cancer mortality by age was used as an approximation. The mortality of RRP was low and thus was assumed to be the same between US and UK (1% annually).[31]

Table 23: Annual cancer-associated mortality by site, age and stage

Cancer type	Age group (years)	Mortality rate		
		Local cancer	Regional cancer	Distant cancer
Cervical cancer	0-14	0.000	0.000	0.000
	15-39	0.007	0.022	0.055
	40-49	0.015	0.046	0.117
	50-59	0.024	0.075	0.189
	60-69	0.036	0.114	0.286
	70-79	0.055	0.174	0.438
	>80	0.075	0.240	0.604
Vaginal cancer	0-14	0	0	0
	15-44	0.021	0.036	0.064
	45-54	0.034	0.058	0.103
	55-64	0.053	0.091	0.163
	65-74	0.072	0.124	0.220
	>75	0.125	0.216	0.384
Vulvar cancer	0-14	0	0	0
	15-44	0.017	0.036	0.078
	45-54	0.027	0.058	0.125
	55-64	0.042	0.091	0.198
	65-74	0.057	0.124	0.267
	>75	0.099	0.216	0.466
Anal cancer (females)	0-14	0	0	0
	15-54	0.038	0.084	0.144
	55-64	0.041	0.092	0.157
	65-74	0.056	0.124	0.213
	>75	0.101	0.224	0.386
Anal cancer (males)	0-14	0	0	0
	15-44	0.043	0.096	0.165
	45-54	0.048	0.106	0.183
	55-64	0.052	0.115	0.198
	65-74	0.065	0.144	0.248
	>75	0.107	0.237	0.407
Penile cancer	0-14	0	0	0
	15-44	0.011	0.052	0.115
	45-54	0.010	0.049	0.107

Cancer type	Age group (years)	Mortality rate		
		Local cancer	Regional cancer	Distant cancer
	55-64	0.013	0.064	0.140
	65-74	0.020	0.093	0.205
	>75	0.042	0.199	0.438
Head & neck cancer (females)	0-14	0	0	0
	15-44	0.040	0.058	0.072
	45-54	0.063	0.091	0.114
	55-64	0.083	0.120	0.149
	65-74	0.099	0.143	0.179
	>75	0.165	0.239	0.298
Head & neck cancer (males)	0-14	0	0	0
	15-44	0.056	0.081	0.101
	45-54	0.078	0.114	0.142
	55-64	0.104	0.151	0.188
	65-74	0.141	0.204	0.255
	>75	0.193	0.279	0.349

6 Vaccine Properties

The prophylactic efficacy of the vaccine or vaccine degree of protection is based on clinical trial data. [10,32-36] The duration of protection against HPV genotypes contained in the vaccines is assumed to be lifelong. No cross-protection effect was considered in this analysis.

6.1 Efficacy against infection vs efficacy against disease

The model considers different efficacy values as the property of the model includes a degree of protection against infection and a degree of protection against disease given a breakthrough infection. In the model it is further assumed that these "breakthrough" infections are transmissible.

6.2 Efficacy against non-cervical cancers

The efficacy against anal, head and neck, penile and RRP diseases is conferred through protection against infection only.

6.3 Efficacy for less than 3 doses

In the absence of data, the efficacy for 1 dose was assumed to be 0% and efficacy for 2 doses 100%. Vaccines should have a very similar clinical effect between the US and UK, and thus the following variables are the same for all countries:

- The existence of herd immunity
- The relative effectiveness of the vaccine if fewer than the full regimen of three doses are received
- Duration of protection
- Relative risk of people with breakthrough genital HPV infections transmitting the infection to their partners

- Efficacy of the vaccine against HPV infection
- Degree of protection of the vaccine against HPV infections becoming persistent

Vaccine efficacy assumptions are reported in **Table 24** for HPV-related cancers and in **Table 25** for HPV-related genital warts.

Table 24: Vaccine efficacy assumptions (international) for HPV-related cancers

**Vaccine assumptions**	**HPV 16**	**HPV 18**	**HPV 31, 33, 45, 52 and 58**
Cervical cancer			
Male*	0.411	0.411	0.411
Female	0.76	0.76	0.76
Degree of protection of the vaccine against cervical HPV infections becoming persistent	0.988	0.988	0.988
Degree of protection of the vaccine against HPV-related CIN	0.97	0.97	0.97
Vaginal and vulvar cancers			
Vaccine efficacy for preventing vaginal/vulvar HPV infections			
Male*	0.411	0.621	0.621
Female	0.76	0.963	0.963
Degree of protection of the vaccine against vaginal/vulvar HPV infections becoming persistent	0.988	0.984	0.984
Degree of protection of the vaccine against HPV-related /VaIN/VIN	1	1	1
Anal cancers			
Vaccine efficacy for preventing anal HPV16/18 infections			
Male	0.762	1	0.762
Female	0.762	1	0.762
Degree of protection of the vaccine against anal HPV infections becoming persistent			
Male	0.938	0.999	0.938
Female	0.938	0.999	0.938
Degree of protection of the vaccine against HPV-related AIN	0.655	1	0.655
H&N cancers			
Vaccine efficacy for preventing H&N infections			
Male	0.411	0.621	0.621
Female	0.760	0.963	0.963
Degree of protection of the vaccine against H&N infections becoming persistent			
Male	0.787	0.96	0.96
Female	0.988	0.984	0.984
Degree of protection of the vaccine against HPV-related H&N neoplasia	0	0	0
Penile cancer			
Vaccine efficacy for preventing penile HPV infections			
Male	0.411	0.621	0.621
Female	0.760	0.963	0.963
Degree of protection of the vaccine against penile HPV16/18 infections becoming persistent			
Degree of protection of the vaccine against HPV-related PIN	0.787	0.960	0.960

*Preventing male genital infections through male vaccination is assumed to prevent transmission of genital infections to females.
**Preventing female genital infections through vaccination is assumed to prevent transmission of genital infections to males. CIN: *Cervical intraepithelial neoplasia, VaIN: vaginal intraepithelial neoplasia, VIN: vulval intraepithelial neoplasia, AIN: anal intraepithelial neoplasia, PIN: penile intraepithelial neoplasia*
Giuliano et a[34] for males; Garland et al[37] and Palefsky et al[36] for females.

Table 25: Vaccine efficacy assumption (international) for genital warts

Vaccine assumptions	HPV 6	HPV 11
Vaccine efficacy against HPV 6/11 infection		
Females	0.761	0.761
Males	0.49	0.57
Degree of protection of the vaccine against HPV 6/11-related genital warts		
Females	0.989	1
Males	0.843	0.909
Degree of protection of the vaccine against HPV 6/11-related CIN 1	1	1

Source: Giuliano et al[34] for males; Garland et al[37] and Palefsky et al[36] for females.

7 Vaccination Strategy

Data on annual HPV quadrivalent vaccine coverage in England, Wales and Scotland in 2018/2019 were used to estimate vaccination coverage rate in the model. [38-40] shown below in **Table 26**.

It is assumed that, in the strategy of universal vaccination, the same coverage rate will be achieved for males and females.

It is assumed that the coverage of 9vHPV will be the same as with 4vHPV.

Table 26: Coverage of HPV vaccination programme for girls (from Datta et al (2019)[78])

Year	Vaccine	Routine Uptake (%)	13	14	15	16	17
2008-2009	Bivalent	80.9	0	0	0	0	47.4
2009-2010	Bivalent	77.5	0	68.5	68.6	41.7	38.9
2010-2011	Bivalent	83.8	4.5	0.3	7.2	2.2	6.4
2011-2012	Quadrivalent	87.0	0	0	0	0	0
2012-2013	Quadrivalent	85.8	0	0	0	0	0
2013-2014	Quadrivalent	88.1	0	0	0	0	0
2014-2015	Quadrivalent	87.5	0	0	0	0	0
2015-2016	Quadrivalent	85.1	0	0	0	0	0

Table 27 summarises the values of the parameter related to the percent of young females receiving the full three doses of the vaccine (*i.e.,* adherence or compliance). The percentages refer to the proportion of those who receive the first dose receiving a second dose, and the proportion of those who receive the second dose receiving a third dose within one year of initiating vaccination. The proportion of those receiving the third dose was set to 0% since the target vaccination age range (12-13 years) only requires two doses to receive the full benefit of the vaccine.

Table 27: Percentage of individuals receiving two-or three-dose vaccine

Adherence	Value
Get second dose after first dose	95%
Get third dose after second dose	0%

8 Costs

To be consistent, all cost values from the different sources were inflated to 2019/2020 British pounds (£) and discounted at 3.5% and 1.5% accordingly to the NICE recommendations.

8.1 Cost of vaccine administration

The administration cost per dose (£10) from Datta et al (2019).[9]

8.2 Cost of vaccination

Cost of a single dose of 4vHPV vaccine was £17.80 retrieved from the British National Formulary (BNF) and a dose of 9vHPV was varied by scenario.

8.3 Cost per episode of care

The cost per episode of care of RRP was obtained from a publication from Jit et al. where separate costs for juvenile and adult cases were reported.[7] As the model does not allow for a difference in costs according to the age of patients, an average of the two was used.

All costs per episode of care are reported in **Table 28**.

Table 28: Costs of diagnosing and treating diseases caused by HPV infection

Parameter	Gender	Original values	Inflated values (£2019/2020)
CIN 1, 2, 3 and CIS[7,41,42]	Female	£349	£406
Cervical cancer, local disease[43]	Female	£18,425	£20,701
Cervical cancer, regional disease[43]	Female	£22,780	£25,594
Cervical cancer, distant disease[43]	Female	£24,244	£27,239
VaIN 1, 2, 3 and CIS[7,41]	Female	£349	£406
Vaginal cancer, all stages[7]	Female	£13,650	£15,893
Vulval cancer, all stages[7]	Female	£13,650	£15,893
Penile cancer, all stages[44]	Male	£8,063	£10,482
Anal cancer, all stages[45]	Male/Female	£16,281	£18,292
Head & neck cancer, all stages[7]	Male/Female	£15,000	£17,465
Genital warts[46]	Male/Female	£265	£292
Recurrent respiratory papillomatosis[47]	Male/Female	£17,450	£18,981

* Disease stages can be related to the traditional Tumour-Node-Metastasis (TNM) classification system as followed: - "Local disease" corresponds to stages I and II TNM classification, i.e., localized primary tumour; "Regional disease" corresponds to stage III TNM classification system, i.e., metastasis to regional lymph nodes; "Distant disease" corresponds to stage IV TNM classification system, i.e., distant metastatic disease.

8.4 Cost of screening and diagnostic tests

The costs of screening and diagnostic tests were retrieved from Jit et al. and a report from NICE.[7,43,48] The different costs are reported in **Table 29**.

Table 29: Screening and diagnostic tests for cervical and vaginal cancers

Parameter	Value (females, £2019/2020)
Screening (cytology) and office visit[7]	£58
Screening (HPV)[49]	£25.06
Colposcopy[43,48]	£151.18
Biopsy [43,48]	£79.84

9 Health-Related Quality of Life

A discount rate for both costs and benefits of 3.5% and 1.5% were used, accordingly to the NICE recommendations. Age-specific health utility values in the healthy population were based on a UK-specific catalogue of EQ-5D scores from Sullivan et al[50] shown in tables below.

Table 30: Age-specific utilities in healthy population

Age group (year)	Male Utility	Female Utility
1-17	0.93	0.93
18-34	0.92	0.91
35-44	0.90	0.89
45-54	0.87	0.86
55-64	0.81	0.80
65-74	0.76	0.78
75+	0.69	0.70

Unit: number between 0 and 1

Table 31: Utilities in population with HPV-related diseases

Condition	Value
CIN1, VaIN1, VIN1	0.91
CIN2+, CIS, VaIN2+, VIN2+	0.87
Cervical/Vaginal/Vulvar/Anal/H&N/Penile Cancer, Local	0.76
Cervical/Vaginal/Vulvar/Anal/H&N/Penile Cancer, Regional	0.67
Cervical/Vaginal/Vulvar/Anal/H&N/Penile Cancer, Distant	0.48
Cervical/Vaginal/Vulvar/Anal/H&N/Penile Cancer, Survivor	0.76
Genital Warts	0.91
RRP	0.79

Sources: Calculated based on Sullivan et al (2011),[50] Dominiak-Felden et al (2013),[51] and Chadha et al (2010).[51]

Unit: Number between 0 and 1.

10 Calibration Process

10.1 Data sources

The model was calibrated utilizing cancer incidence data, HPV type attribution, HPV prevalence by type, and genital warts and RRP incidence. Cancer incidence data (with the exception of penile cancer) was retrieved from Cancer Research UK. Penile cancer incidence data was retrieved from the National Cancer Intelligence Network. The cancer incidence data is given in the tables below, stratified according to the ages in the model:

Table 32: Cancer incidence (cases per 100,000) data for females

Female	Cervical	CIN 1	CIN 2+	Vaginal	Vulvar	Anal	Oral cavity	Oropharynx	Larynx	Head & Neck	Genital warts
Overall	9.80	302.55	156.53	0.80	3.80	2.20	6.90	0.18	1.30	8.38	120.10
15-17	0.10	0.97	0.23	0.00	0.00	0.00	0.20	0.00	0.00	0.20	509.50
18	0.10	0.97	0.23	0.00	0.00	0.00	0.20	0.00	0.00	0.20	509.50
19	0.10	0.97	0.23	0.00	0.00	0.00	0.20	0.00	0.00	0.20	509.50
20-24	2.90	238.84	122.72	0.00	0.20	0.00	0.20	0.01	0.00	0.21	662.80
25-26	19.10	1527.96	985.75	0.10	0.20	0.10	0.50	0.00	0.00	0.50	222.90
27-29	19.10	1527.96	985.75	0.10	0.20	0.10	0.50	0.00	0.00	0.50	222.90
30-34	19.70	836.08	440.09	0.10	0.50	0.30	0.90	0.00	0.10	1.00	222.90
35-39	19.10	573.77	284.12	0.40	1.20	0.70	1.50	0.02	0.20	1.72	71.90
40-44	15.70	445.66	178.28	0.50	2.30	1.40	3.20	0.08	0.40	3.68	71.90
45-49	12.10	381.65	113.24	0.40	3.10	2.10	5.30	0.09	0.80	6.19	24.40
50-54	10.30	271.96	76.76	1.00	3.80	3.60	10.30	0.36	1.60	12.26	24.40
55-59	10.40	165.11	58.09	1.40	4.30	4.50	14.40	0.44	2.40	17.24	24.40
60-64	9.10	91.32	34.51	1.50	5.40	5.20	16.40	0.67	3.20	20.27	24.40
65-69	8.60	33.61	10.97	2.10	6.60	5.50	17.80	0.46	4.50	22.76	1.80
70-74	9.90	10.42	3.05	2.30	9.20	6.40	20.00	0.50	4.70	25.20	1.80
75-79	11.80	2.13	0.60	2.20	14.80	5.90	20.30	0.28	4.20	24.78	1.80
80-84	12.50	2.13	0.60	2.70	19.10	6.30	21.50	0.50	4.40	26.40	1.80
>85	12.40	2.13	0.60	4.90	23.70	7.30	23.60	0.47	3.20	27.27	1.80

Table 33: Cancer incidence (cases per 100,000) data for males

Male	Penile	Anal	Oral Cavity	Oropharynx	Larynx	Head & Neck	Genital Warts
Overall	1.30	1.40	14.10	0.52	6.20	20.82	154.90
15-17	0.00	0.00	0.20	0.00	0.00	0.20	229.10
18	0.00	0.00	0.20	0.00	0.00	0.20	229.10
19	0.00	0.00	0.20	0.00	0.00	0.20	229.10
20-24	0.00	0.00	0.20	0.00	0.00	0.20	803.30
25-26	0.00	0.00	0.80	0.00	0.00	0.80	388.50
27-29	0.00	0.00	0.80	0.00	0.00	0.80	388.50
30-34	0.20	0.20	1.10	0.00	0.00	1.10	388.50
35-39	0.20	0.40	2.80	0.05	0.50	3.35	127.50
40-44	0.60	1.00	6.70	0.17	1.30	8.17	127.50
45-49	1.10	1.40	15.00	0.69	3.10	18.79	47.50
50-54	1.90	2.00	27.00	1.11	6.80	34.91	47.50
55-59	2.50	2.70	39.50	1.37	13.60	54.47	47.50
60-64	4.00	3.20	42.50	1.79	17.60	61.89	47.50
65-69	4.90	4.10	41.50	1.54	23.10	66.14	8.10
70-74	6.40	4.40	39.00	1.34	26.20	66.54	8.10
75-79	7.50	4.50	36.10	1.27	25.20	62.57	8.10
80-84	9.00	5.80	34.40	1.24	24.00	59.64	8.10
>85	11.20	5.40	31.70	0.87	23.50	56.07	8.10

HPV type attribution was obtained from a series of global meta-analyses.[52-57] Often the data in these studies was aggregated across geographical regions, and where possible attribution for Europe was used. In some cases (for example, with anal and head and neck cancers) the data was aggregated either by sex or by geographical region, but not by both. In such cases, we used the attribution given by sex. Furthermore, in the case of head and neck cancers, the model does not contain multiple sites (oropharynx, larynx, etc.), but the attribution data will tend to be different across these sites. To account for this, we took the weighted average of the attributions for the oropharynx, larynx, and oral cavity. The weights that we used were the percentage of the overall incidence of head and neck cancers in these sites. The attributions are summarized in the tables below.

Table 34: HPV type attributions for male disease

Male	HPV 6	HPV 11	HPV 16	HPV 18	HPV 31	HPV 33	HPV 45	HPV 52	HPV 58
Penile	0	0	0.501000	0.161000	0.021000	0.035000	0.055000	0.018000	0.018000
Head and neck	0	0	0.042203	0.001381	0.000486	0.000670	0.001237	0.002155	0.000833
Anal	0	0	0.642026	0.076230	0.009317	0.019841	0.007623	0.004235	0.015246
Genital warts	0.740893	0.159107	0	0	0	0	0	0	0

Table 35: HPV type attribution for female disease

Female	HPV 6	HPV 11	HPV 16	HPV 18	HPV 31	HPV 33	HPV 45	HPV 52	HPV 58
Cervical	0	0	0.501000	0.161000	0.021000	0.035000	0.055000	0.018000	0.018000
CIN 1	0.046000	0.013400	0.086000	0.049000	0.064000	0.033000	0.037000	0.062000	0.024000
CIN 2+	0.016000	0.003000	0.456000	0.038000	0.096000	0.026000	0.015000	0.074000	0.042000
Vaginal	0	0	0.501000	0.161000	0.021000	0.035000	0.055000	0.018000	0.018000
Vulvar	0	0	0.501000	0.161000	0.021000	0.035000	0.055000	0.018000	0.018000
Anal	0	0	0.682200	0.030600	0.009900	0.020700	0.008100	0.004500	0.016200
Head and neck	0	0	0.052728	0.001839	0.000675	0.000961	0.001723	0.002620	0.001069
Genital warts	0.627289	0.134711	0	0	0	0	0	0	0

Pre-vaccination HPV prevalence by type was collected from Johnson et al (2012).[58] The prevalence for type 31, 33, 45, 52, and 58 were averaged together. The data used is found below.

Table 36: Prevalence of HPV in Britain

HPV type	Prevalence
16	0.042
18	0.014
31	0.006
33	0.009
45	0.007
52	0.023
58	0.017

Genital warts and RRP data were collected from Donne et al (2016)[59] and are given below (**Table 37**). We had a single estimate for the overall prevalence of RRP in the UK at 1.42 per 100,000.

Table 37: Genital warts incidence (cases per 100,000) in the UK

Age	15-19	20-24	25-34	35-44	45-64	65+
GW male	229.1	803.3	338.5	127.5	47.5	8.1
GW female	509.5	662.8	222.9	71.9	24.4	1.8

11 Calibration Results

The model was fit to the data utilizing Bayesian history matching as in previous HPV models.[60] Maximum likelihood fits were selected, and the plots of the fits against the data are given below for the various infection sites/diseases.

11.1 Anal

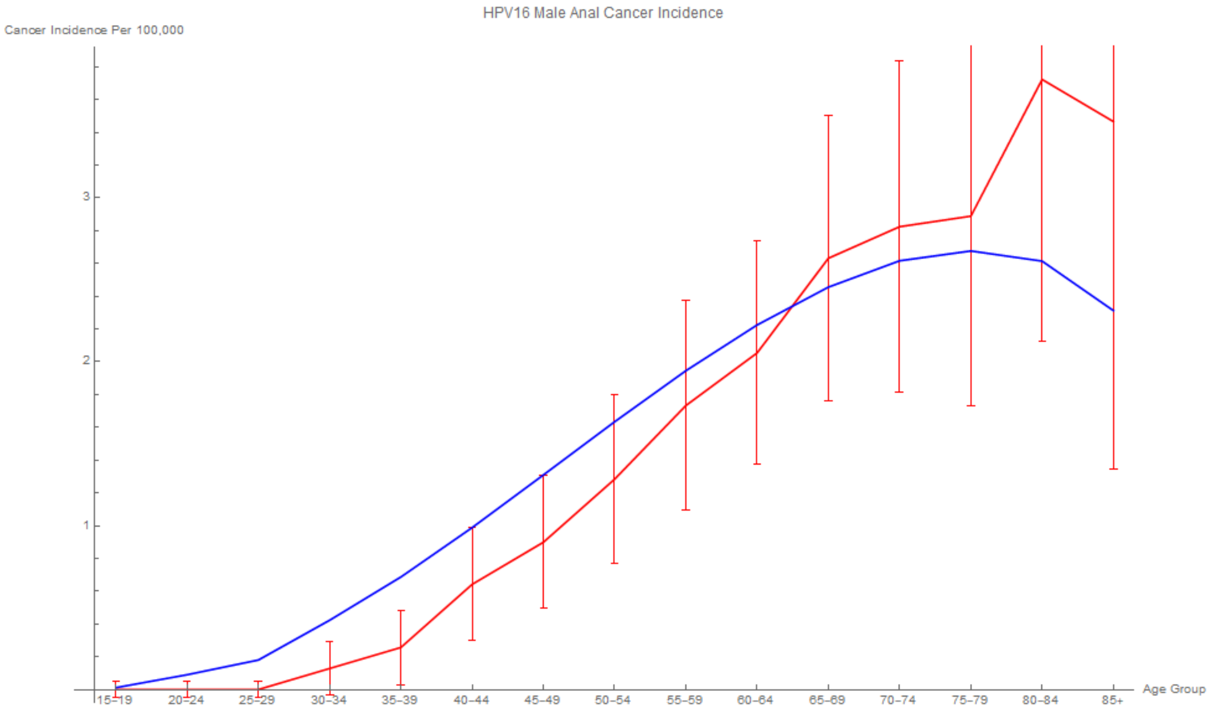

Figure 2. HPV 16 male anal cancer incidence

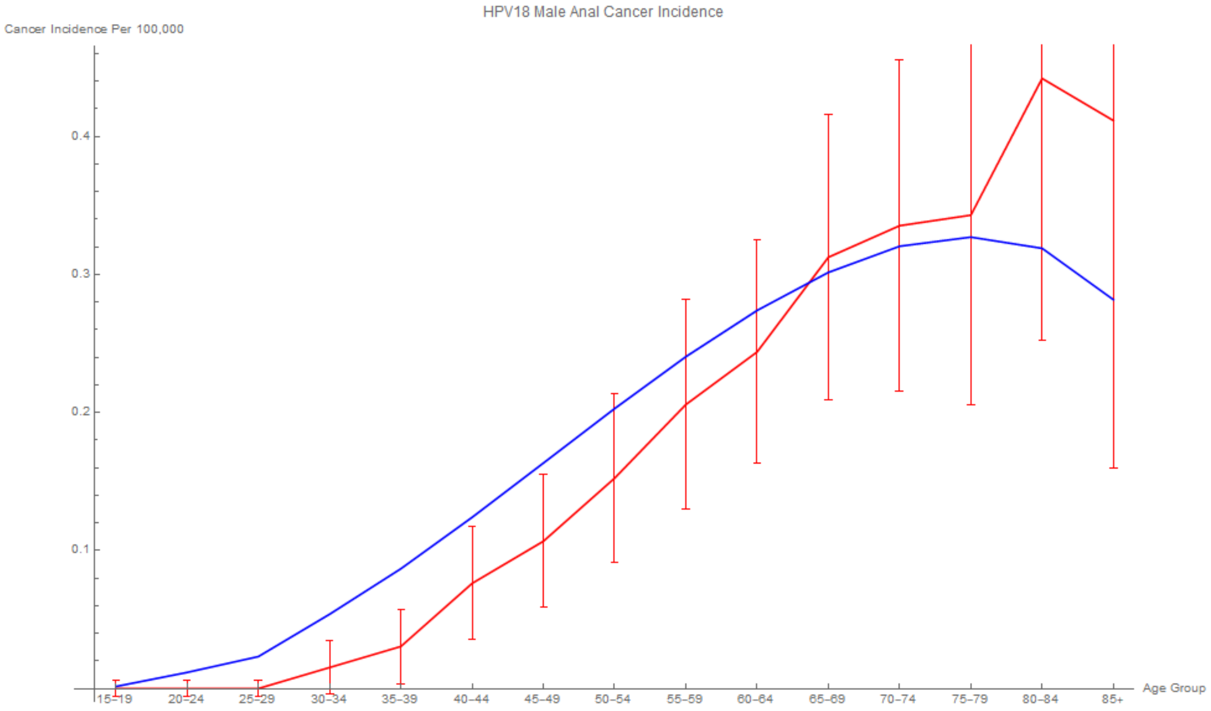

Figure 3. HPV 18 male anal cancer incidence

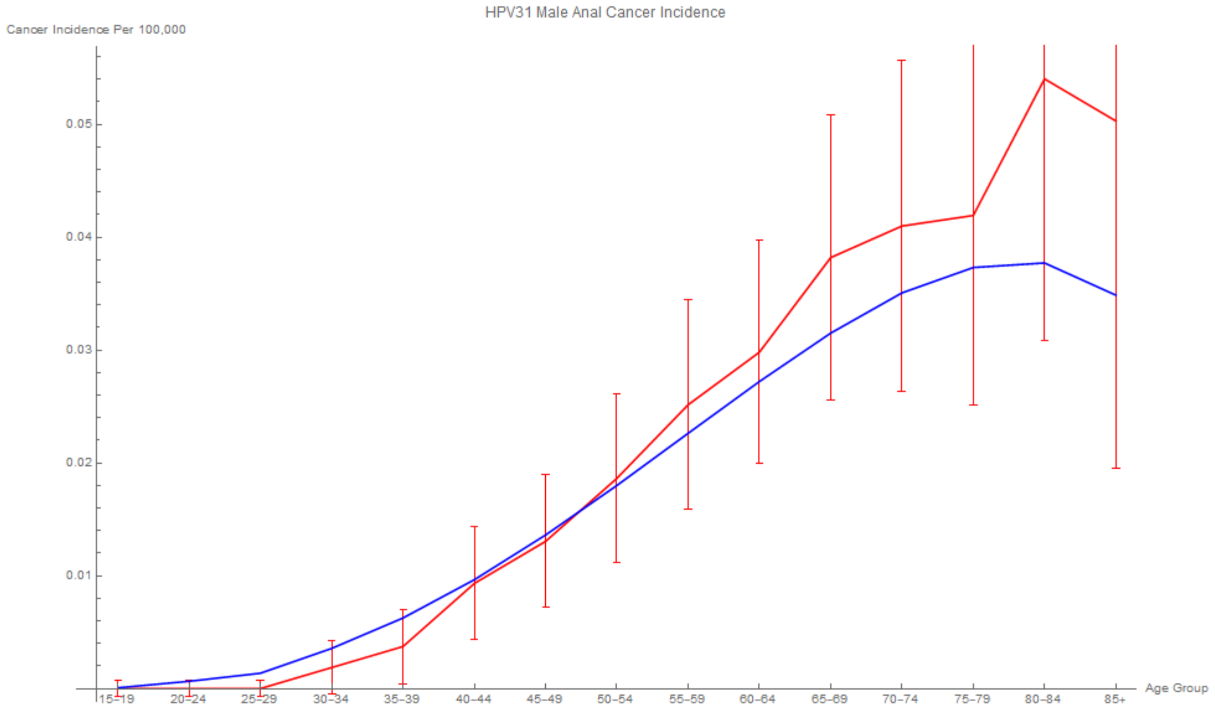

Figure 4. HPV 31 male anal cancer incidence

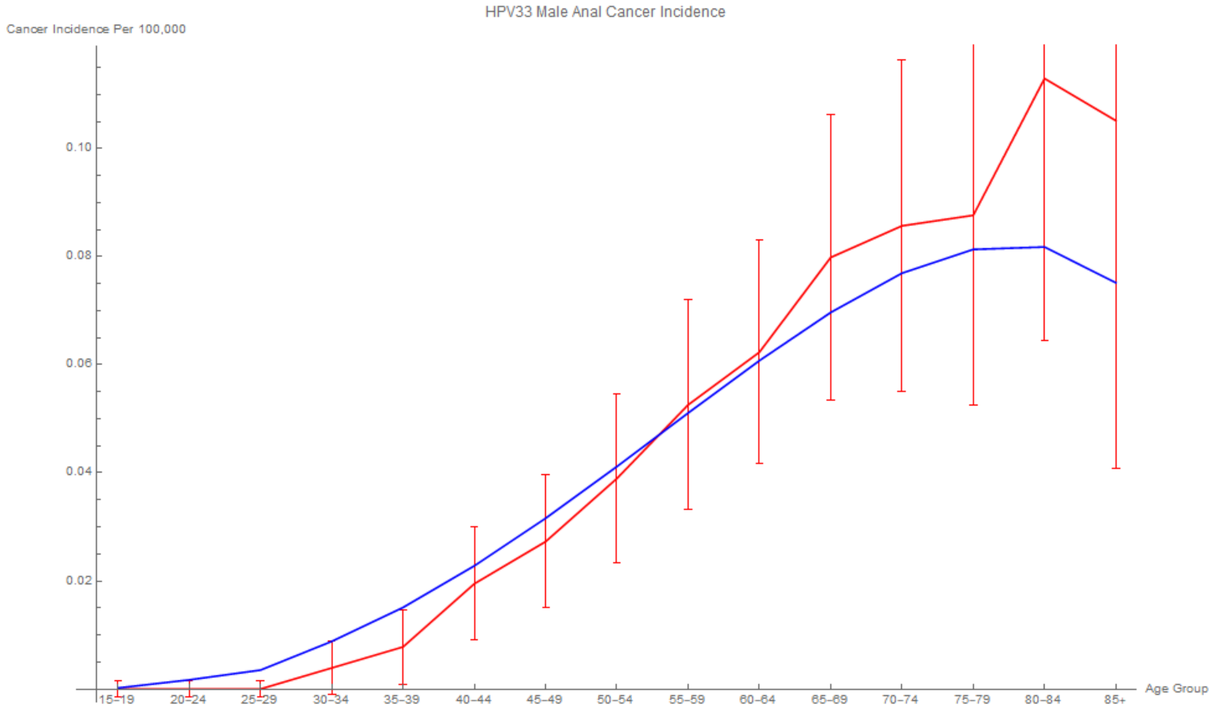

Figure 5. HPV 33 male anal cancer incidence

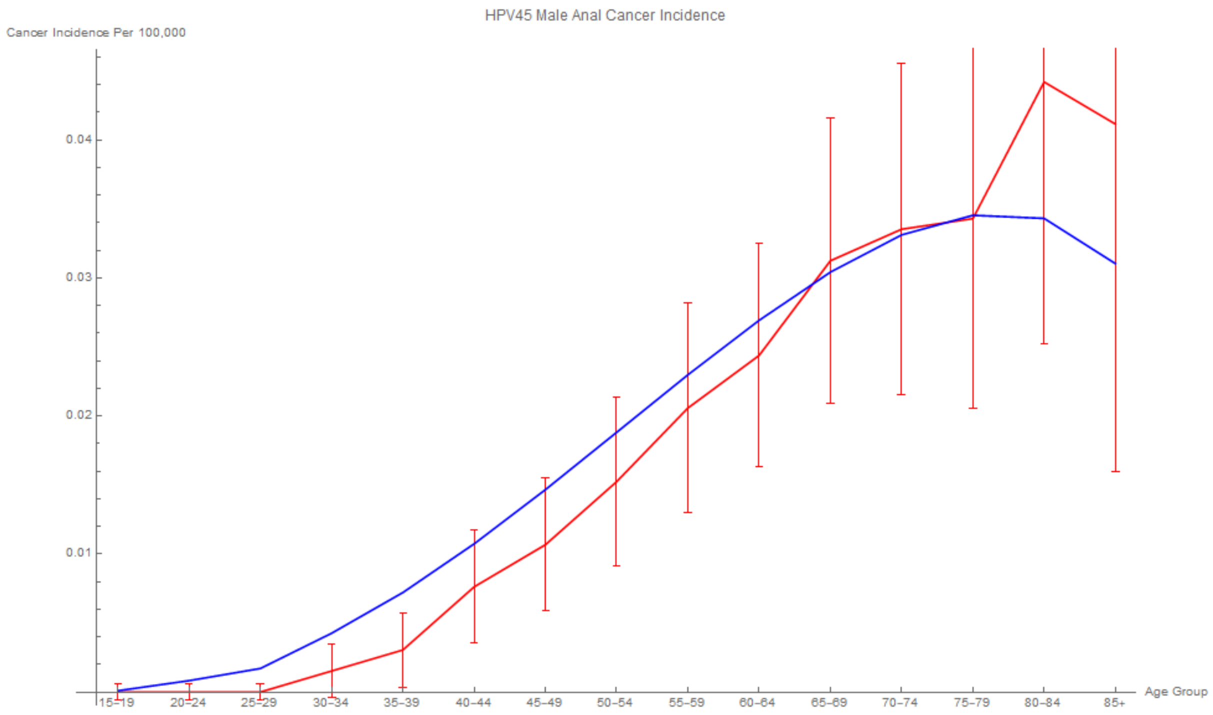

Figure 6. HPV 45 male anal cancer incidence

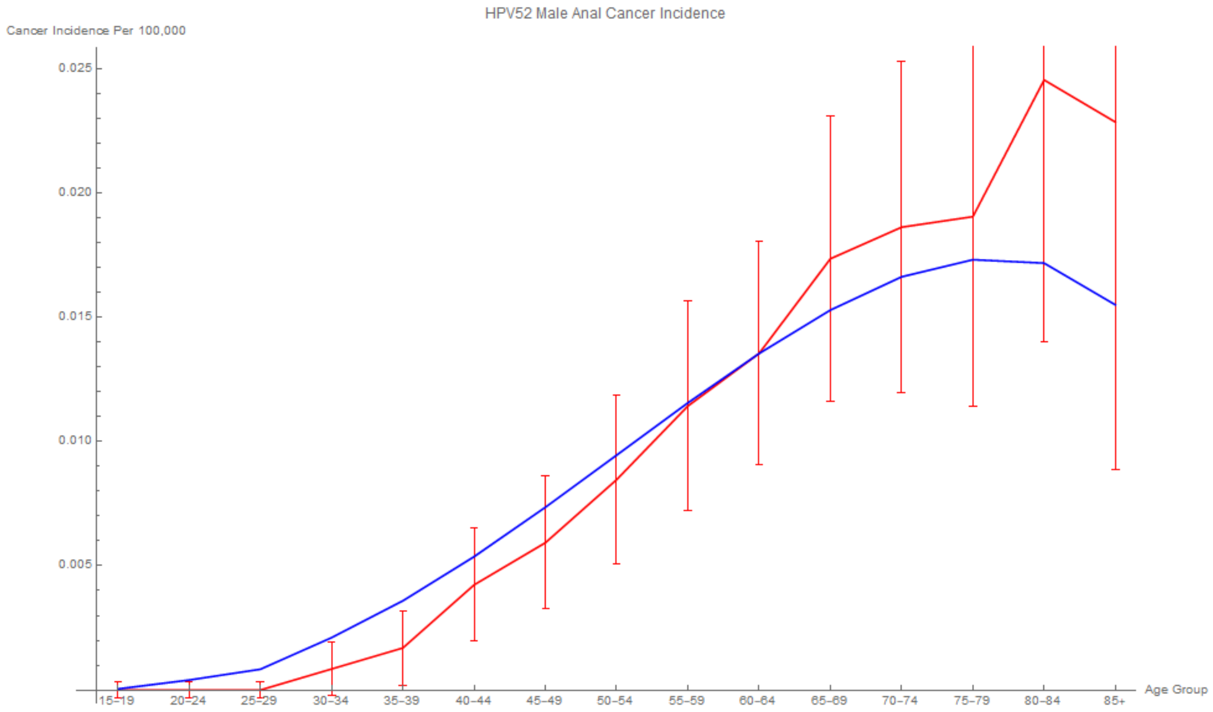

Figure 7. HPV 52 male anal cancer incidence

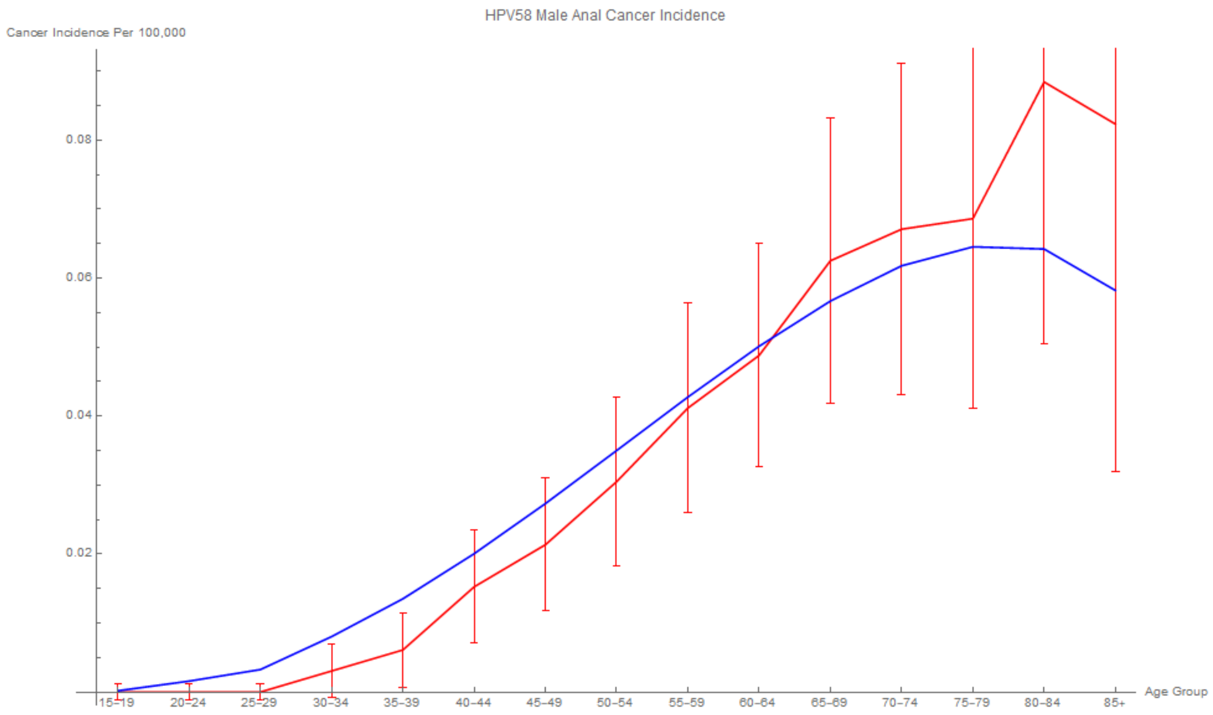

Figure 8. HPV 58 male anal cancer incidence

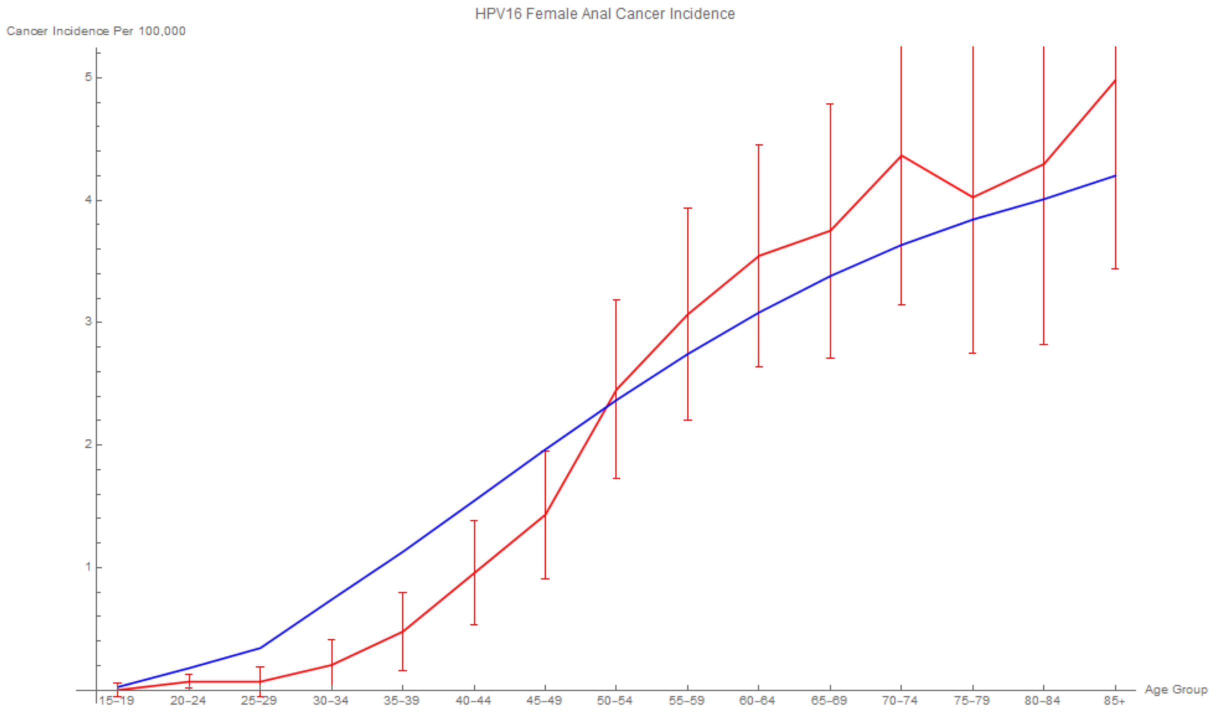

Figure 9. HPV 16 female anal cancer incidence

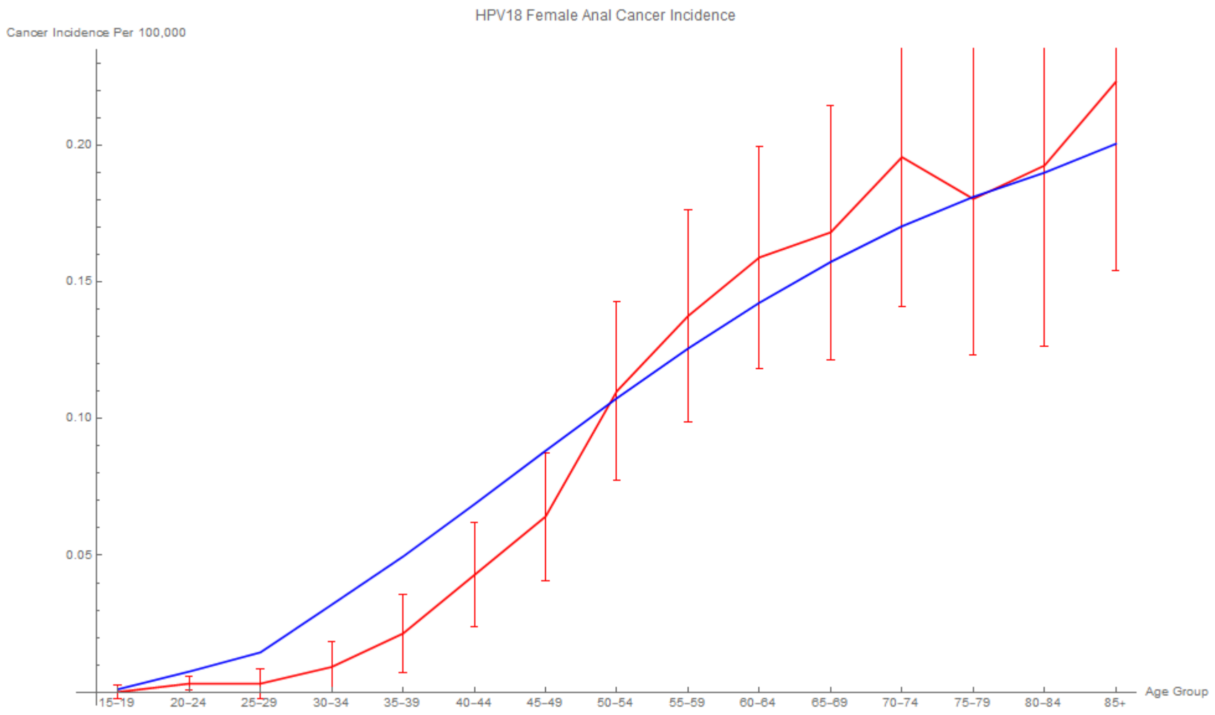

Figure 10. HPV 18 female anal cancer incidence

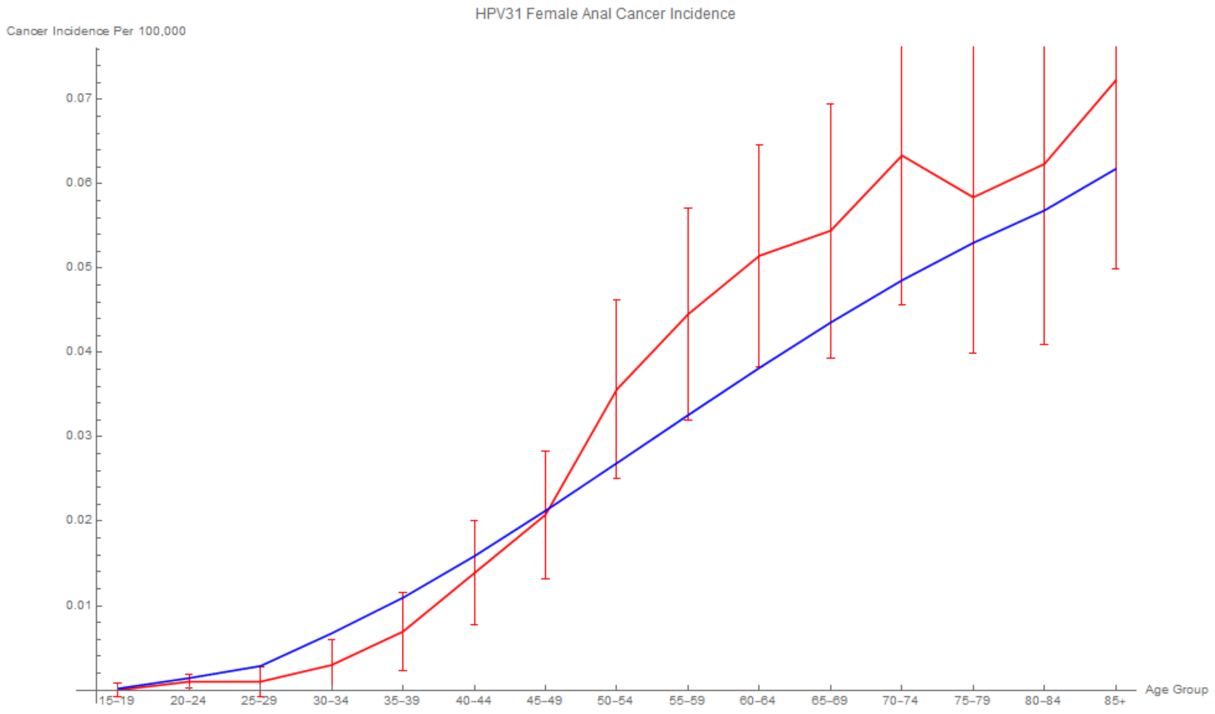

Figure 11. HPV 31 female anal cancer incidence

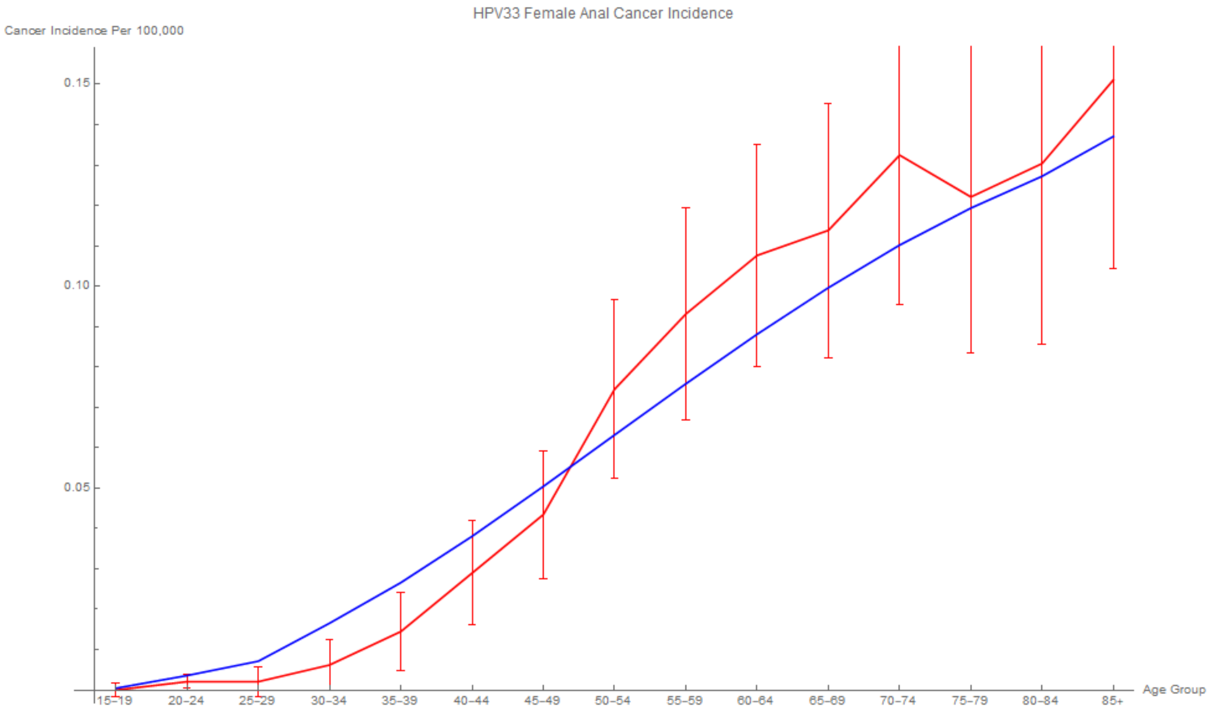

Figure 12. HPV 33 female anal cancer incidence

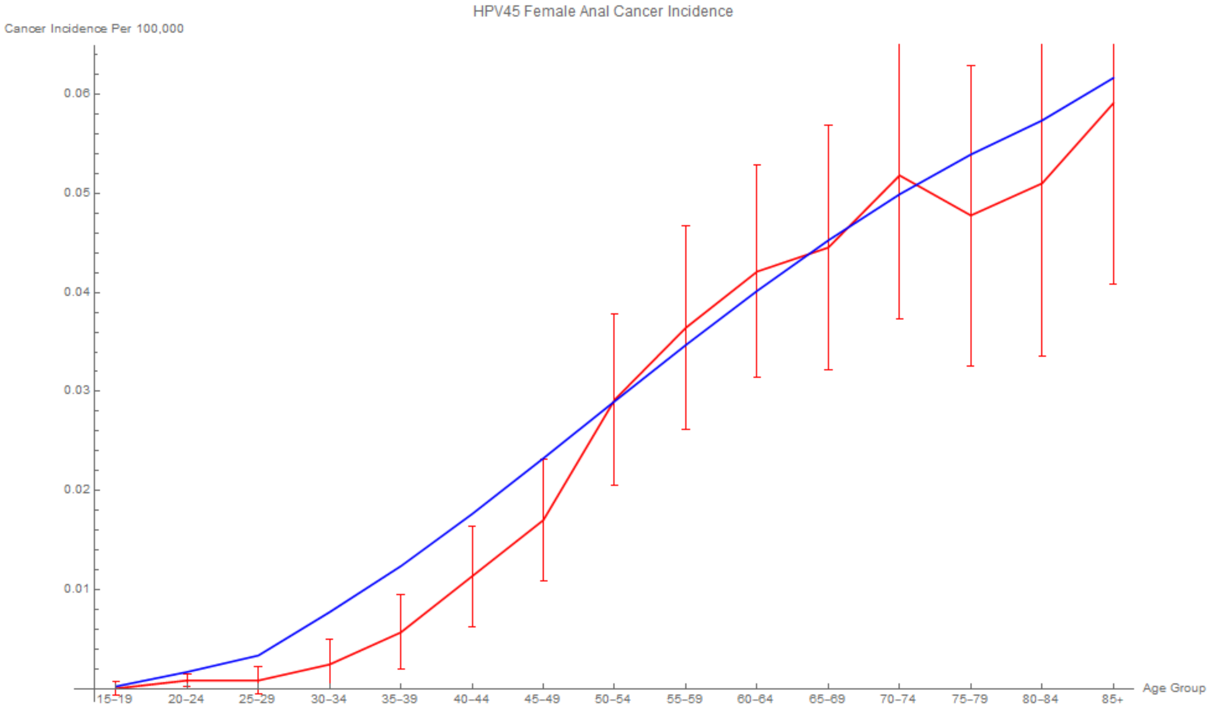

Figure 13. HPV 45 female anal cancer incidence

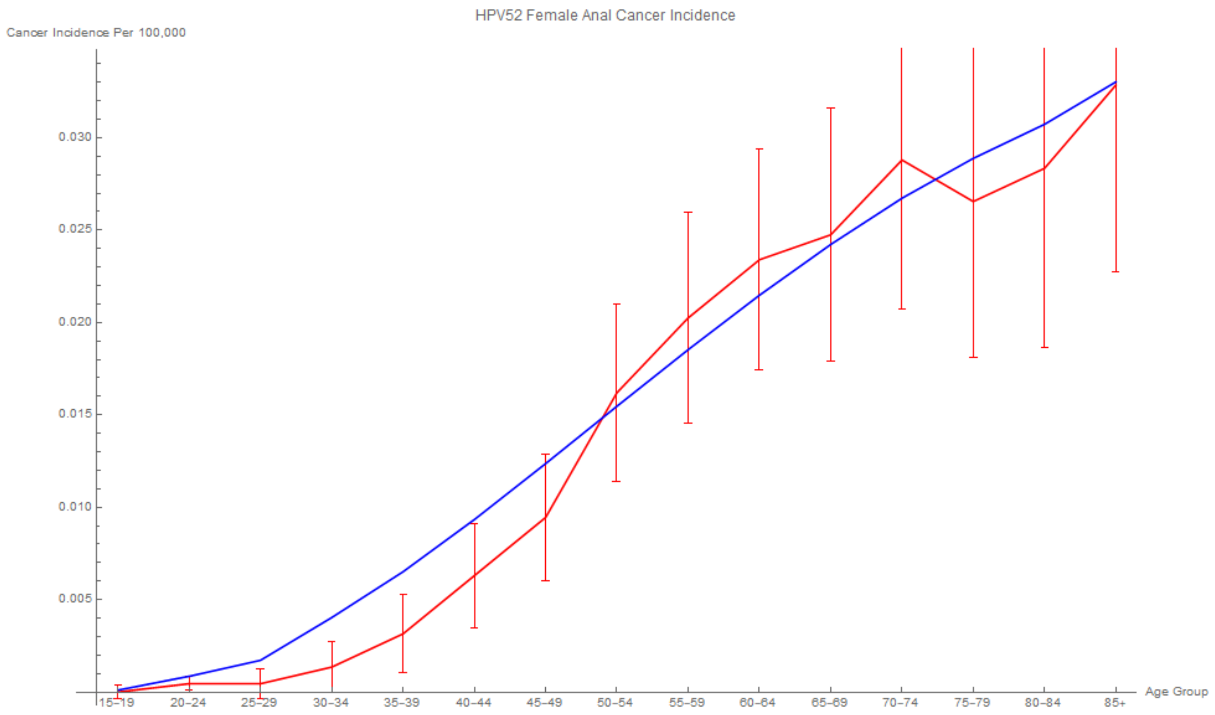

Figure 14. HPV 52 female anal cancer incidence

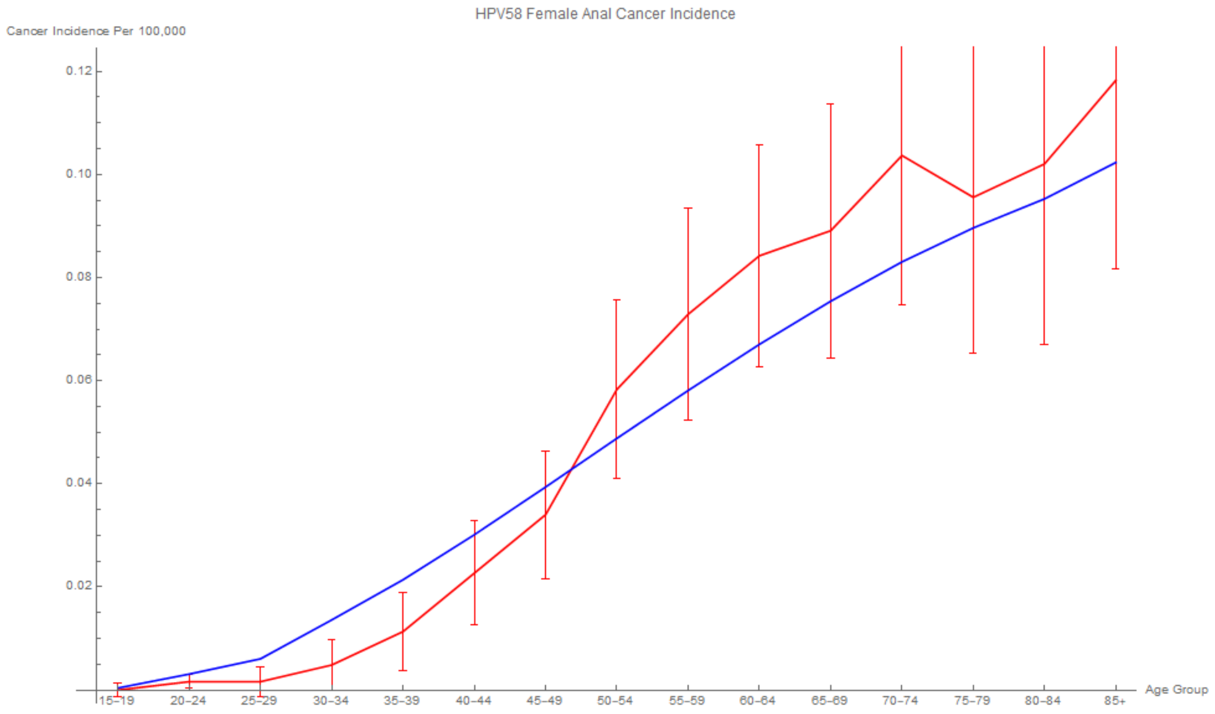

Figure 15. HPV 58 female anal cancer incidence

11.2 Cervical

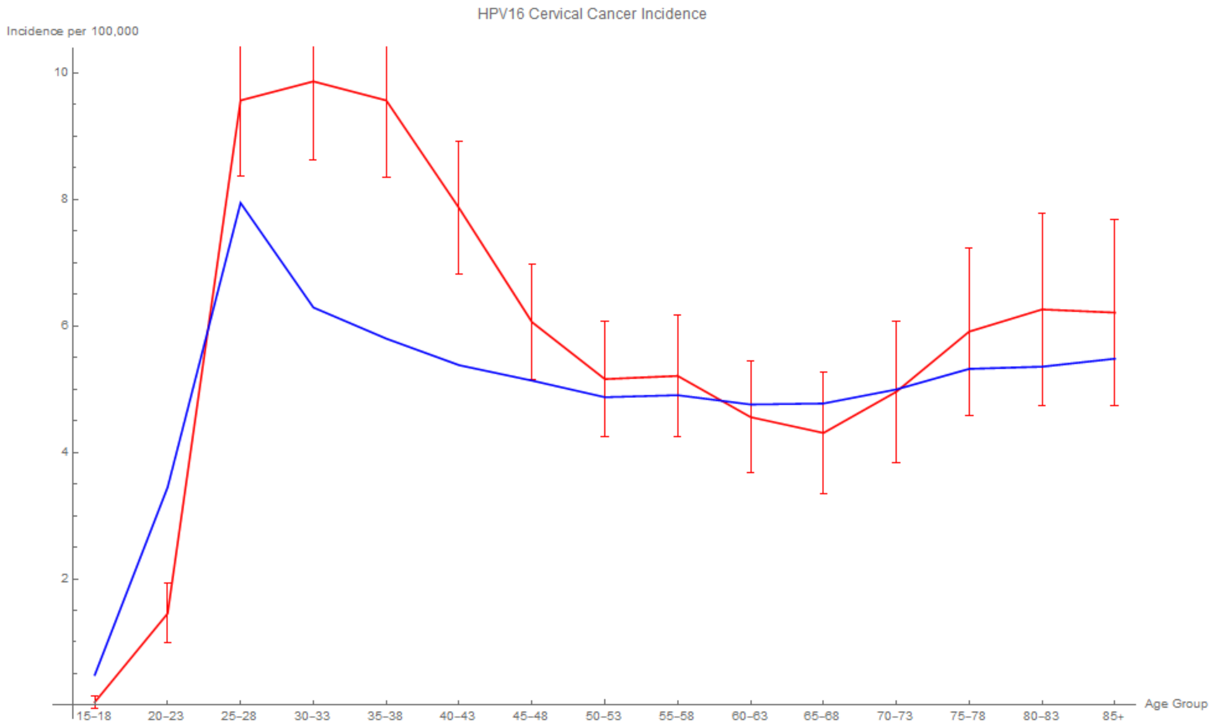

Figure 16. HPV 16 cervical cancer incidence

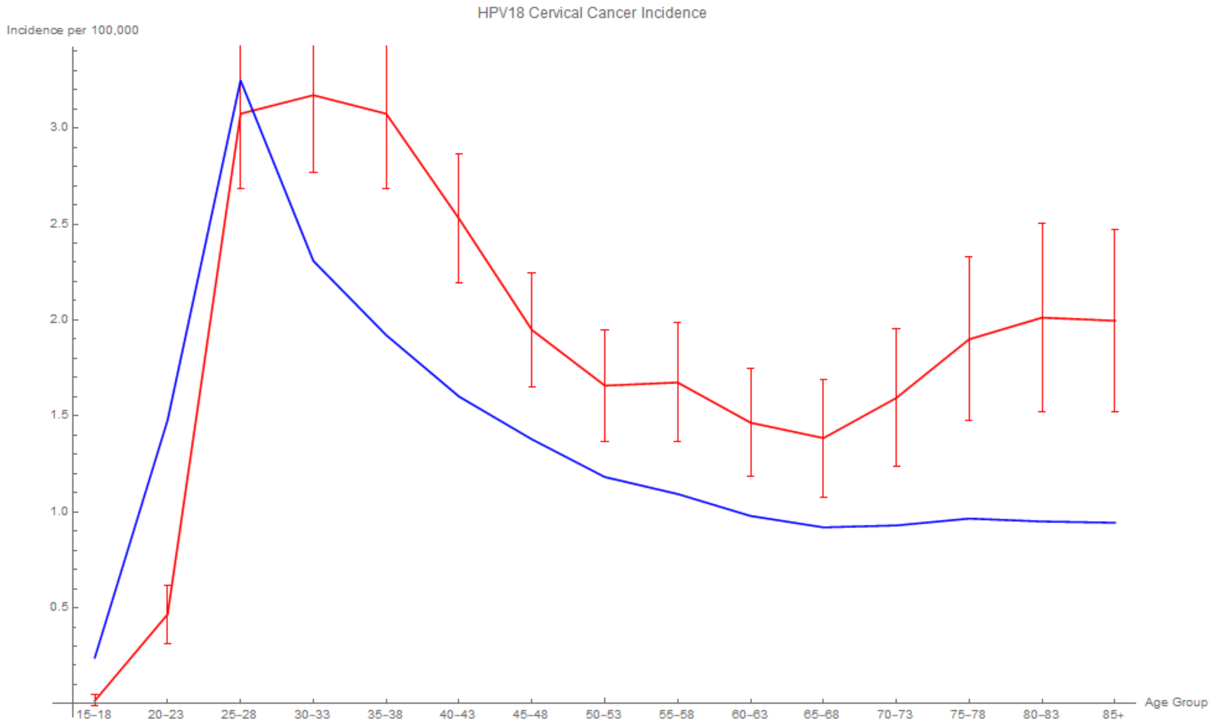

Figure 17. HPV 18 cervical cancer incidence

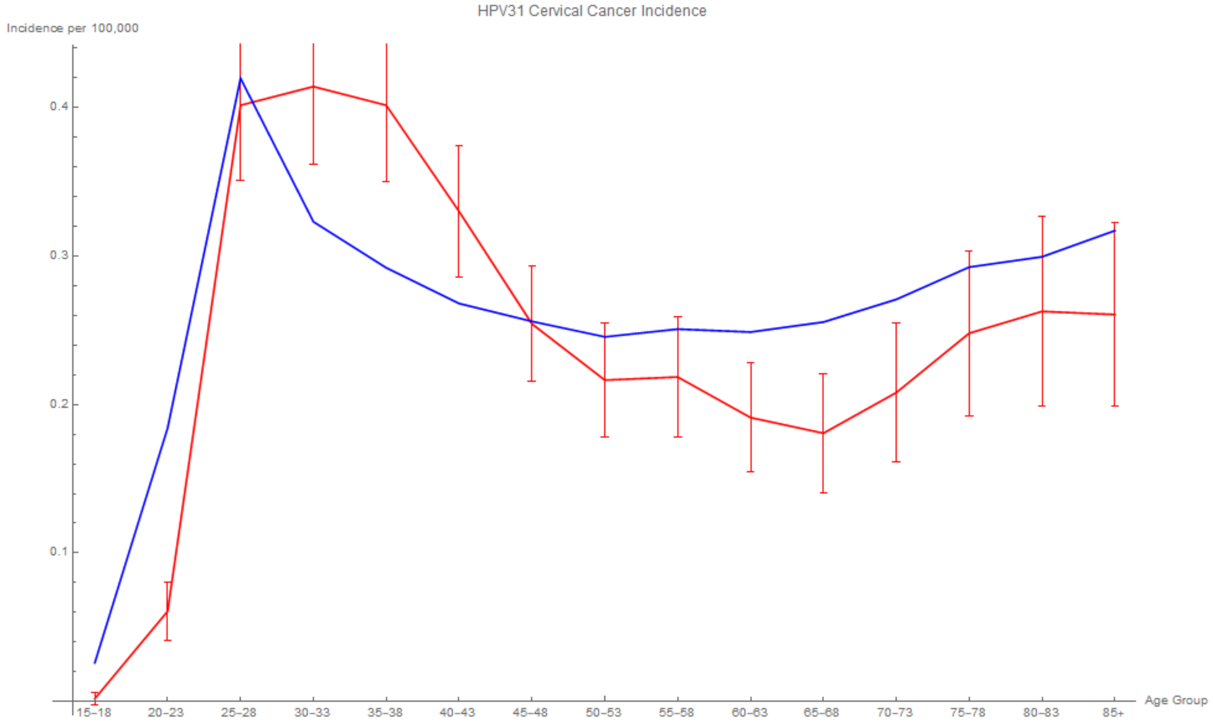

Figure 18. HPV 31 cervical cancer incidence

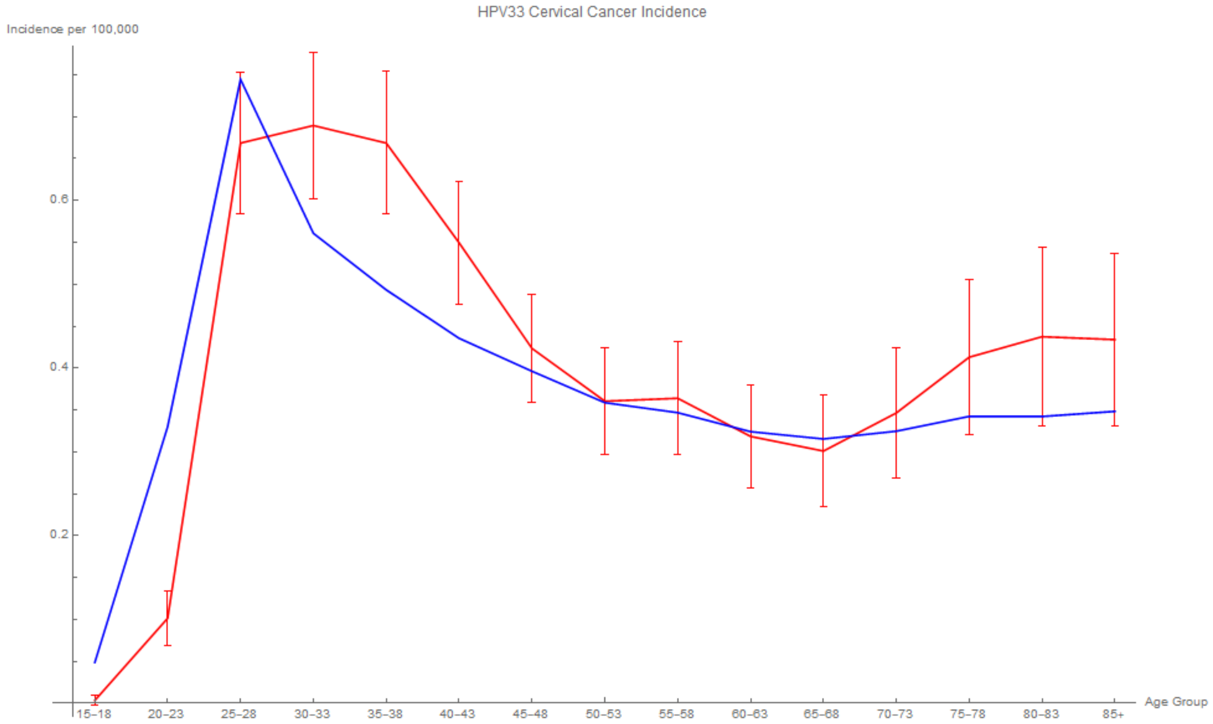

Figure 19 HPV 33 cervical cancer incidence

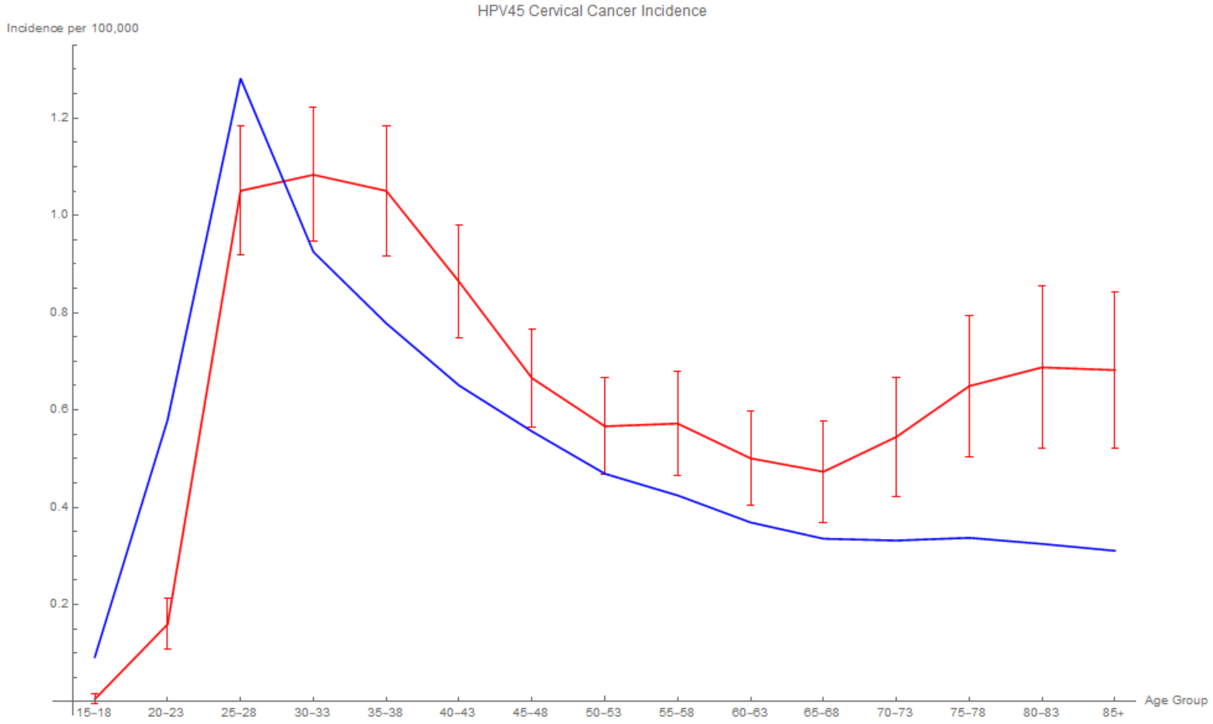

Figure 20. HPV 45 cervical cancer incidence

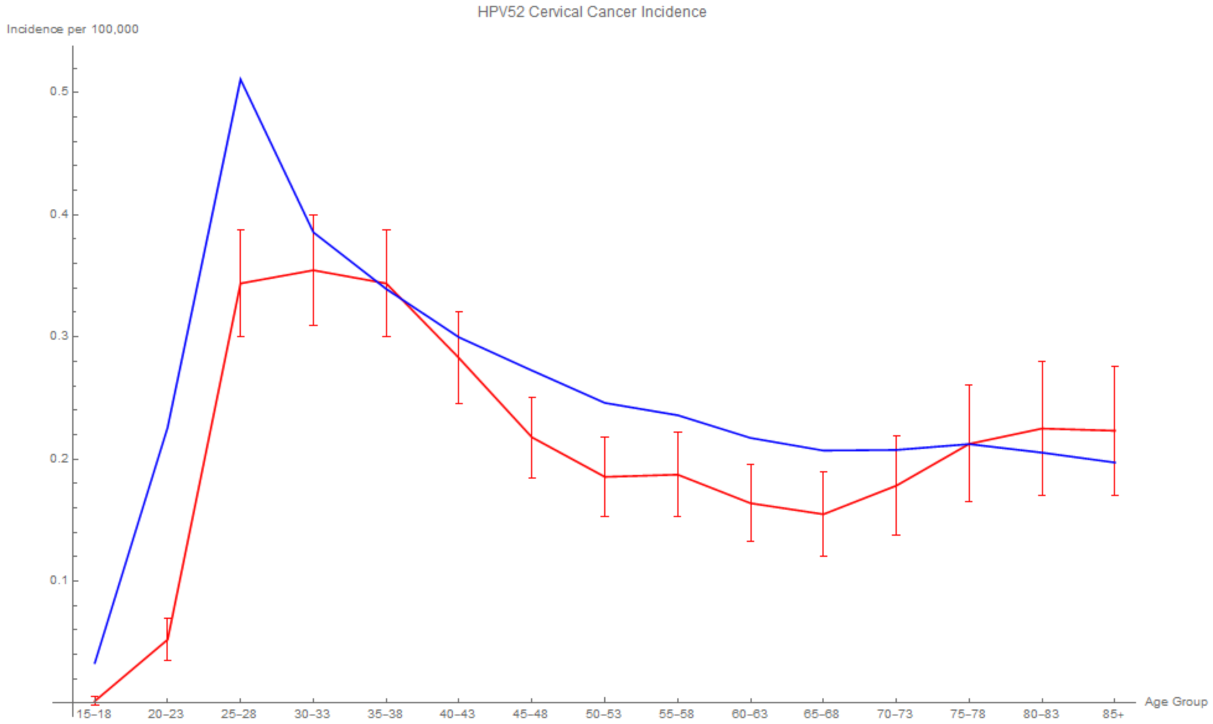

Figure 21. HPV 52 cervical cancer incidence

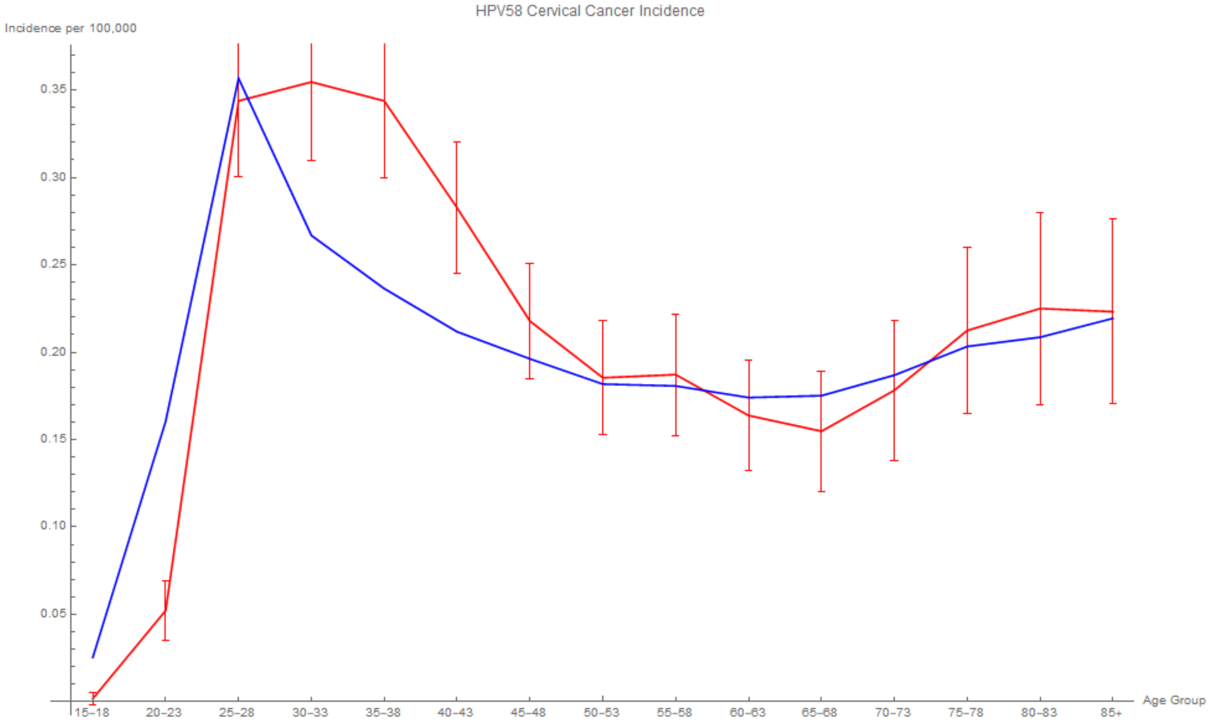

Figure 22. HPV 58 cervical cancer incidence

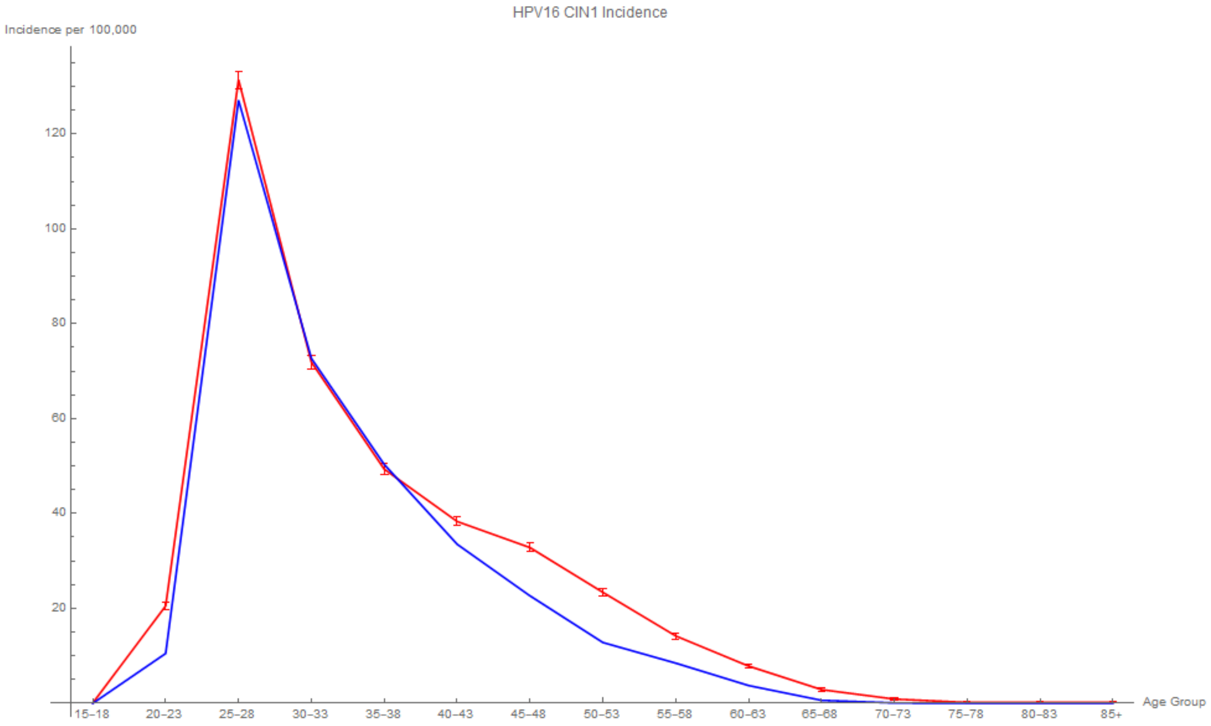

Figure 23. HPV 16 CIN1 incidence

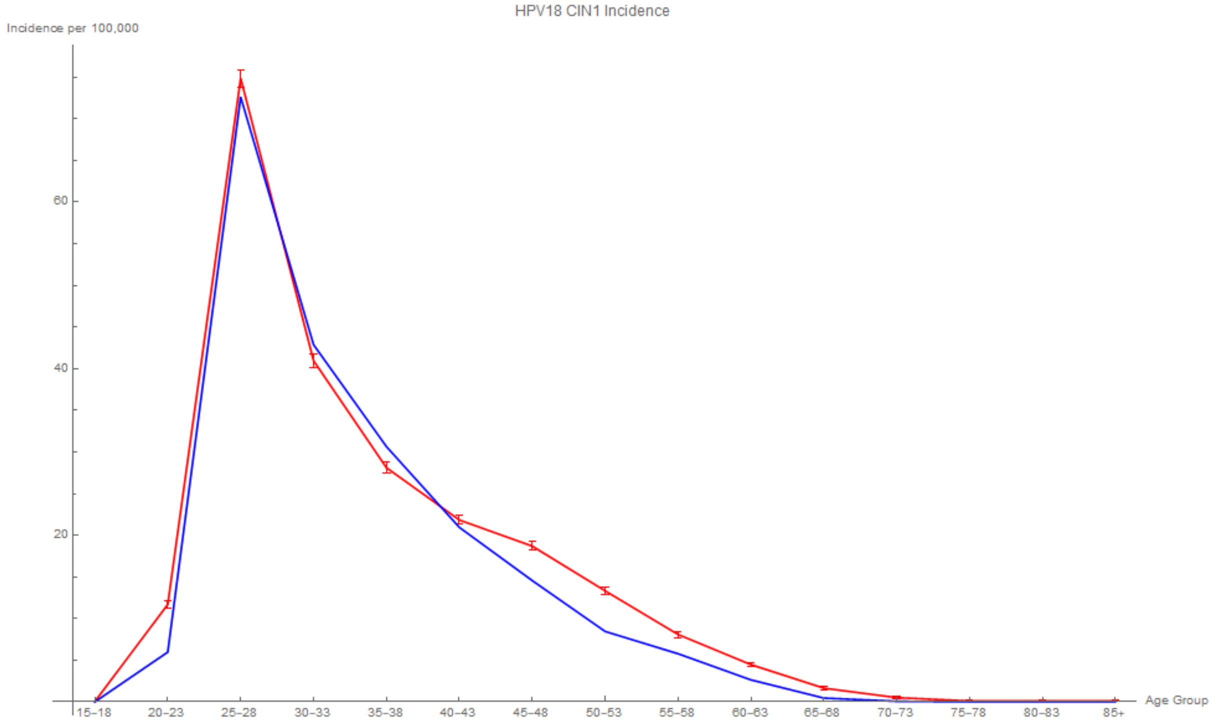

Figure 24. HPV 18 CIN1 incidence

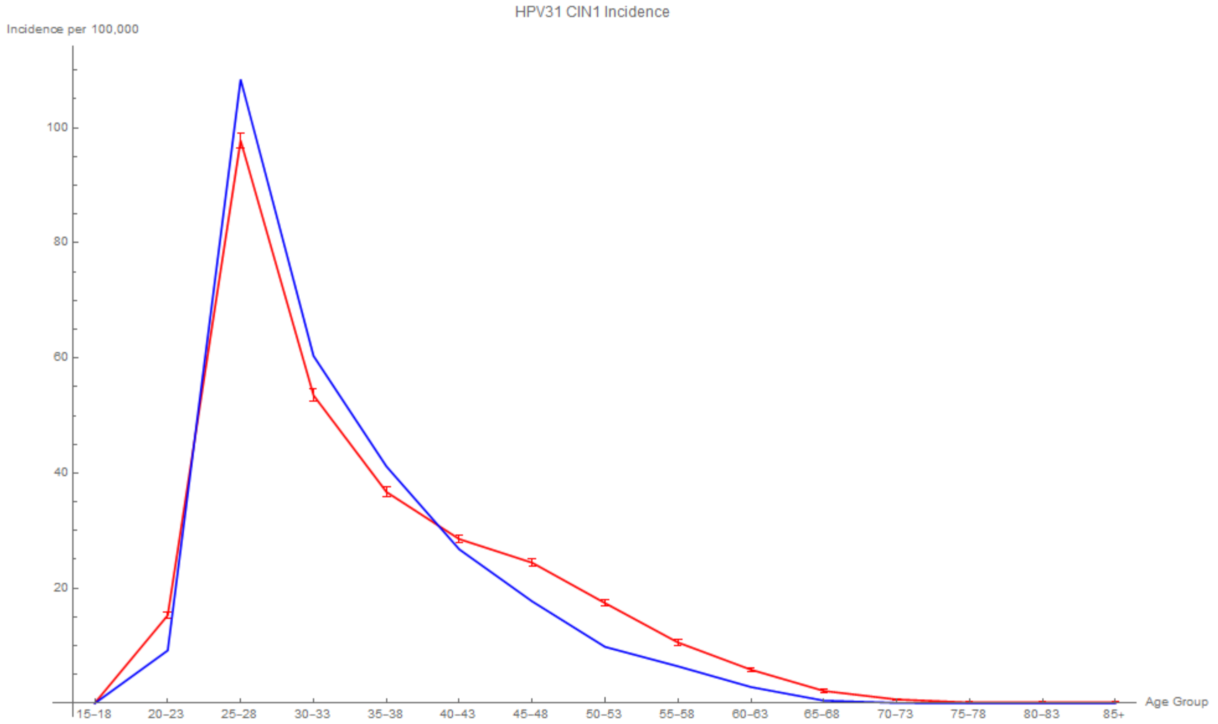

Figure 25. HPV 31 CIN1 incidence

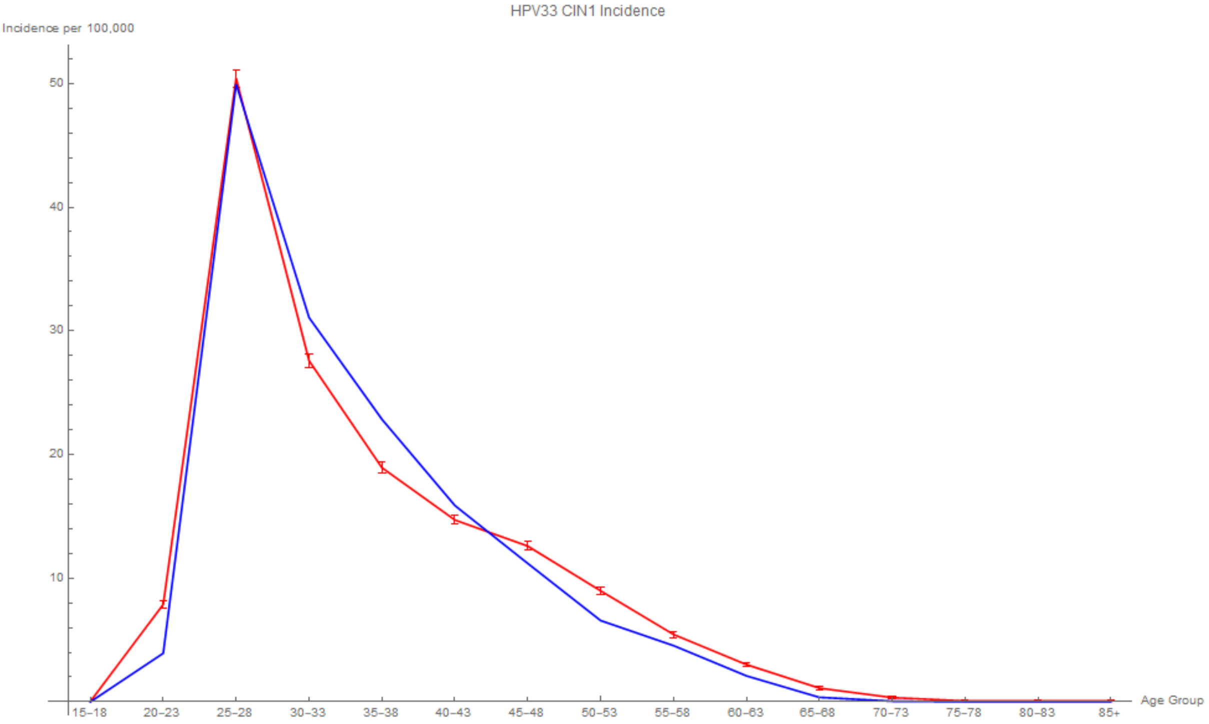

Figure 26. HPV 33 CIN1 incidence

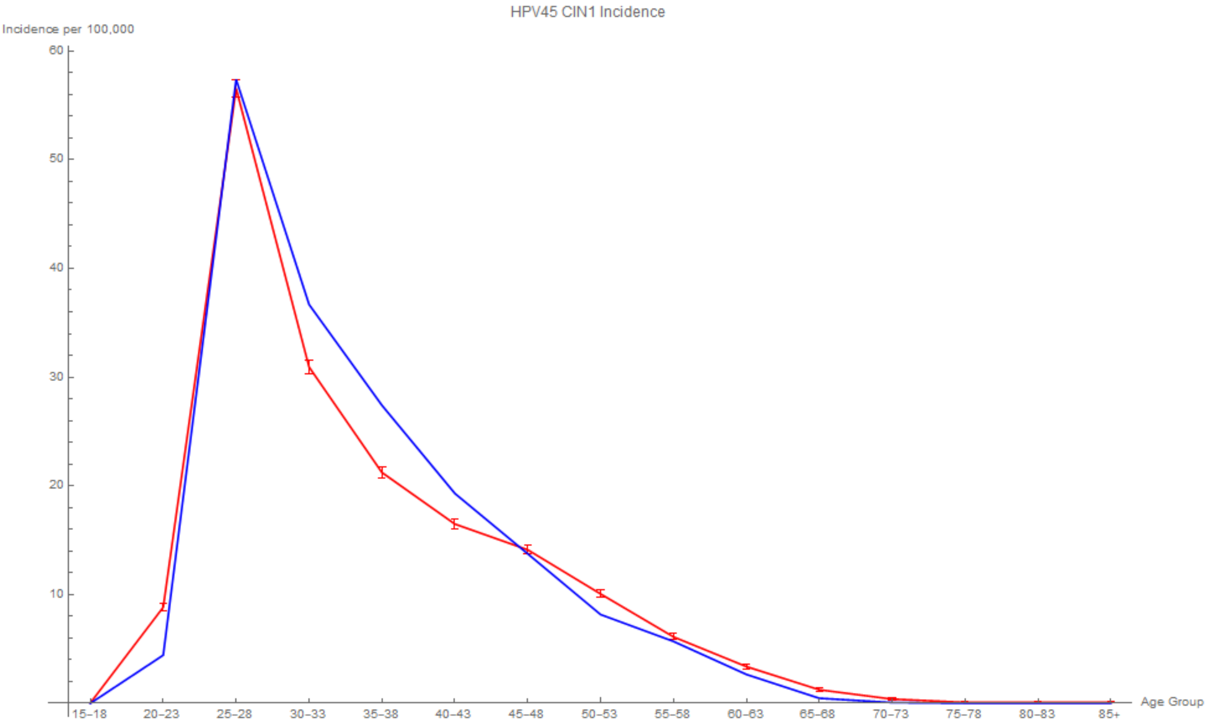

Figure 27. HPV 45 CIN1 incidence

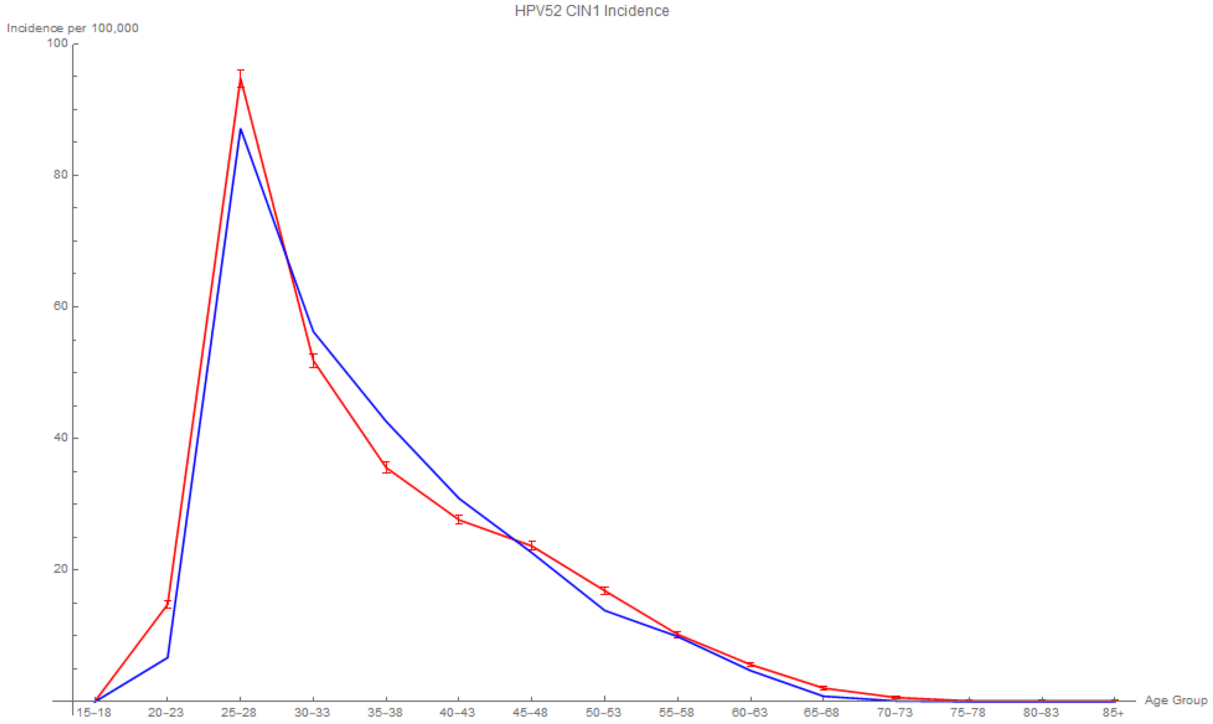

Figure 28. HPV 52 CIN1 incidence

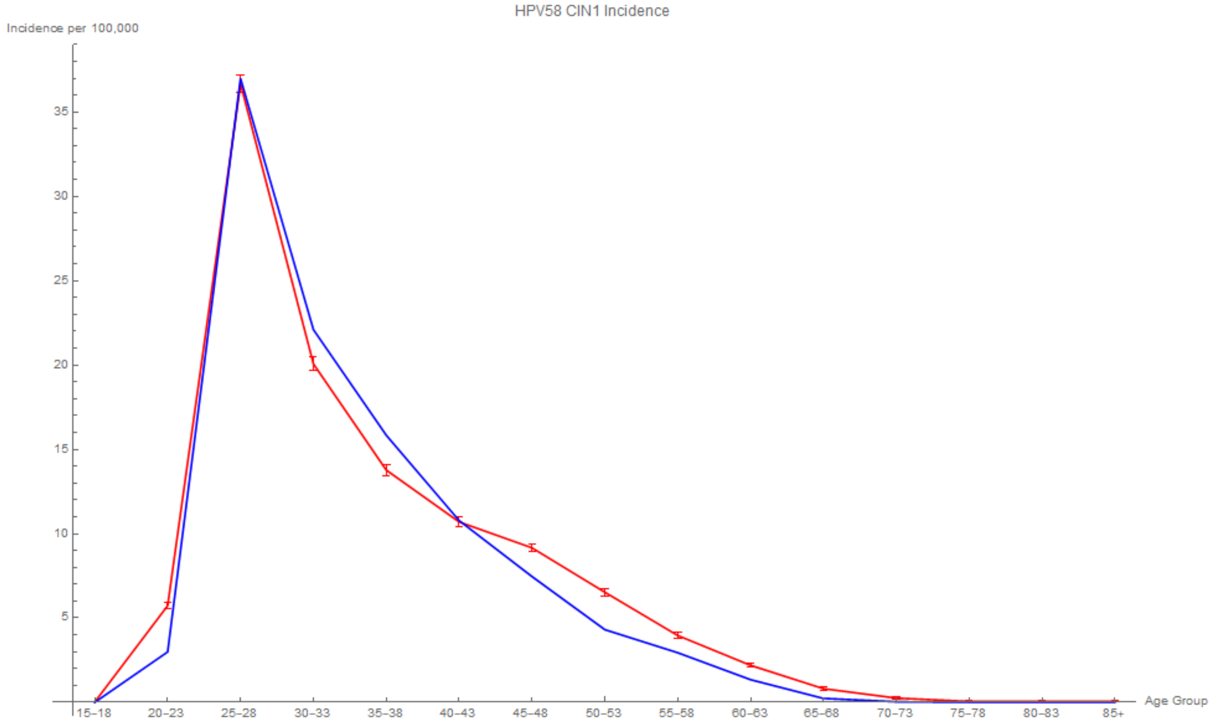

Figure 29. HPV 58 CIN1 incidence

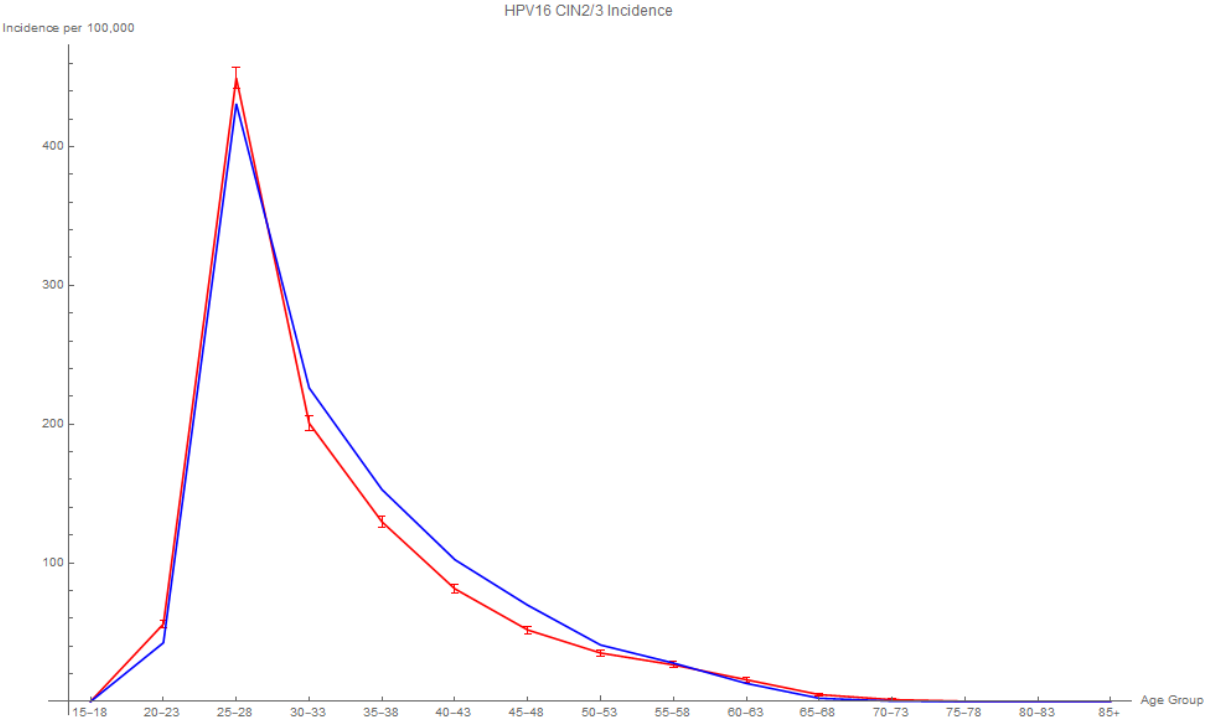

Figure 30. HPV 16 CIN2/3 incidence

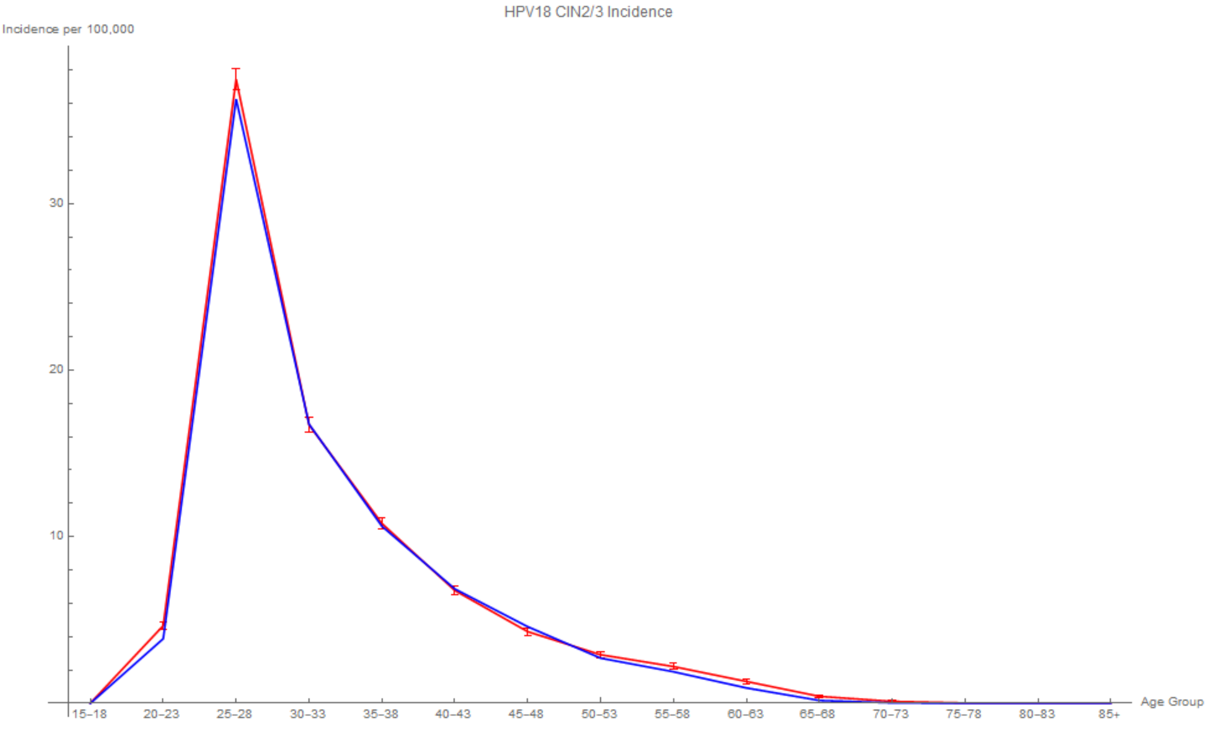

Figure 31. HPV 18 CIN2/3 incidence

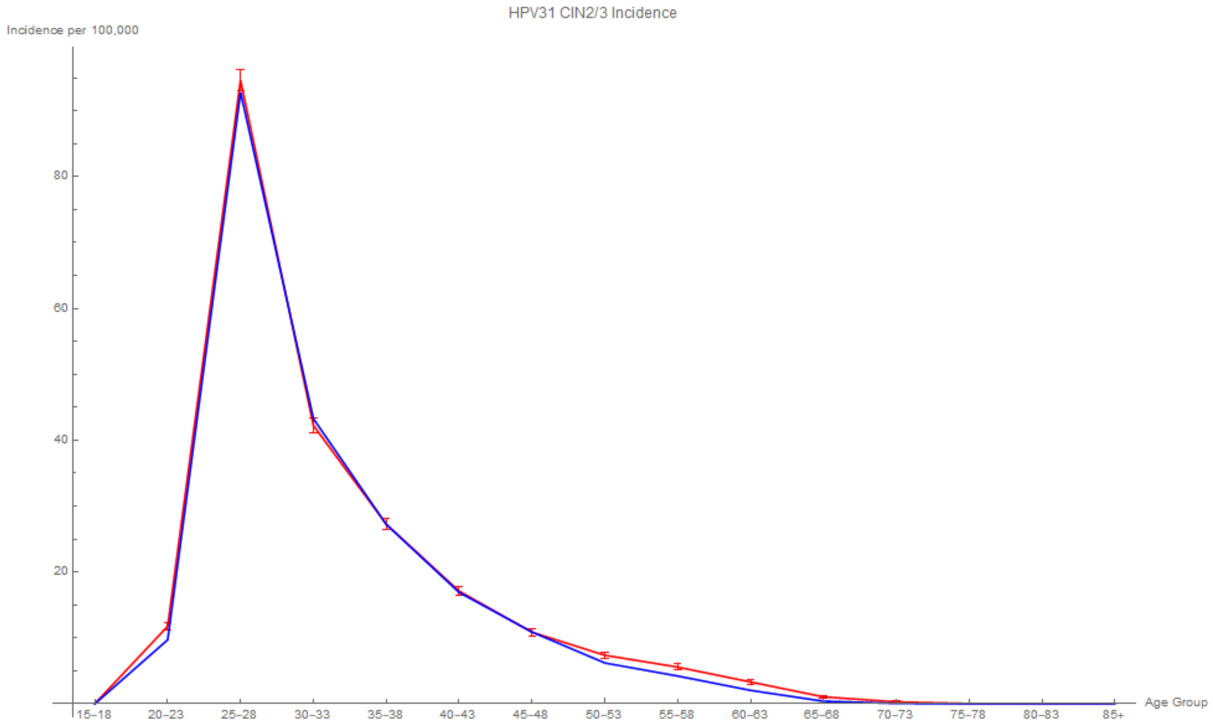

Figure 32. HPV 31 CIN2/3 incidence

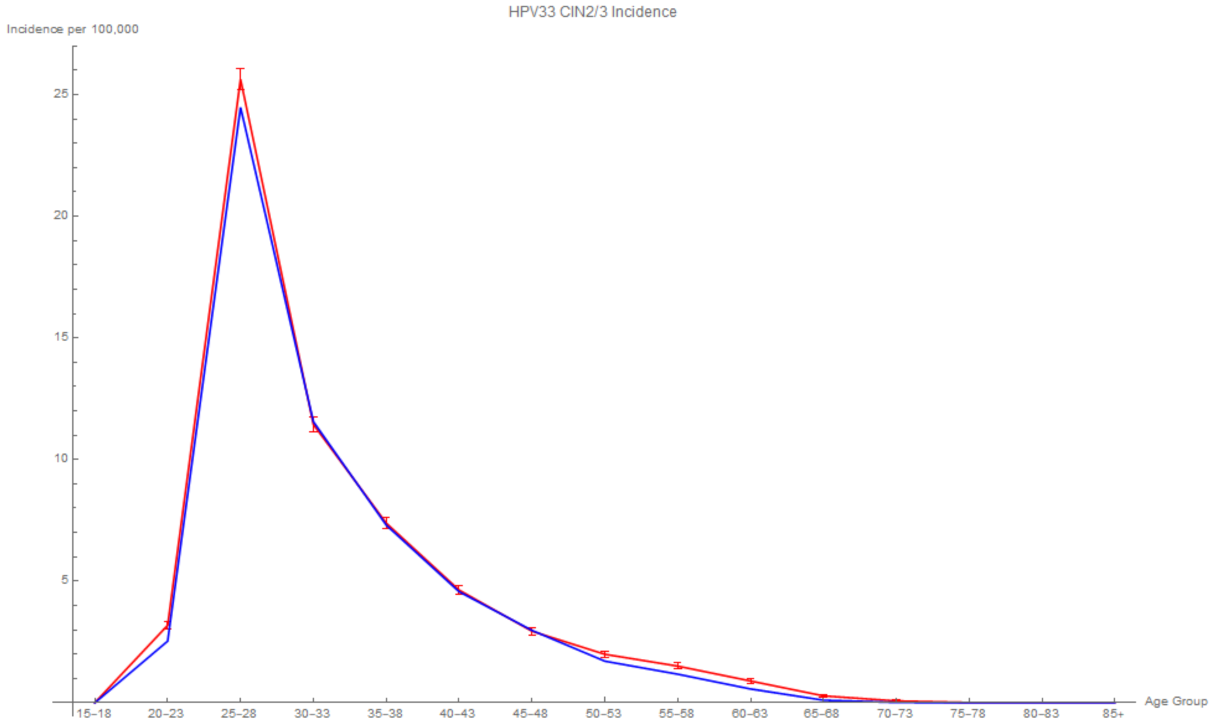

Figure 33. HPV 33 CIN2/3 incidence

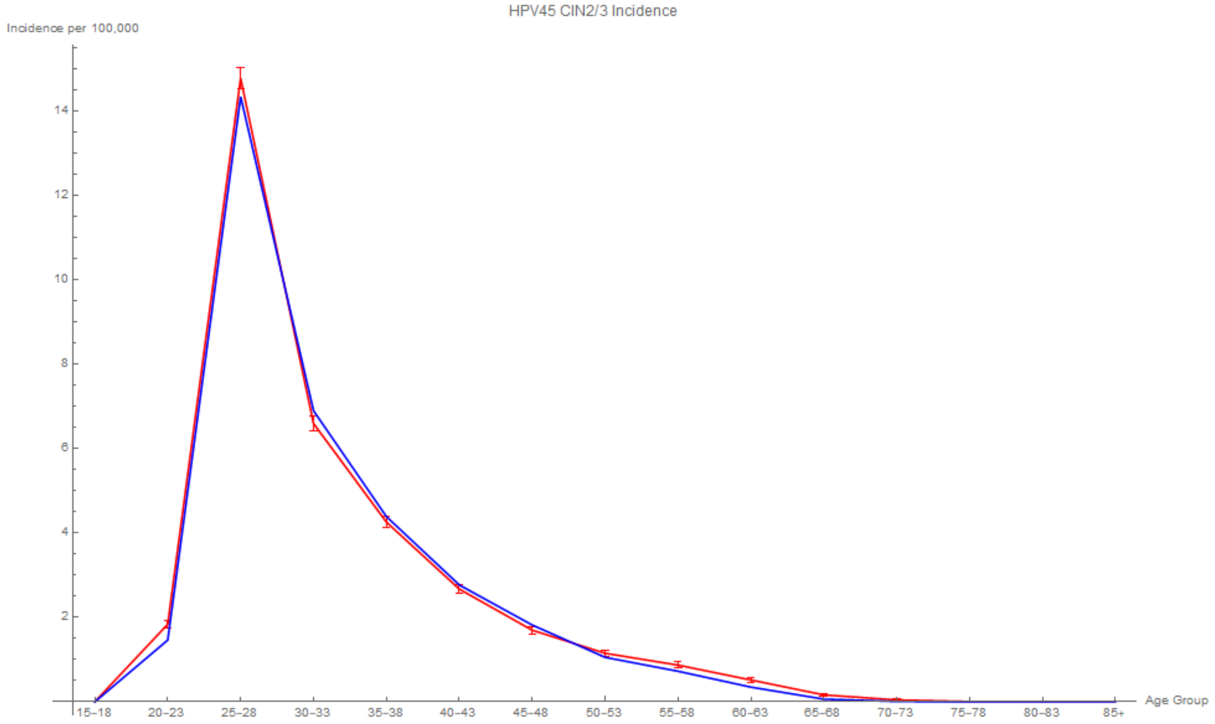

Figure 34. HPV 45 CIN2/3 incidence

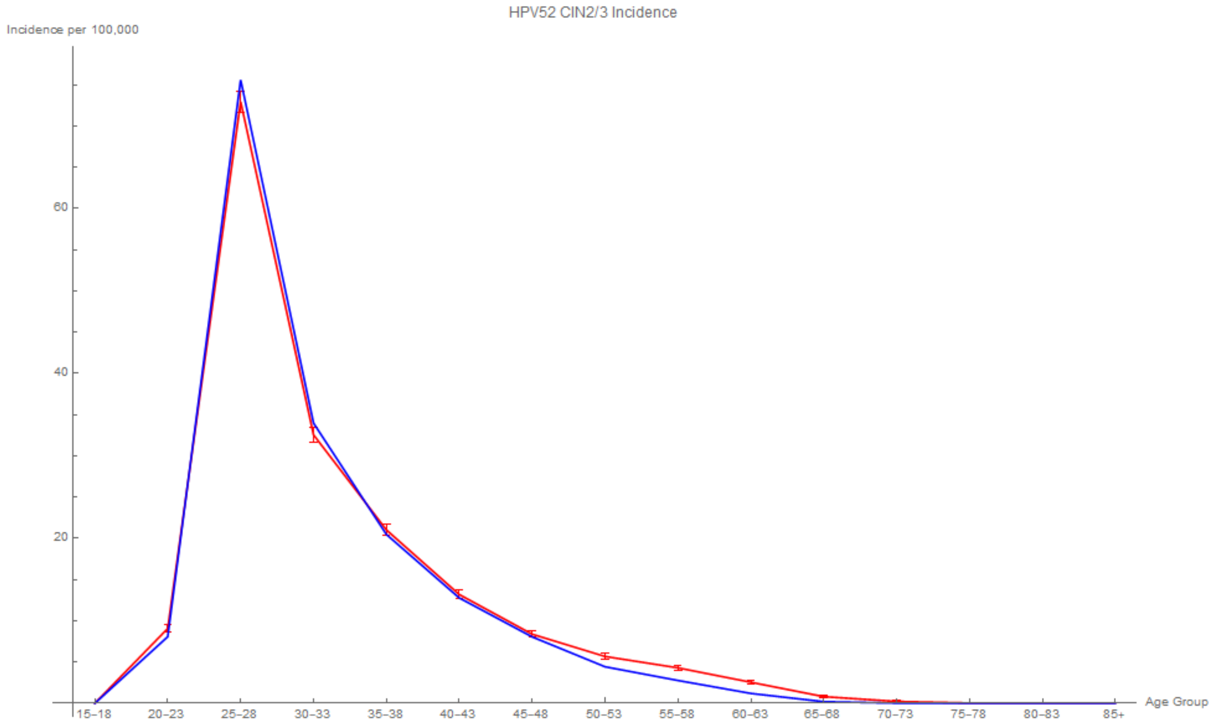

Figure 35. HPV 52 CIN2/3 incidence

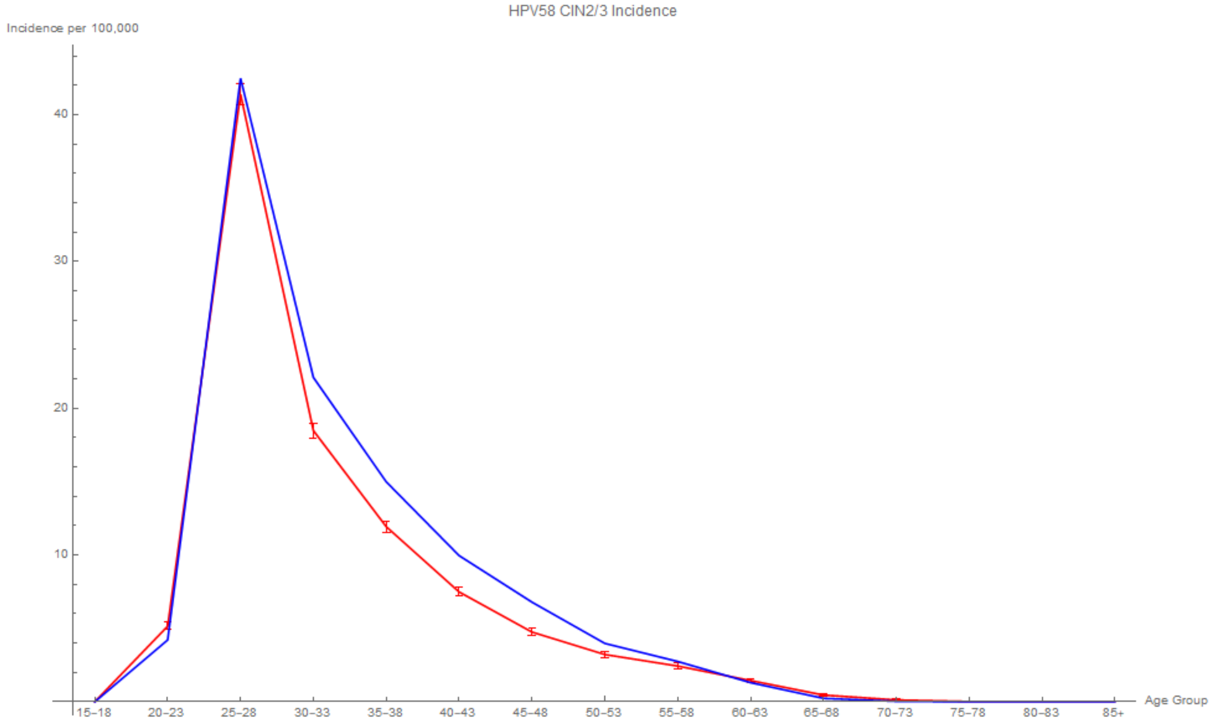

Figure 36. HPV 58 CIN2/3 incidence

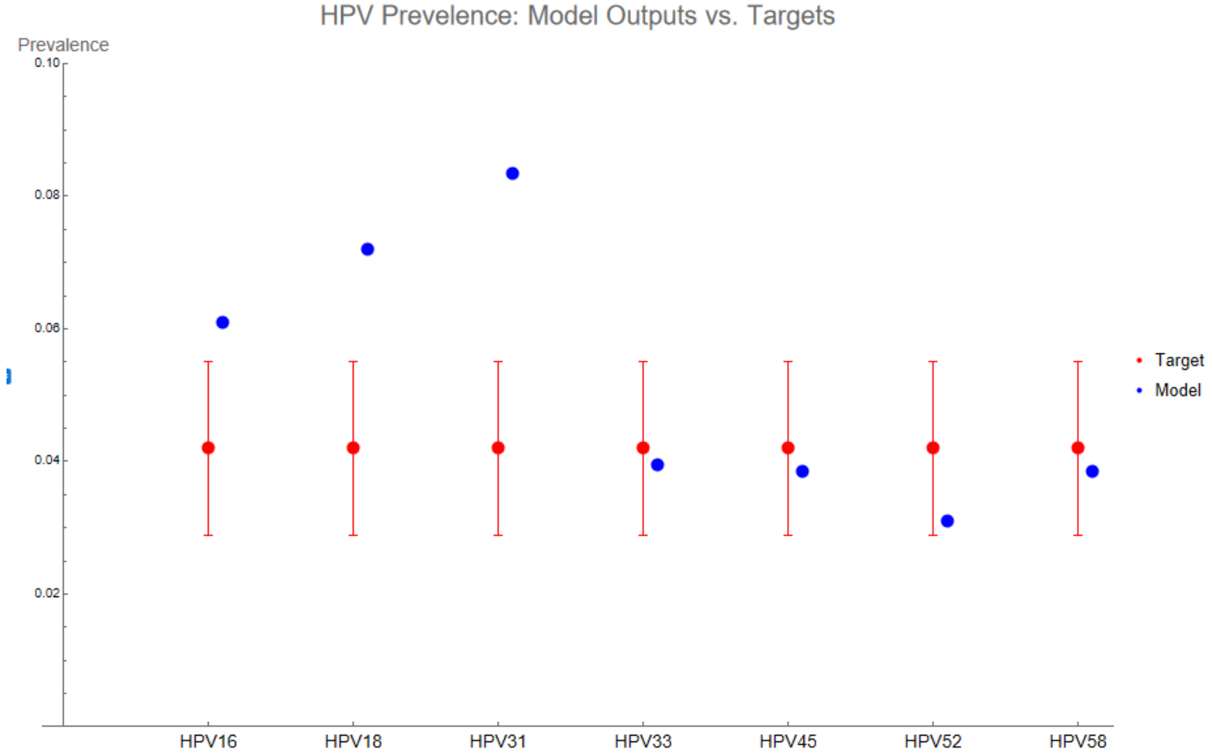

Figure 37. Cervical infection prevalence by HPV type

11.3 Genital Warts

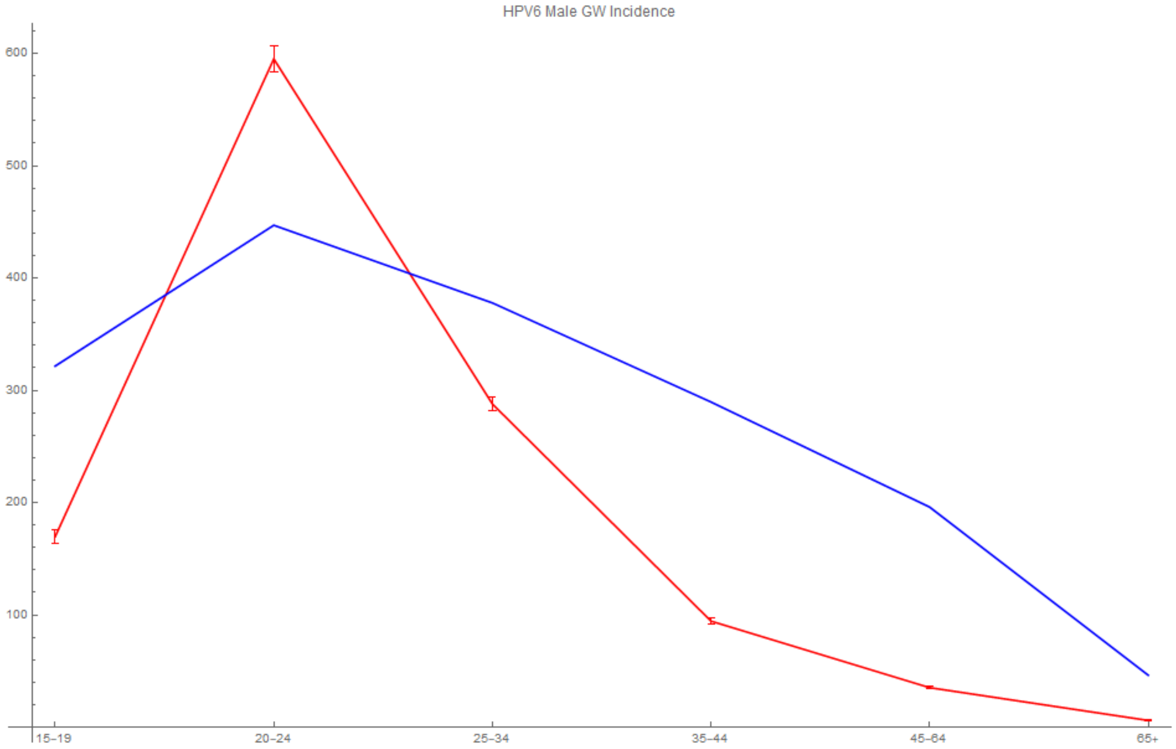

Figure 38. HPV 6 male genital warts incidence

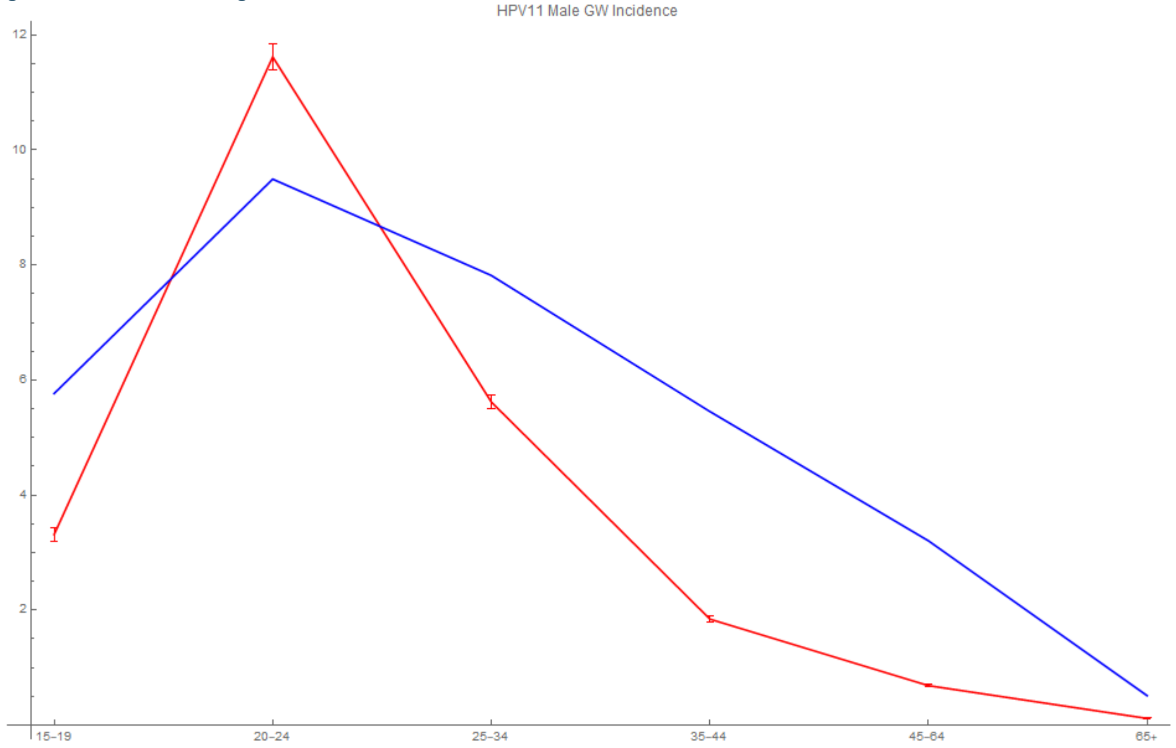

Figure 39. HPV 11 male genital warts incidence

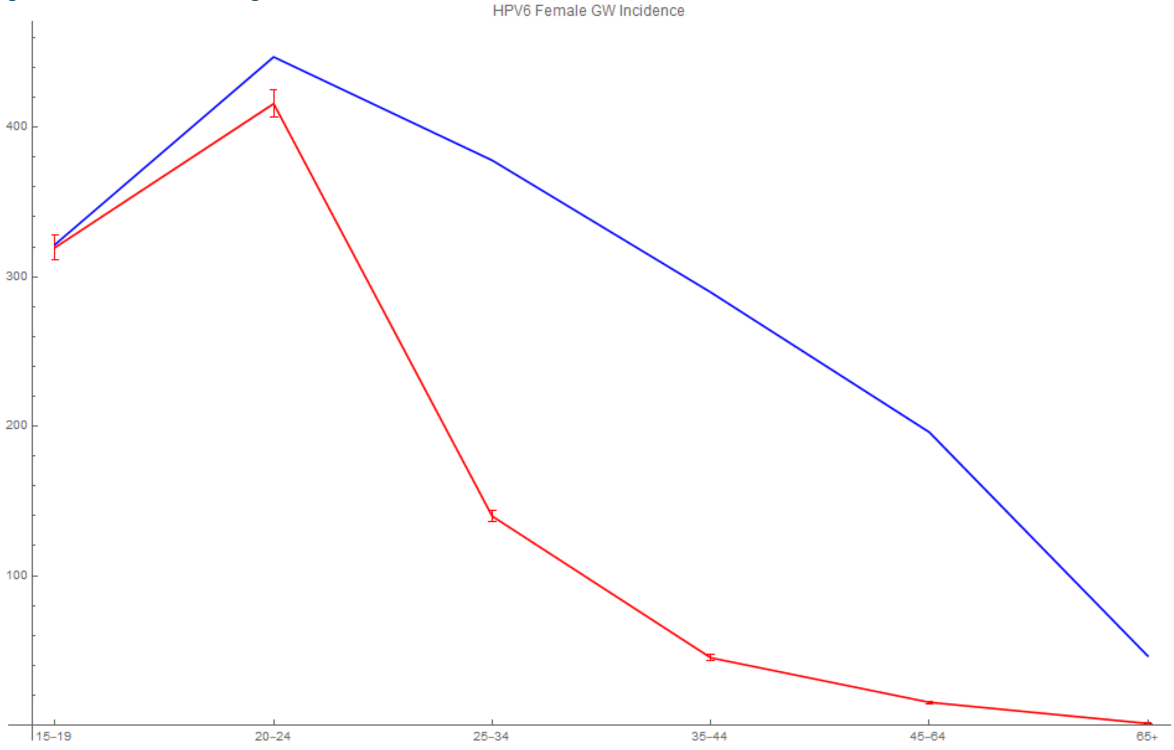

Figure 40. HPV 6 female genital warts incidence

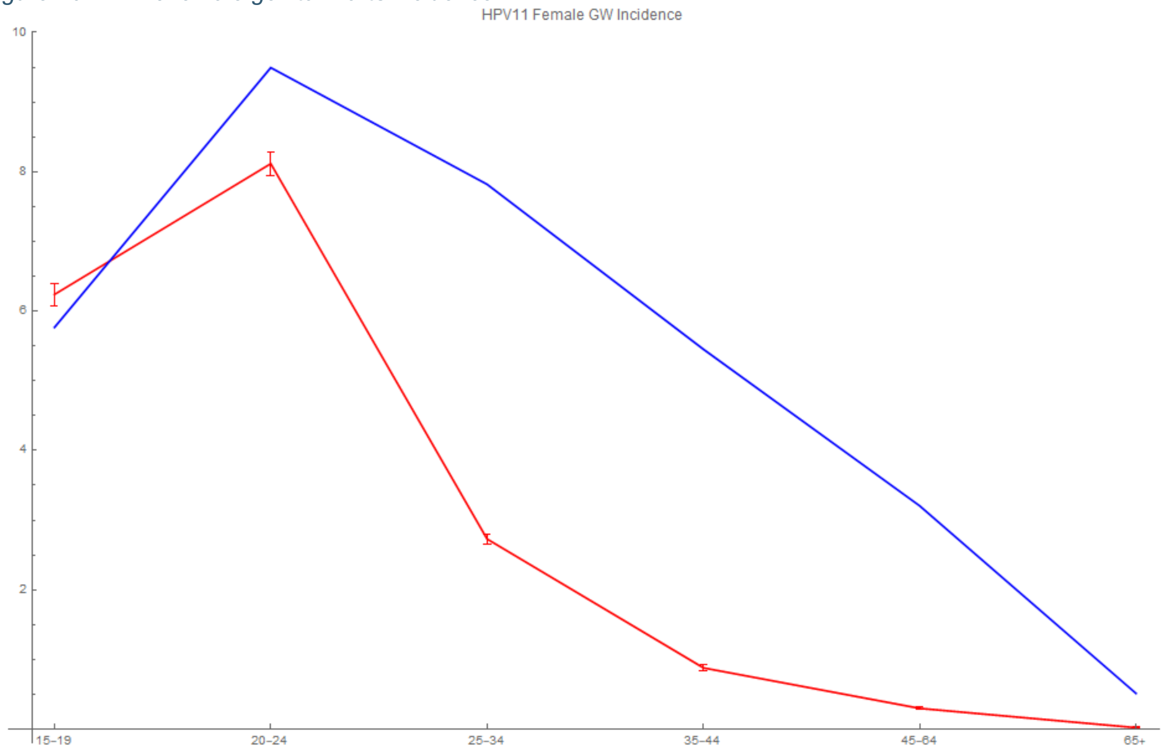

Figure 41. HPV 11 male genital warts incidence

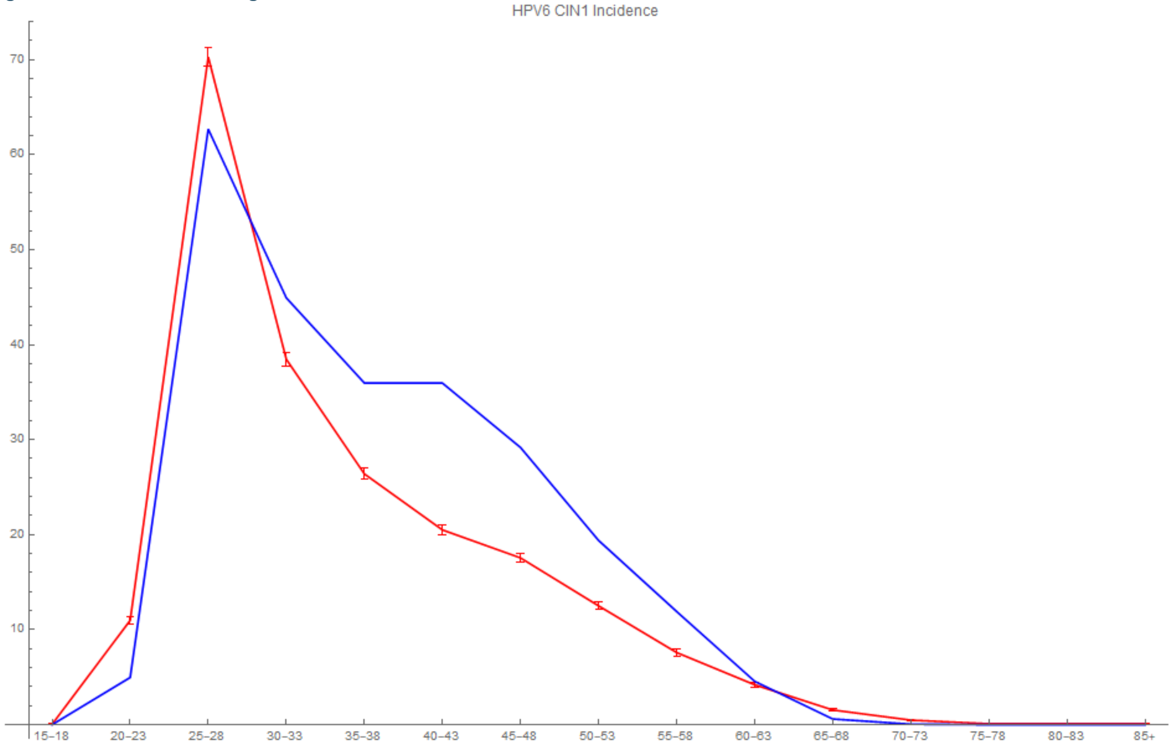

Figure 42. HPV 6 CIN1 incidence

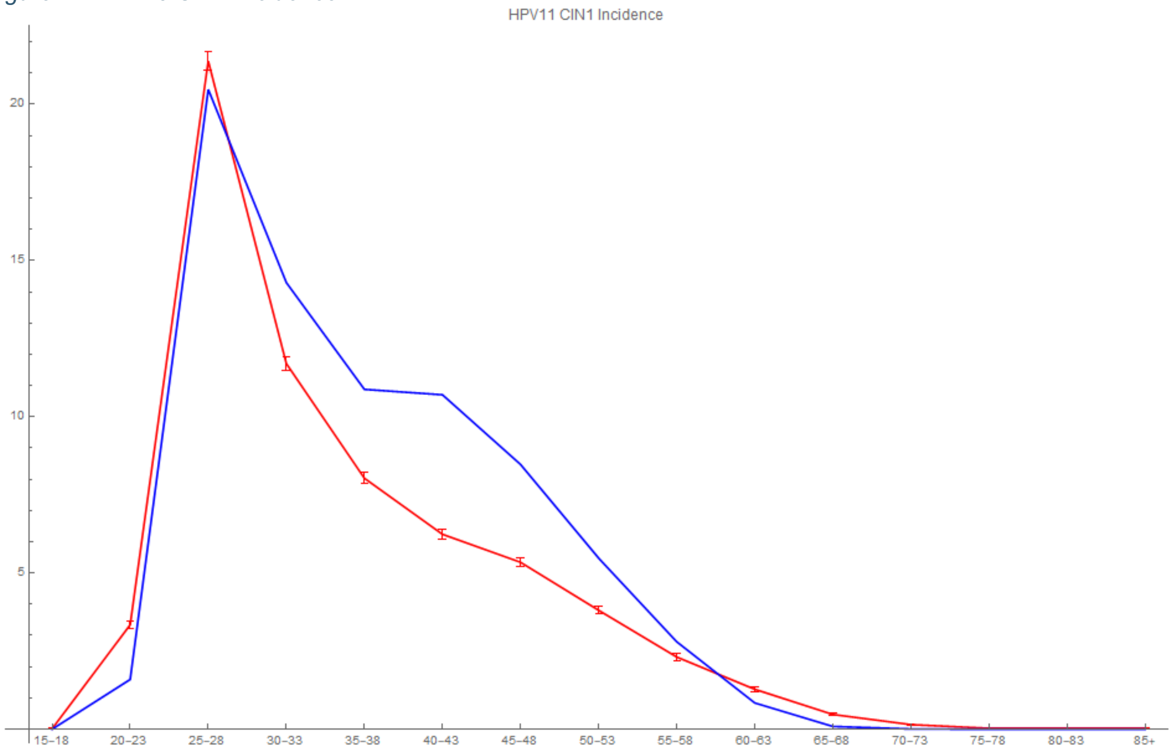

Figure 43. HPV 11 CIN1 incidence

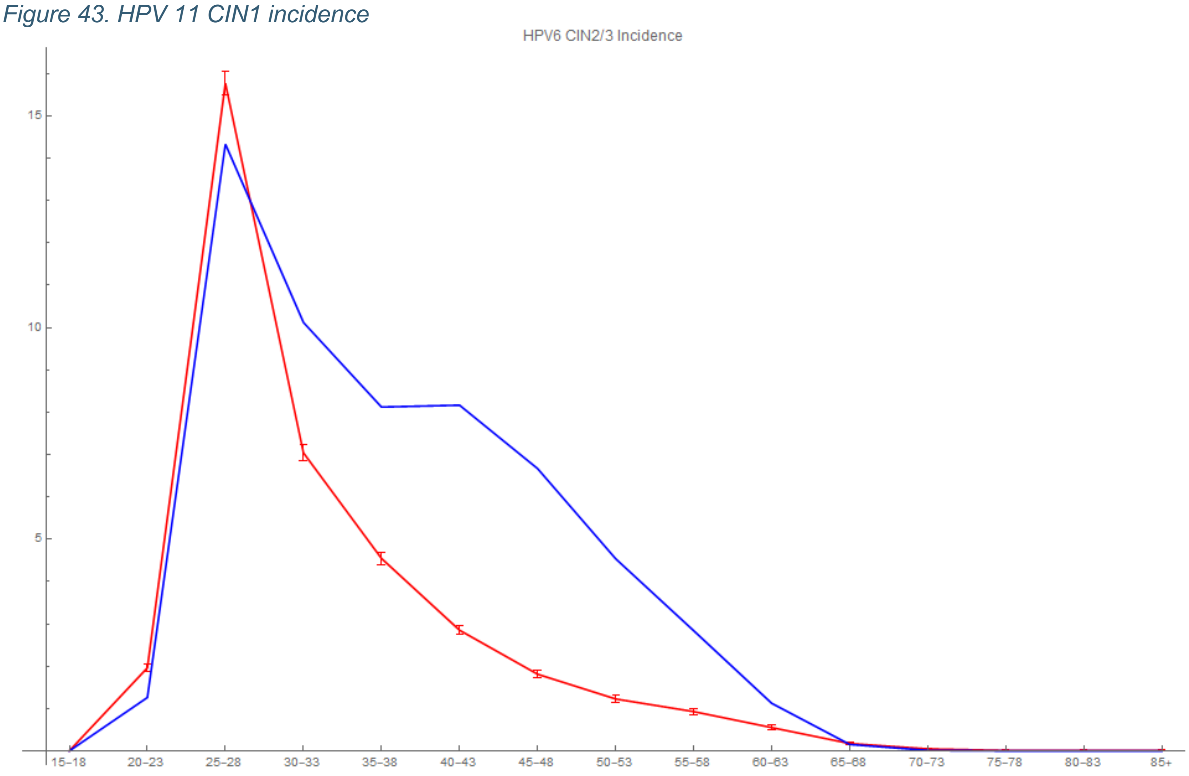

Figure 44. HPV 6 CIN2/3 incidence

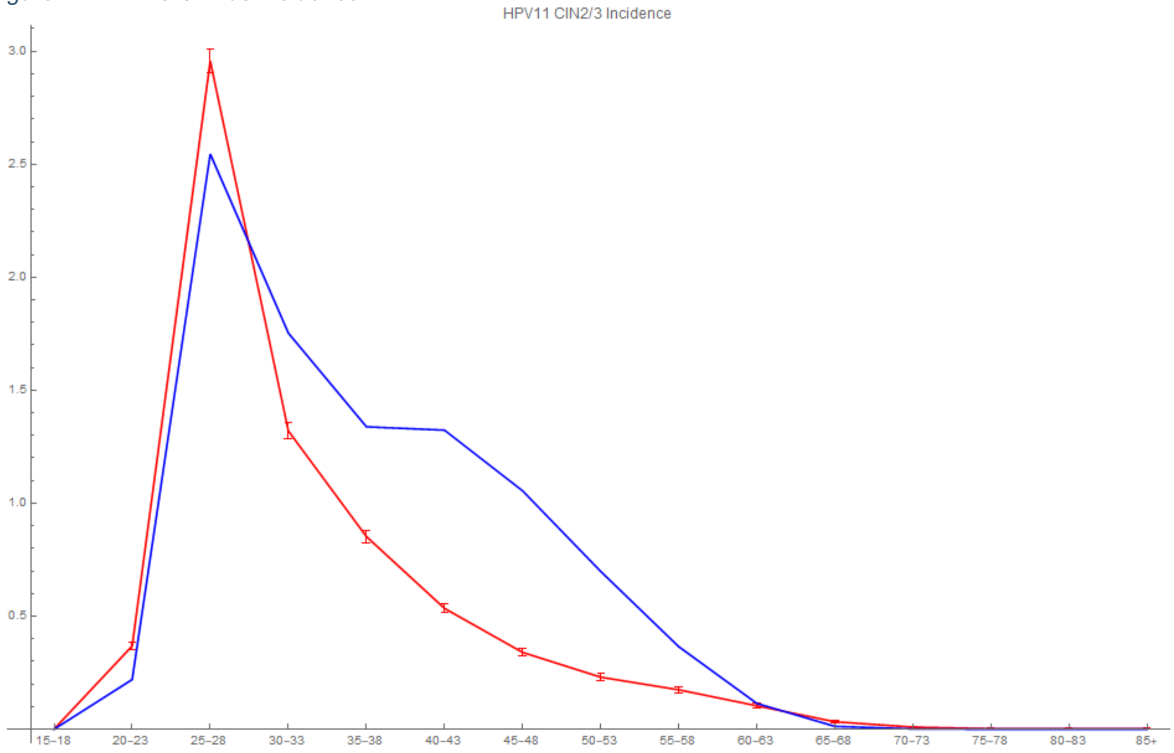

Figure 45. HPV 11 CIN2/3 incidence

11.4 Head and Neck

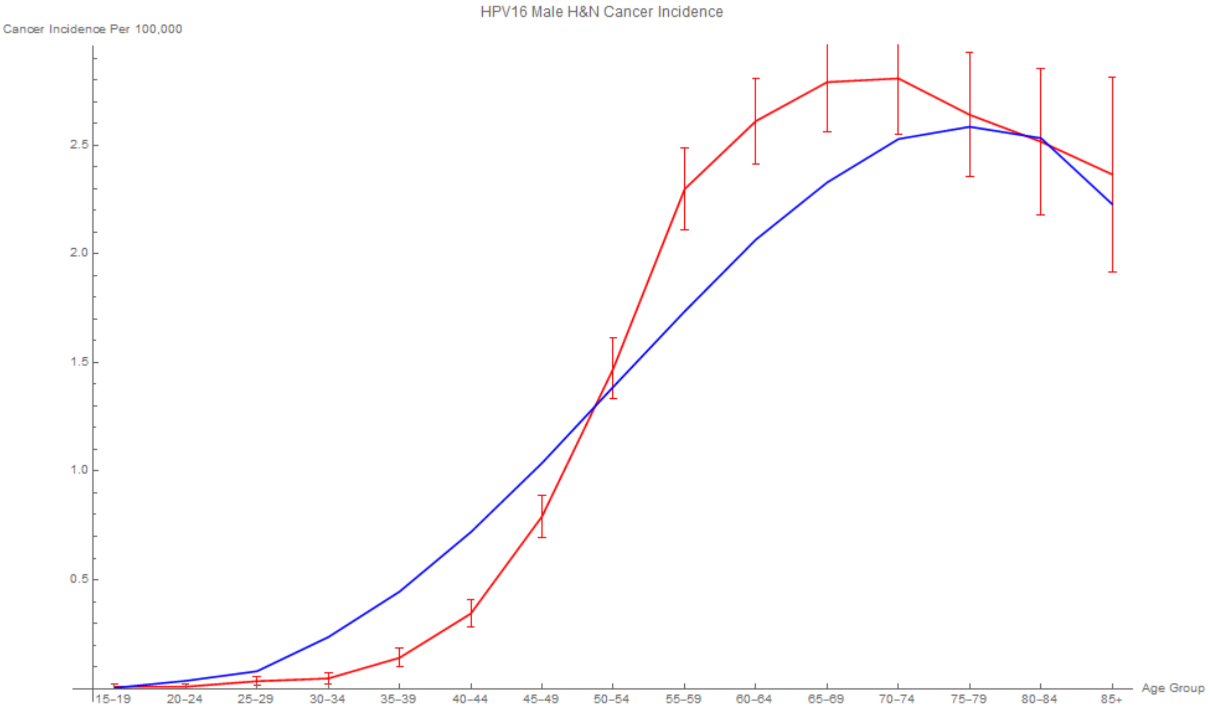

Figure 46. HPV 16 male head and neck cancer incidence

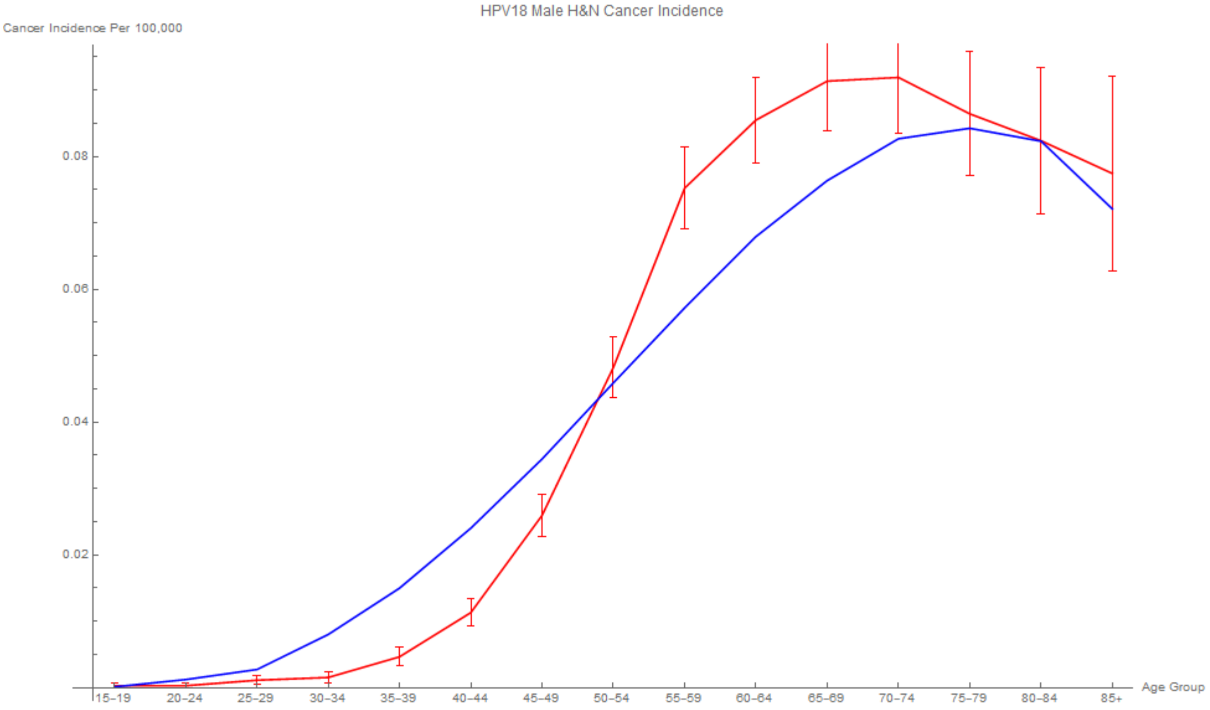

Figure 47. HPV 18 male head and neck cancer incidence

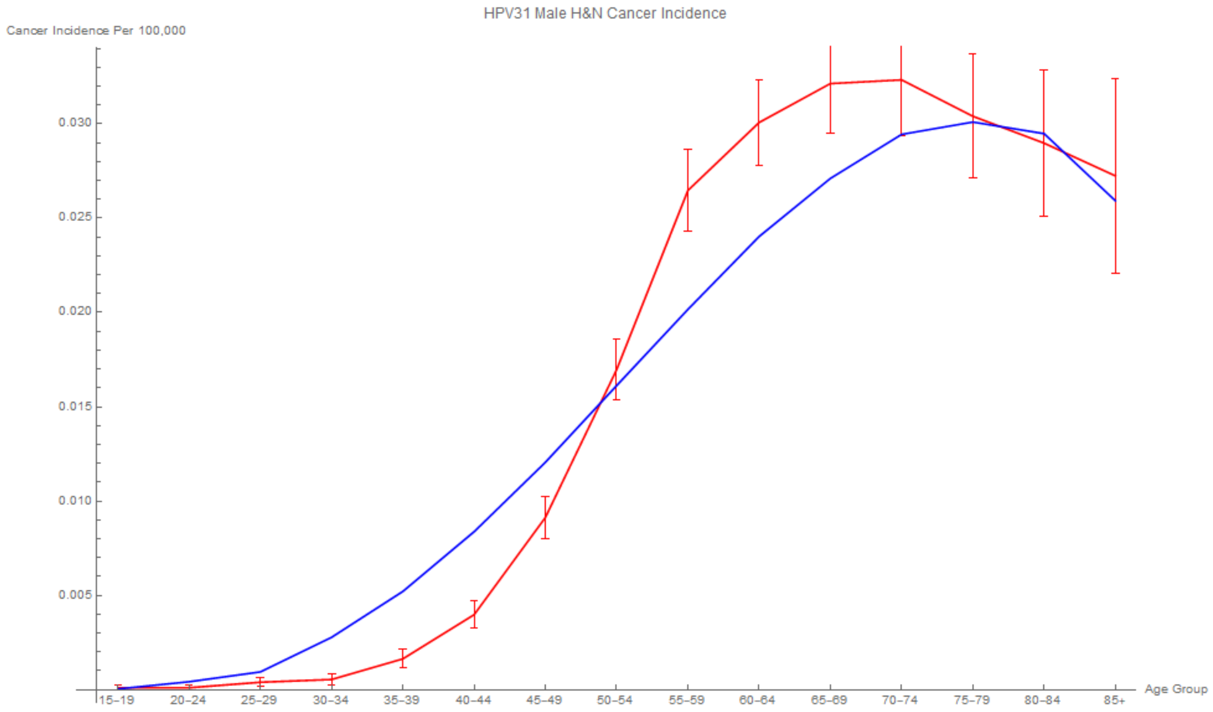

Figure 48. HPV 31 male head and neck cancer incidence

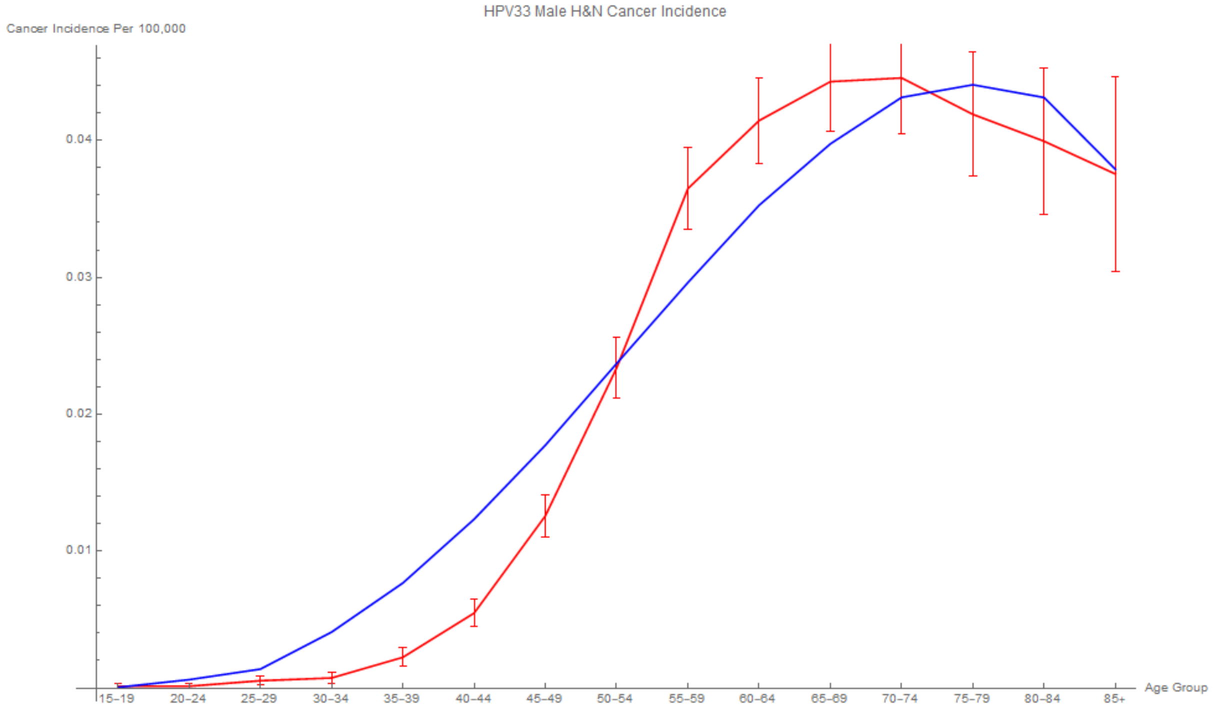

Figure 49. HPV 33 male head and neck cancer incidence

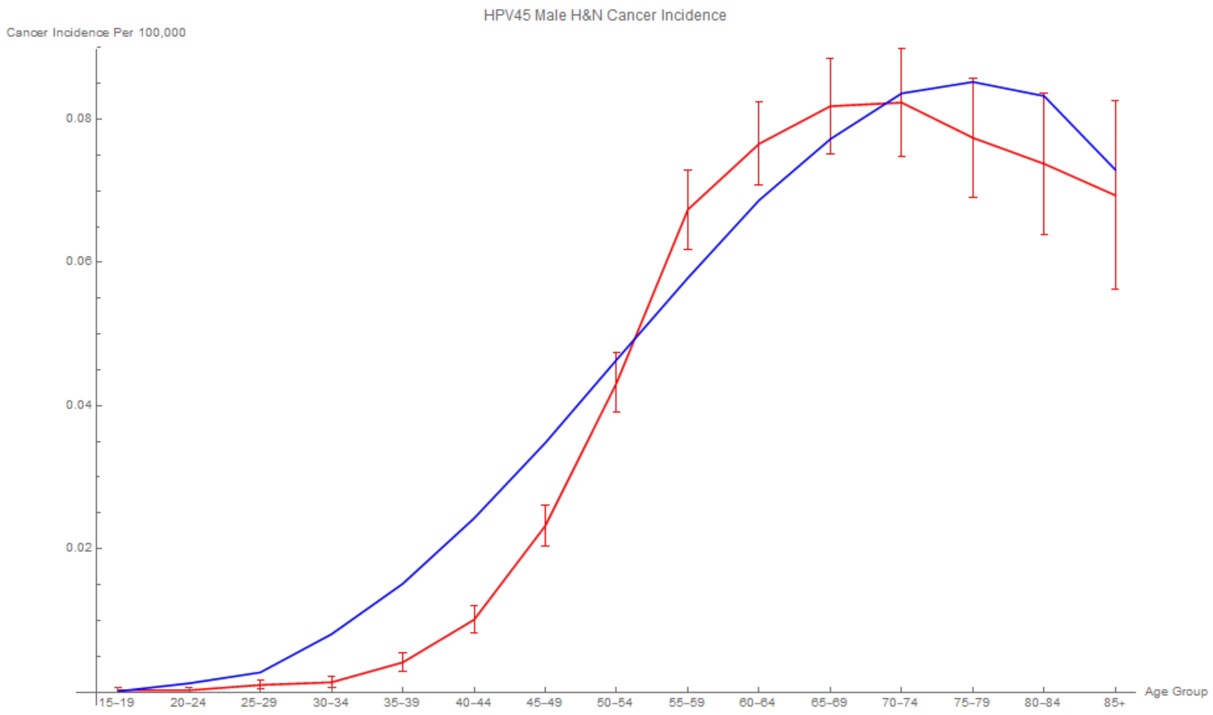

Figure 50. HPV 45 male head and neck cancer incidence

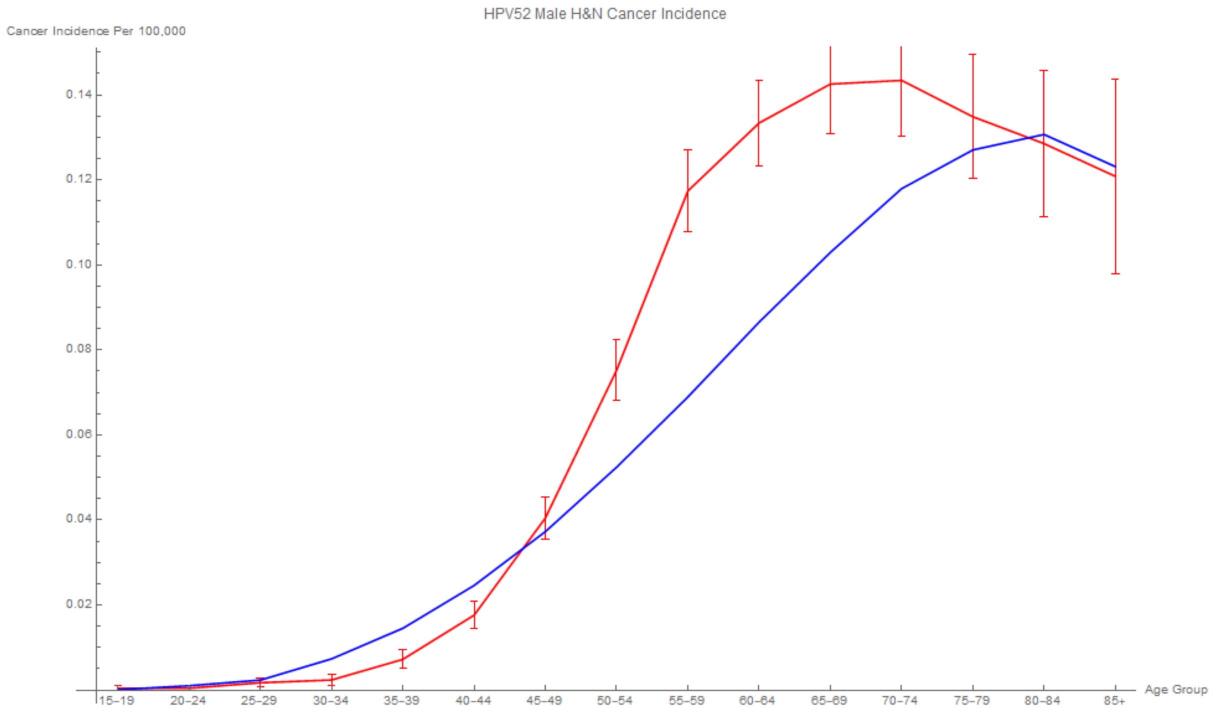

Figure 51. HPV 52 male head and neck cancer incidence

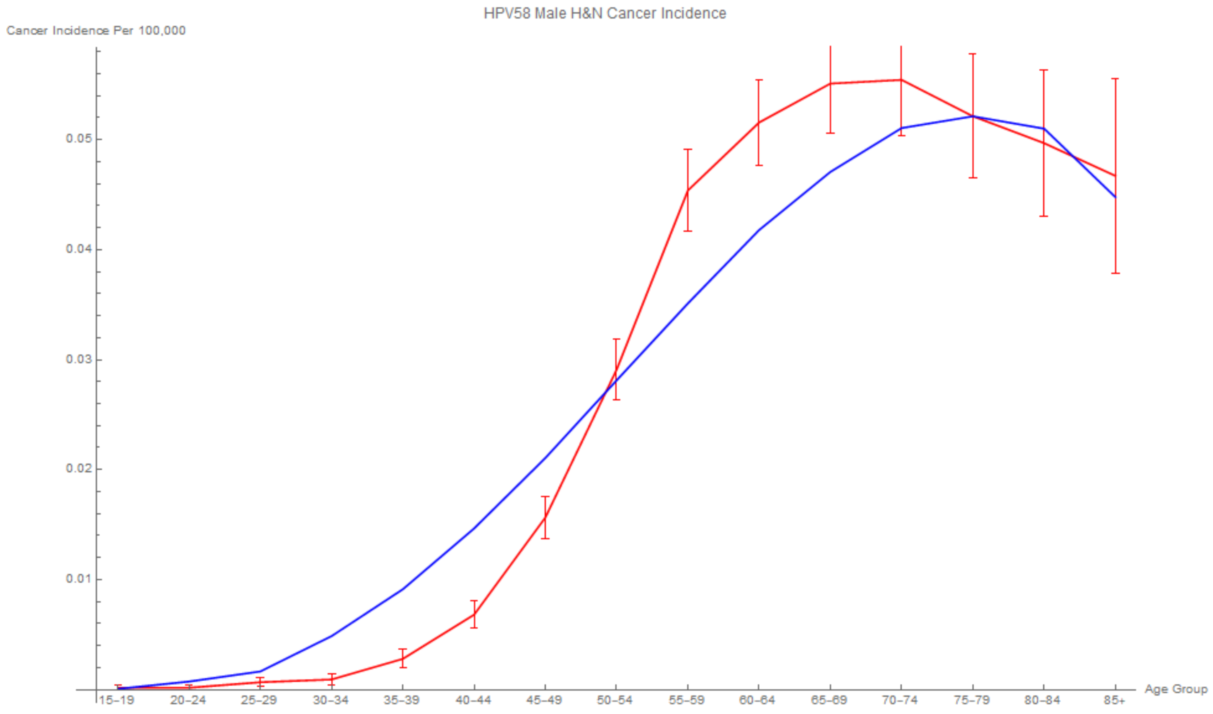

Figure 52. HPV 58 male head and neck cancer incidence

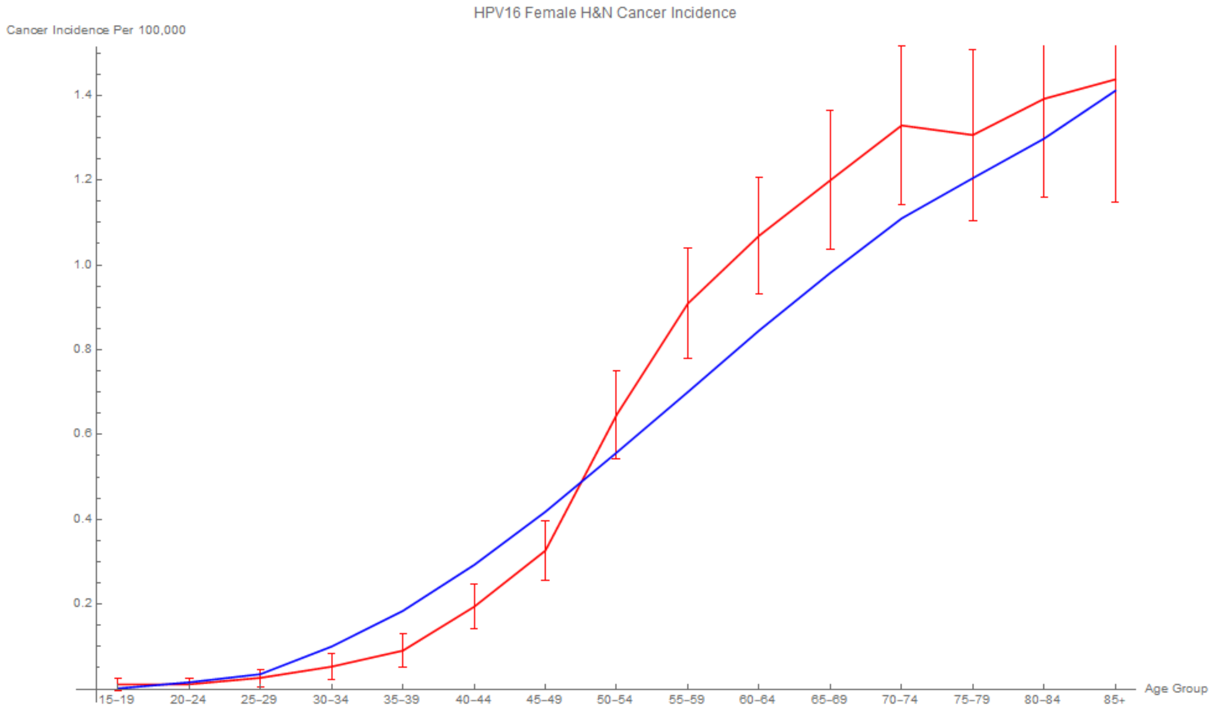

Figure 53. HPV 16 female head and neck cancer incidence

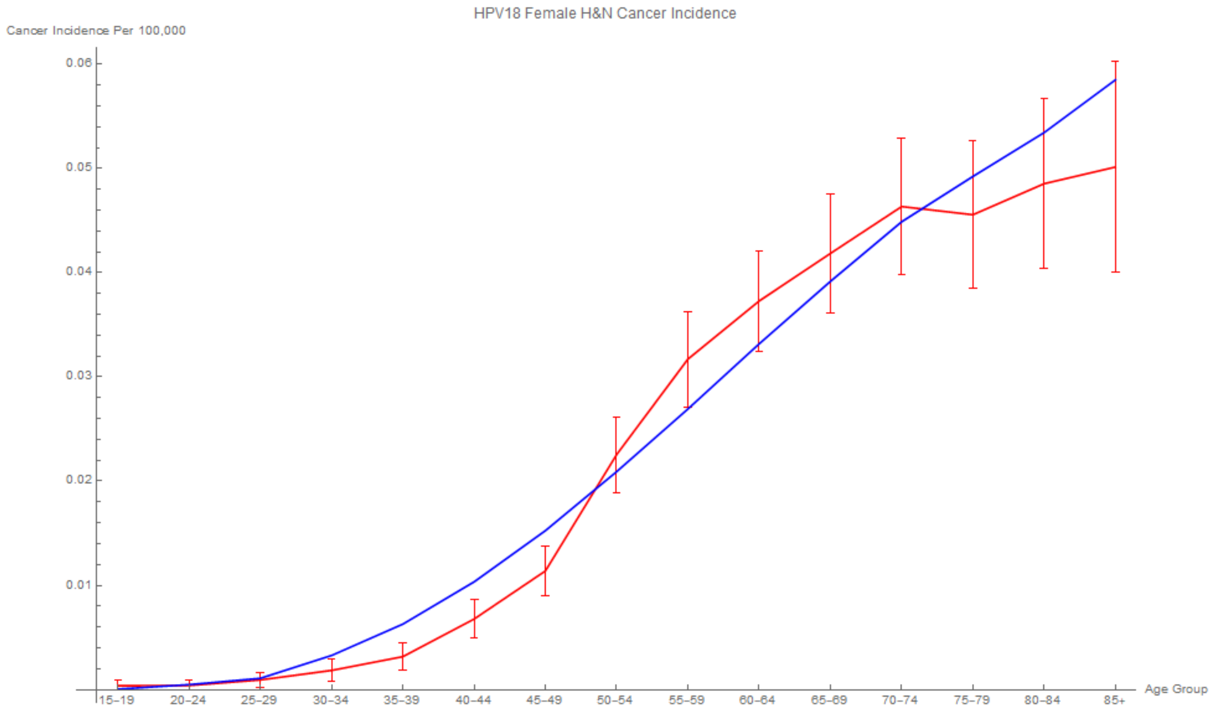

Figure 54. HPV 18 female head and neck cancer incidence

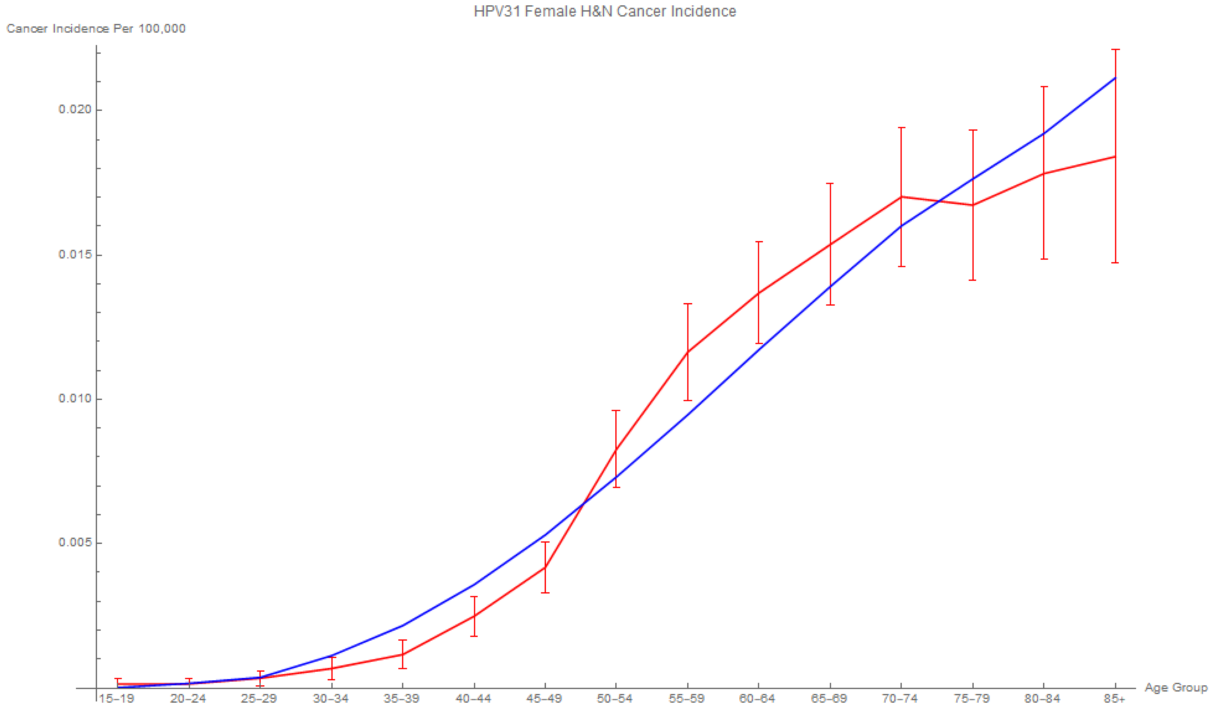

Figure 55. HPV 31 female head and neck cancer incidence

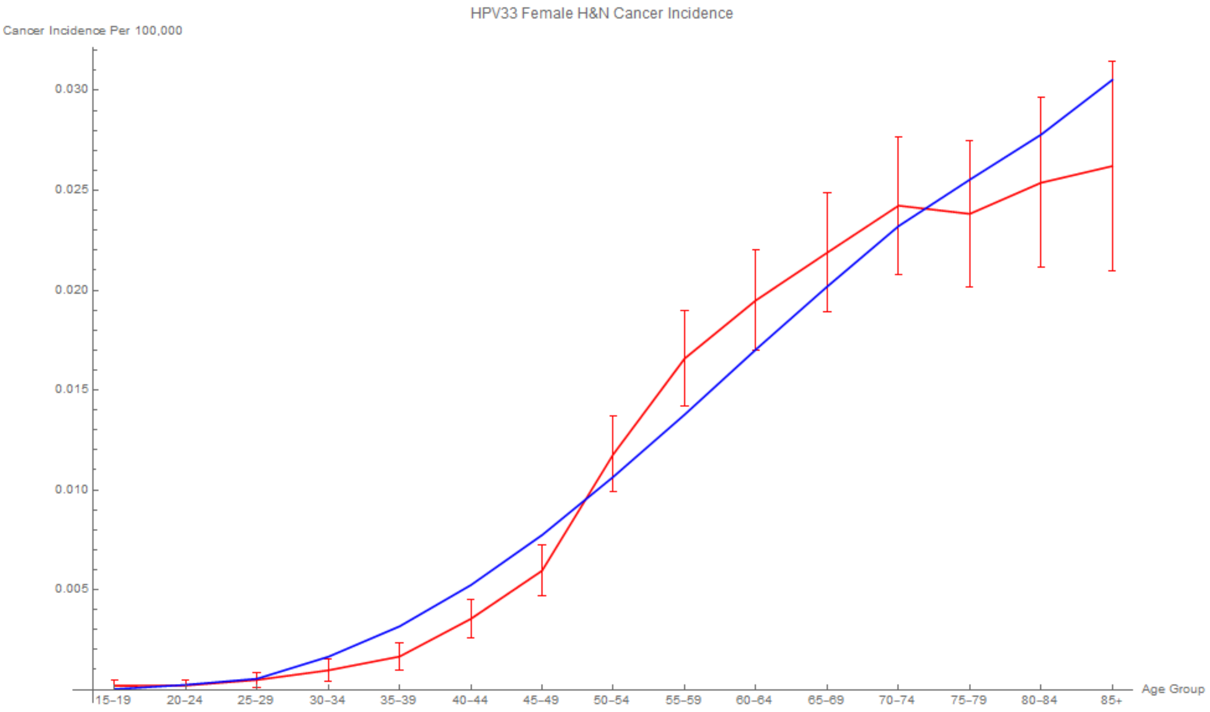

Figure 56. HPV 33 female head and neck cancer incidence

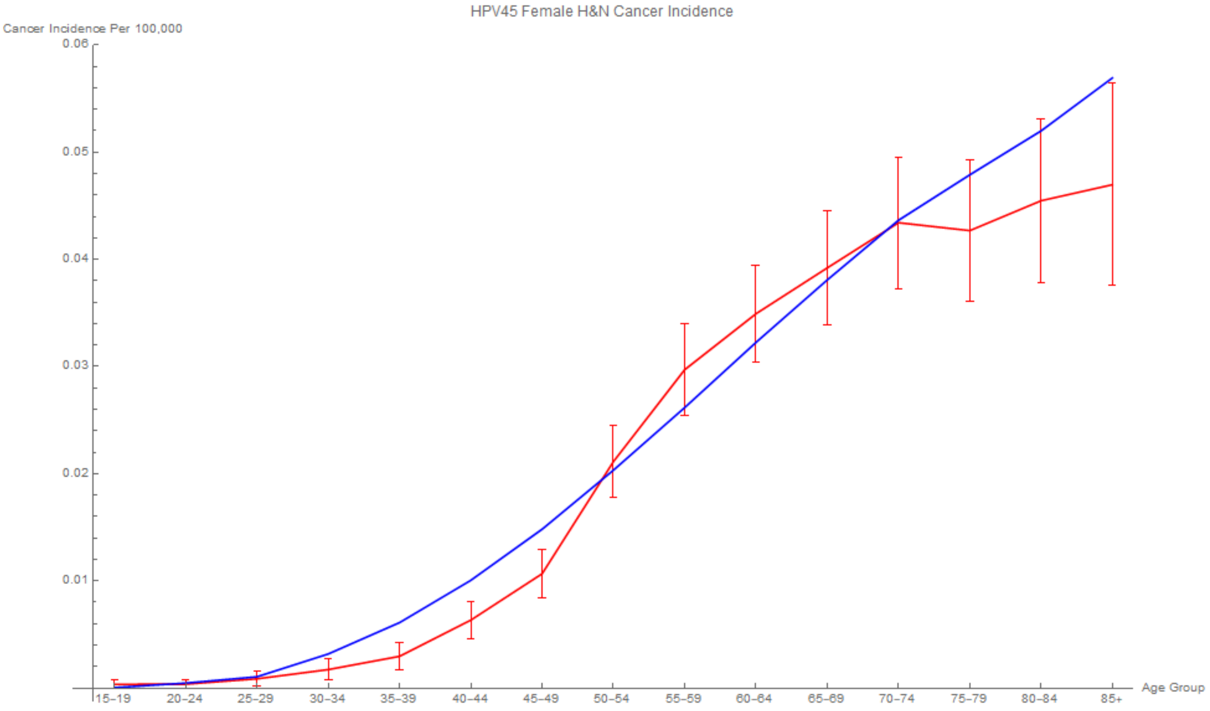

Figure 57. HPV 45 female head and neck cancer incidence

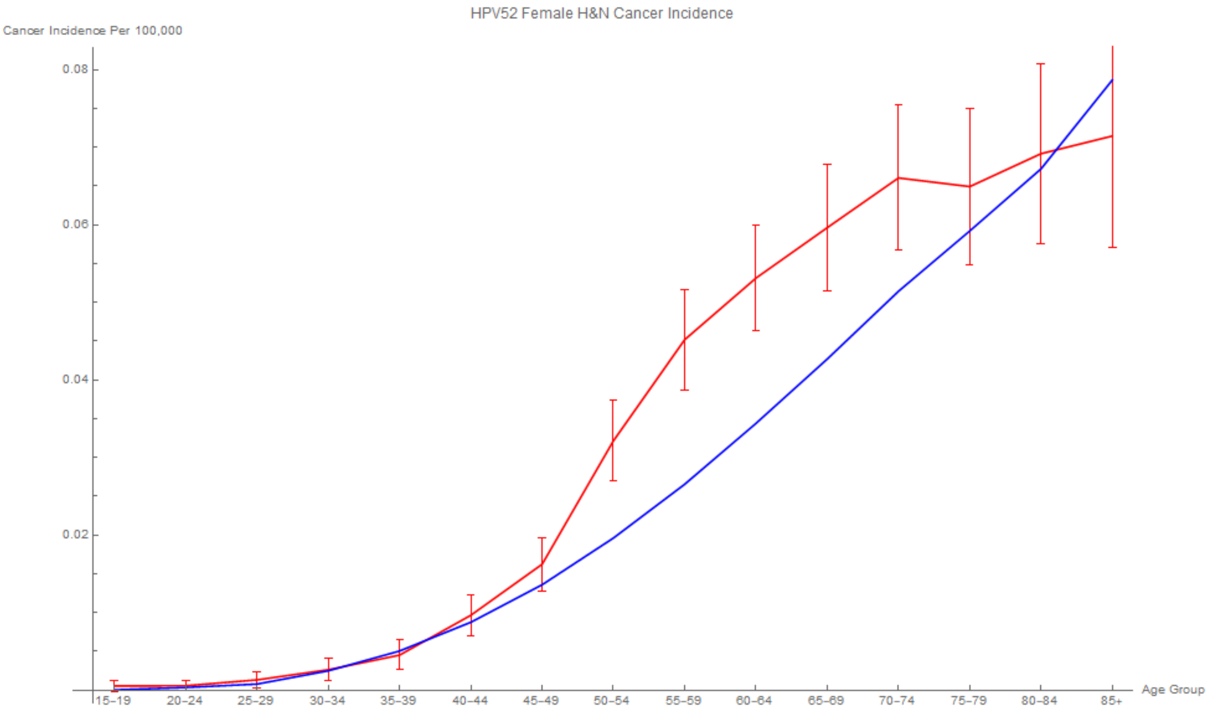

Figure 58. HPV 52 female head and neck cancer incidence

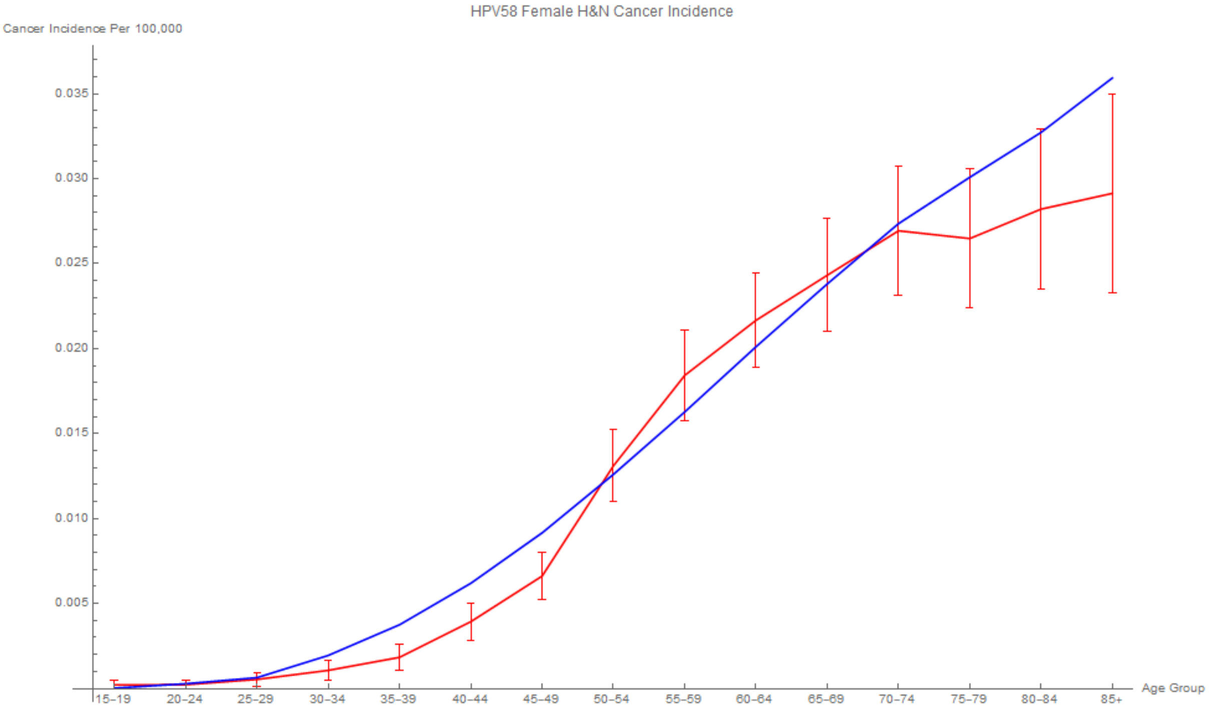

Figure 59. HPV 58 female head and neck cancer incidence

11.5 Penile

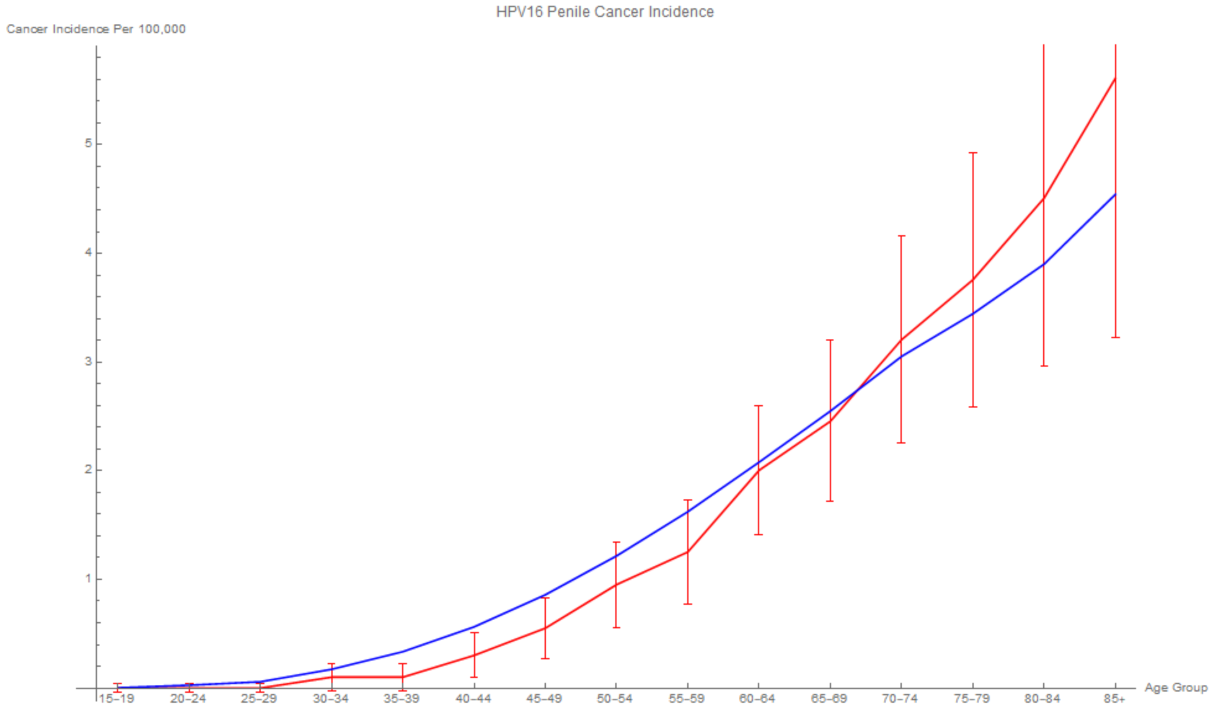

Figure 60. HPV 16 penile cancer incidence

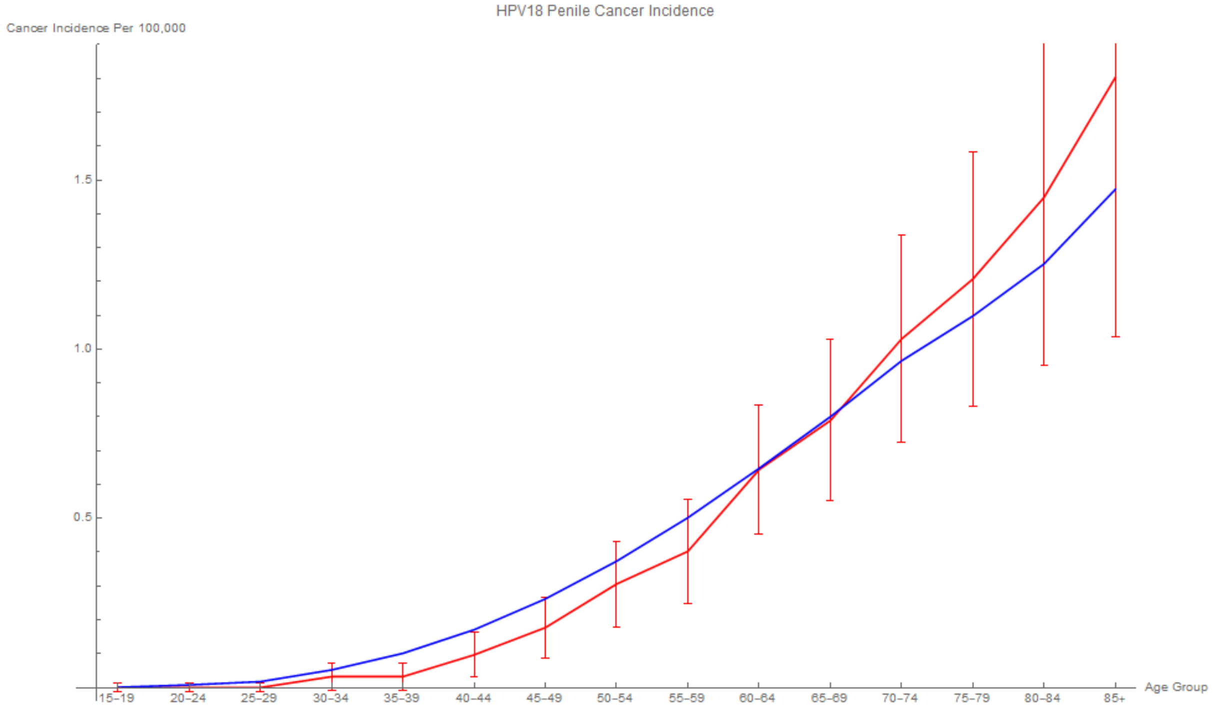

Figure 61. HPV 18 penile cancer incidence

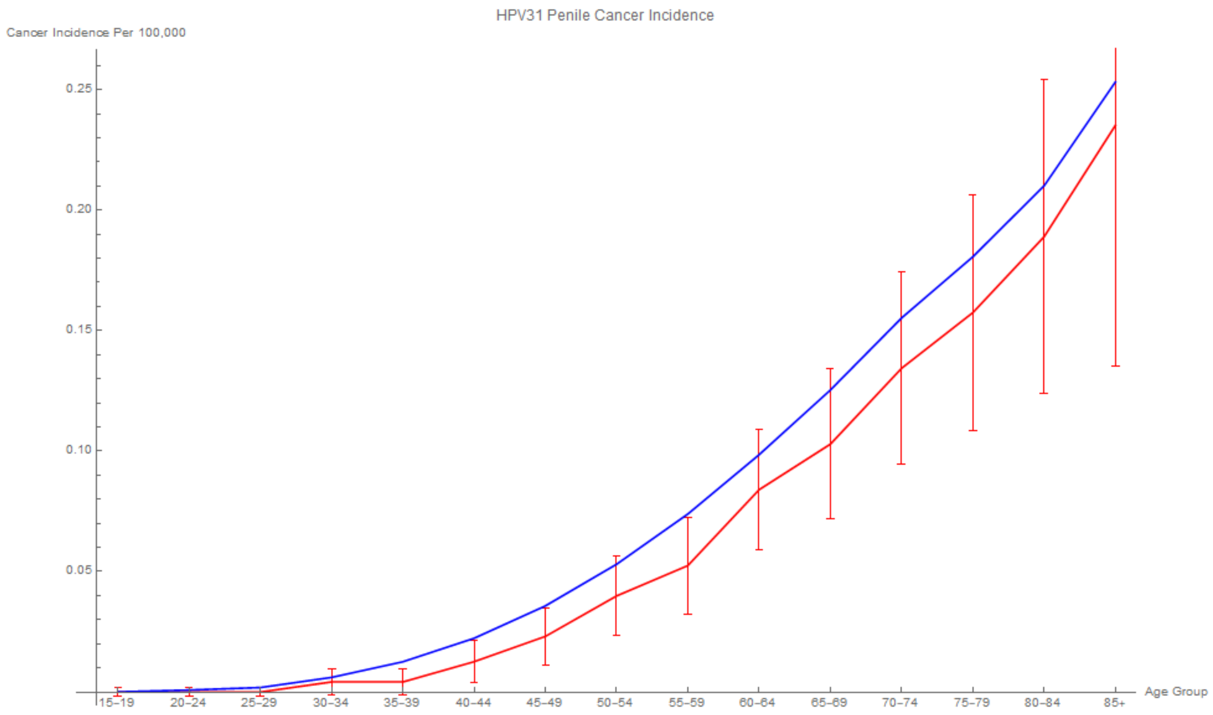

Figure 62. HPV 31 penile cancer incidence

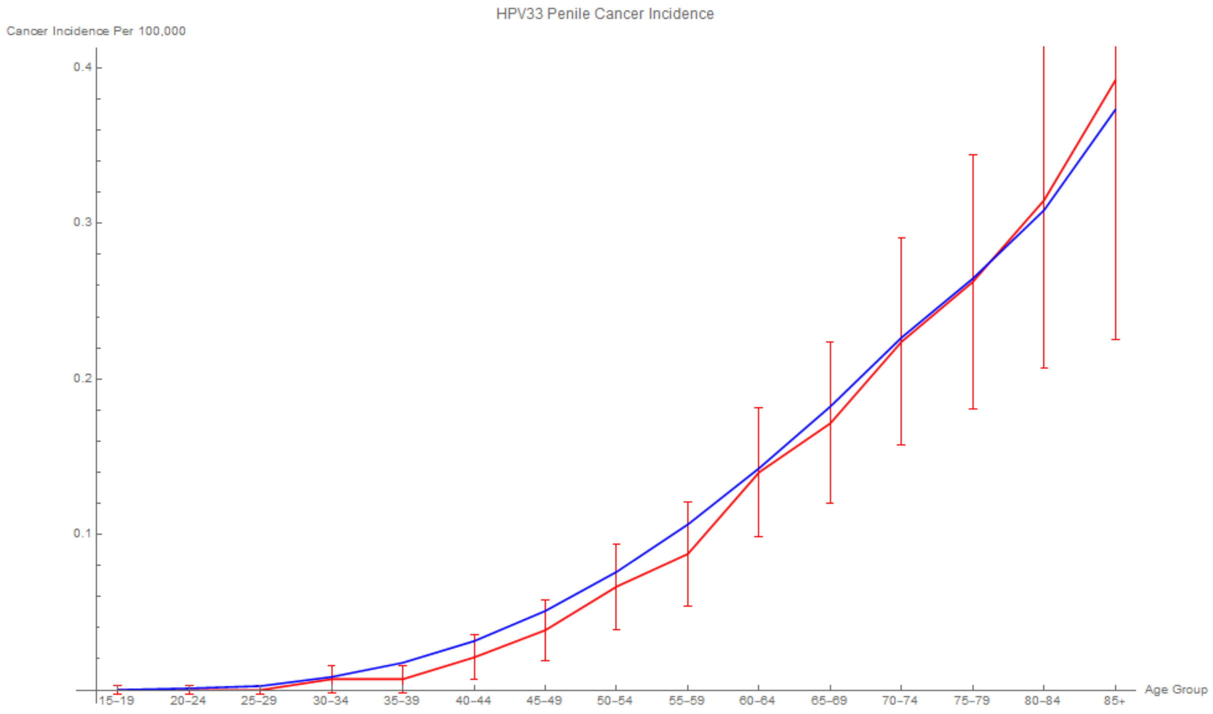

Figure 63. HPV 33 penile cancer incidence

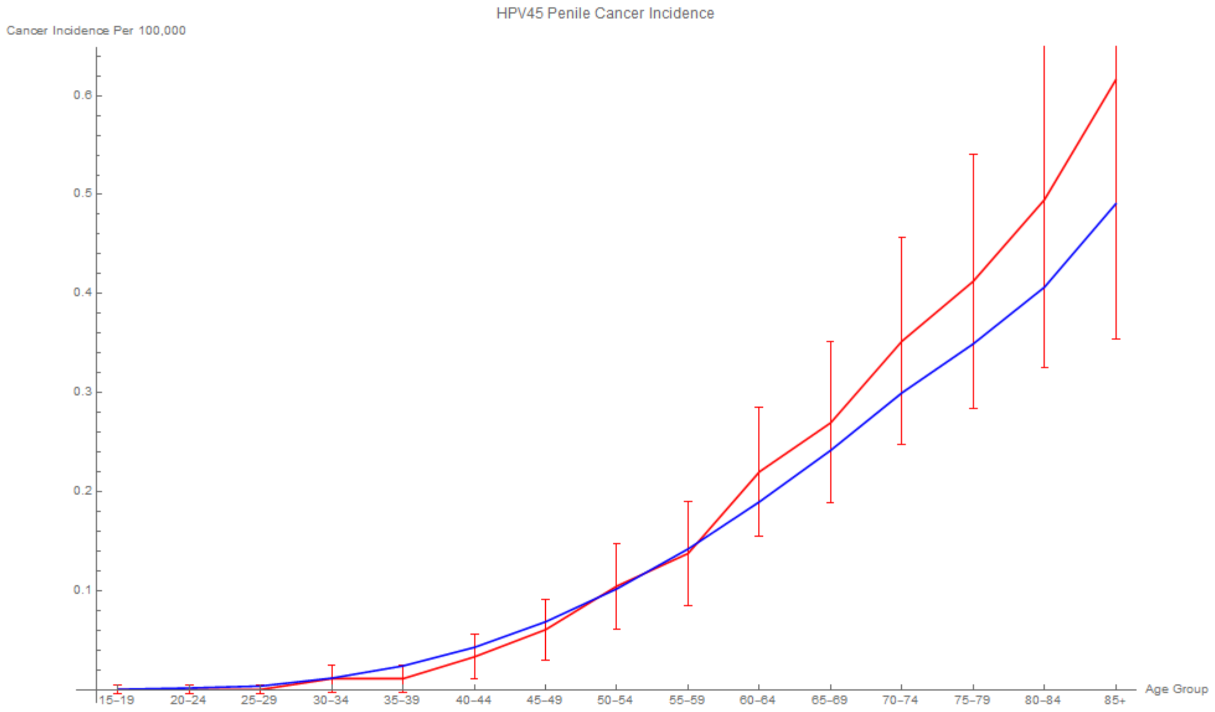

Figure 64. HPV 45 penile cancer incidence

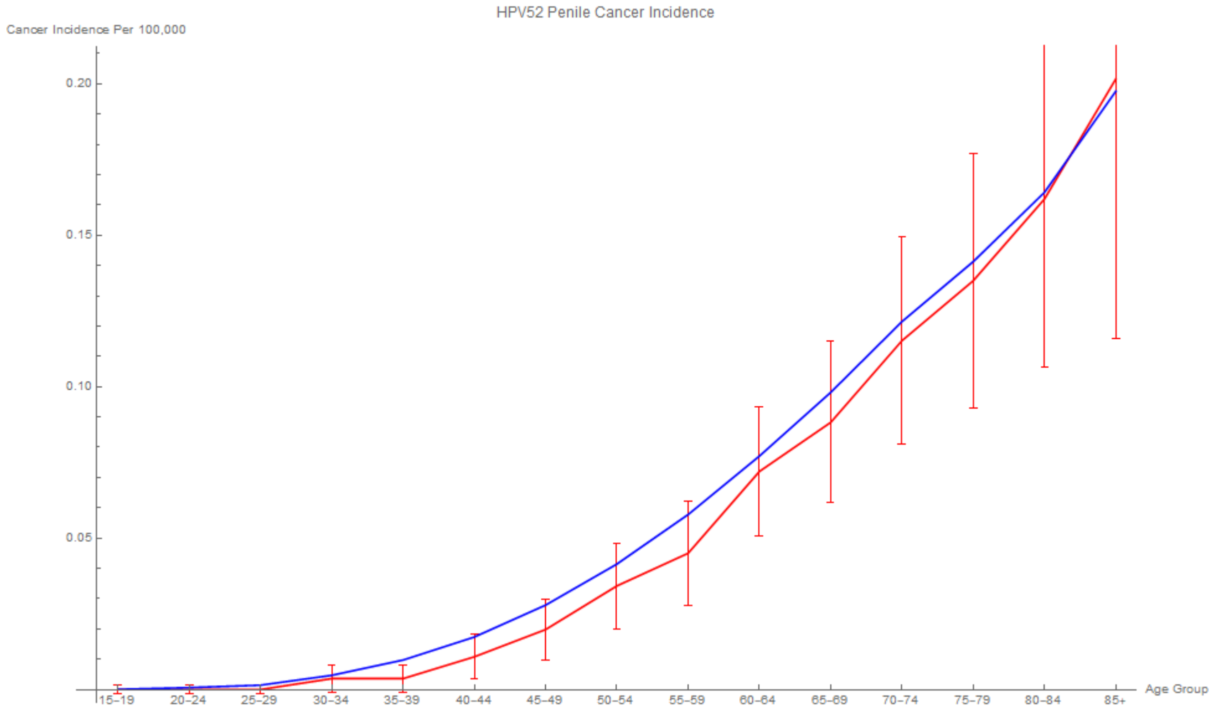

Figure 65. HPV 52 penile cancer incidence

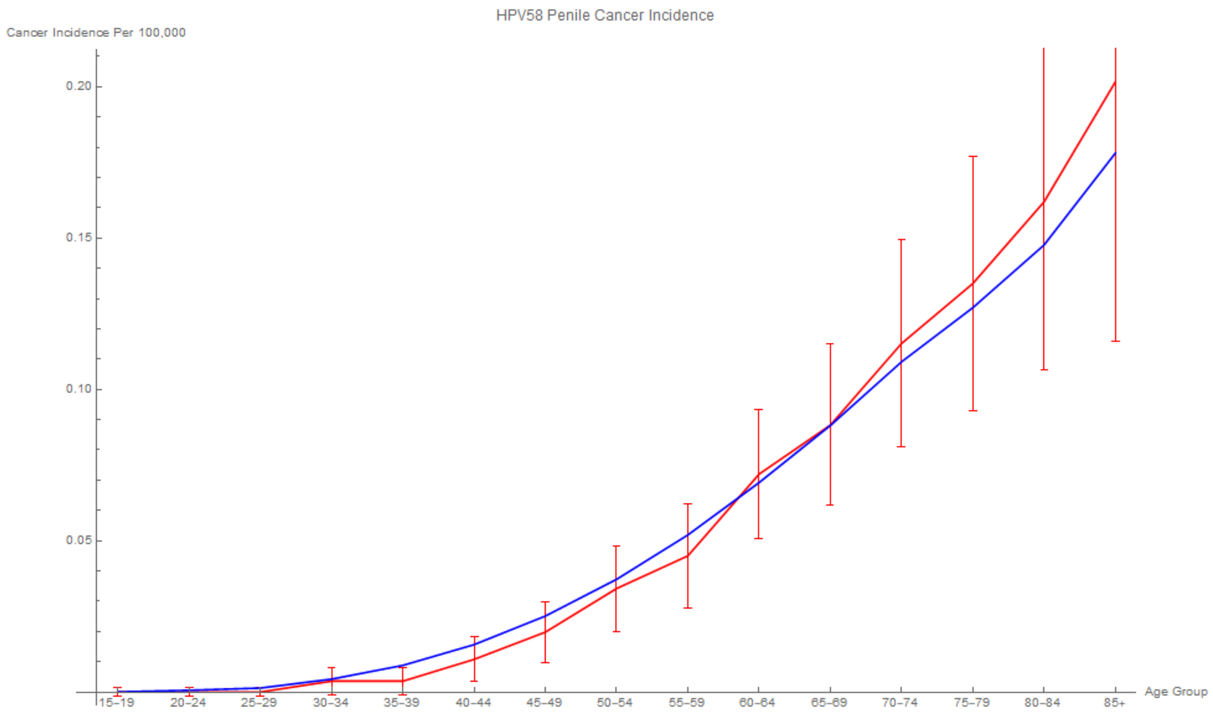

Figure 66. HPV 58 penile cancer incidence

11.6 Vaginal

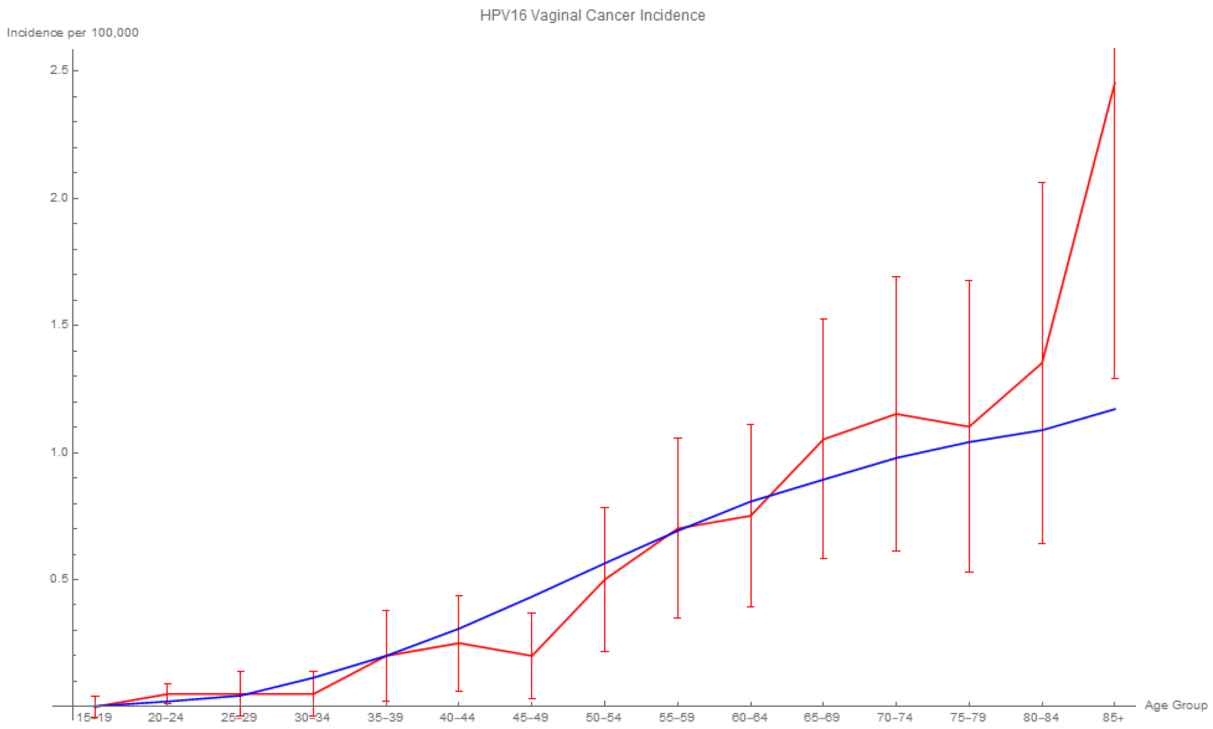

Figure 67. HPV 16 vaginal cancer incidence

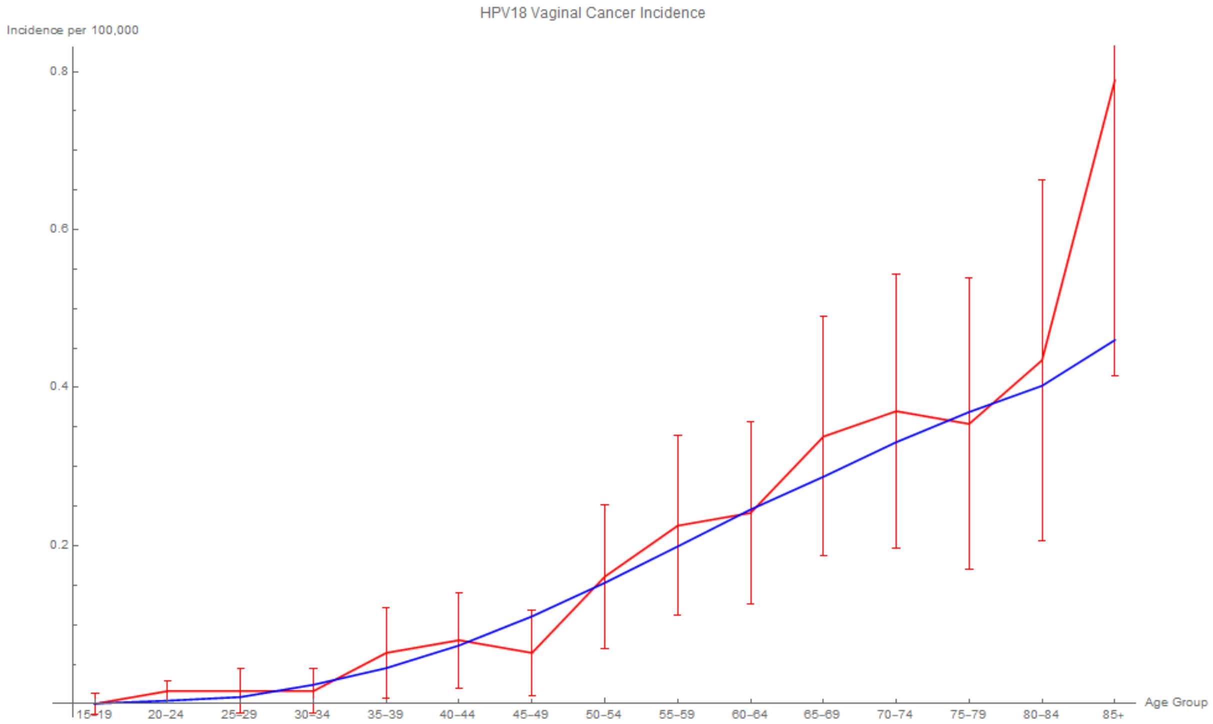

Figure 68. HPV 18 penile cancer incidence

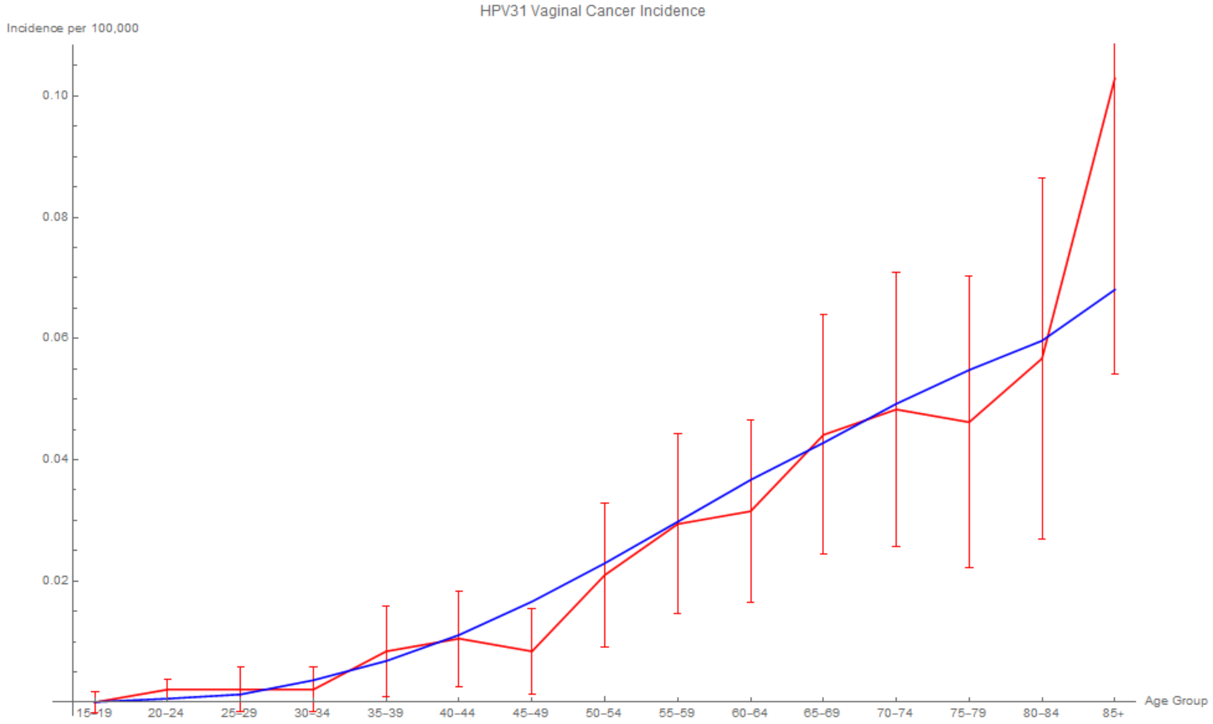

Figure 69. HPV 31 vaginal cancer incidence

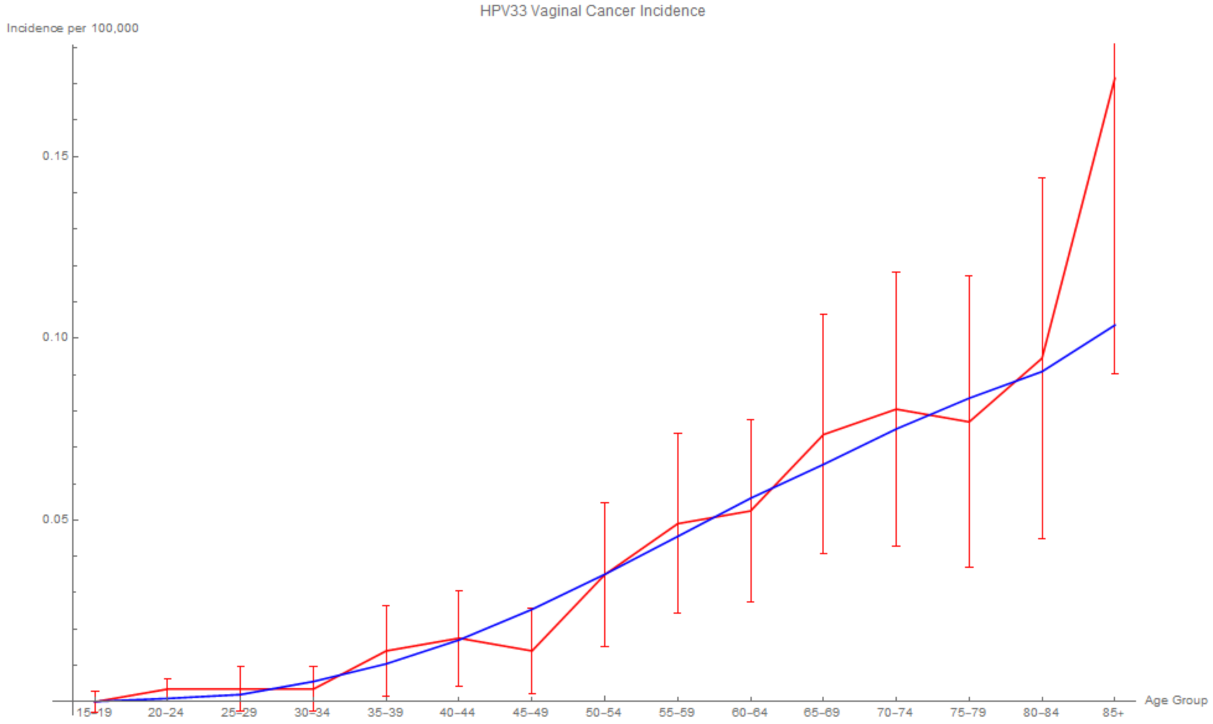

Figure 70. HPV 33 vaginal cancer incidence

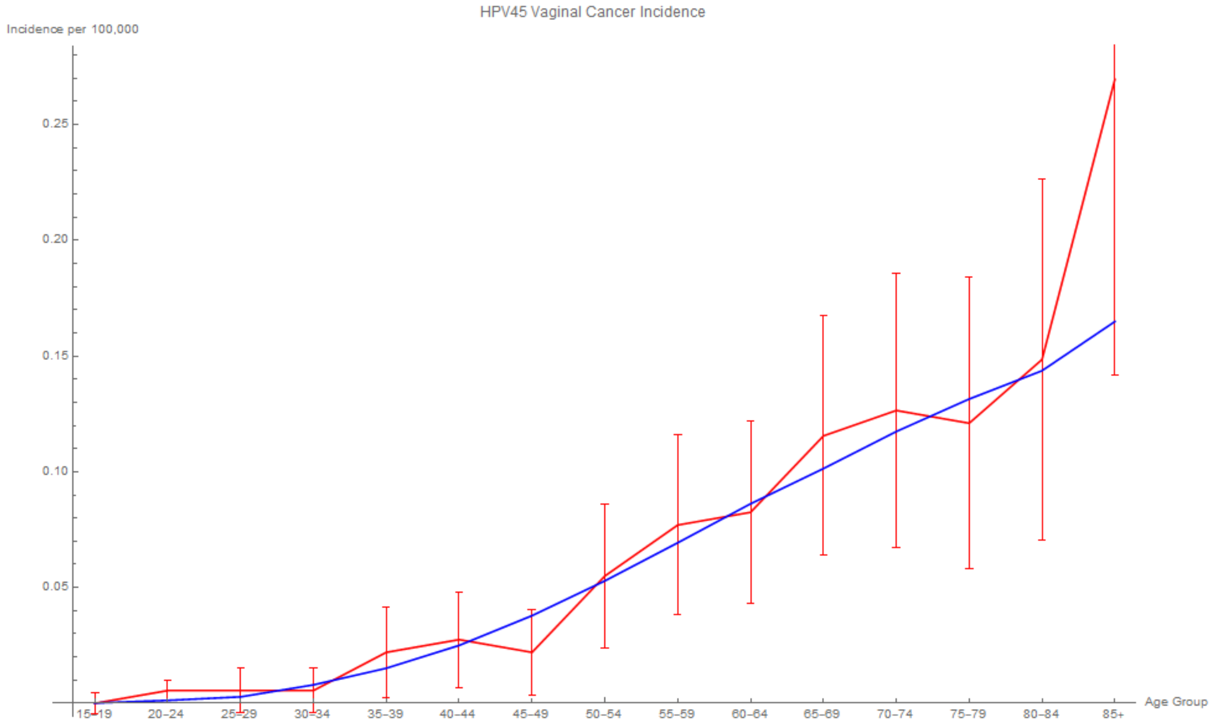

Figure 71. HPV 45 vaginal cancer incidence

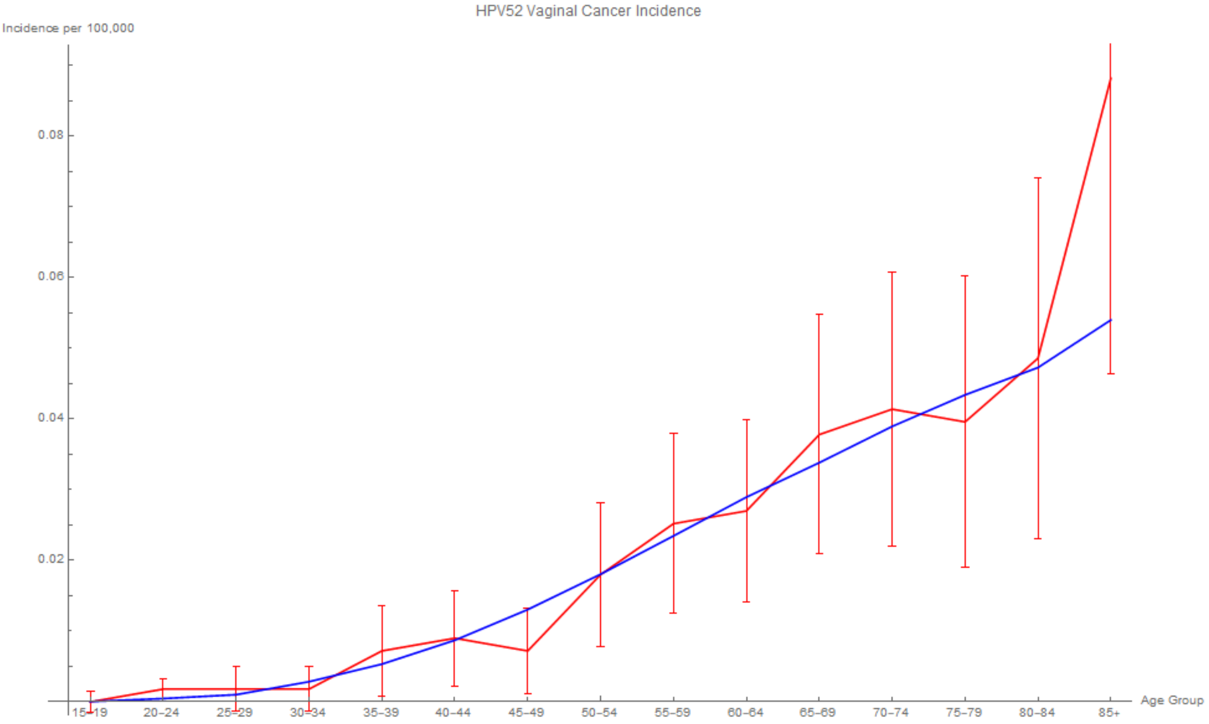

Figure 72. HPV 52 vaginal cancer incidence

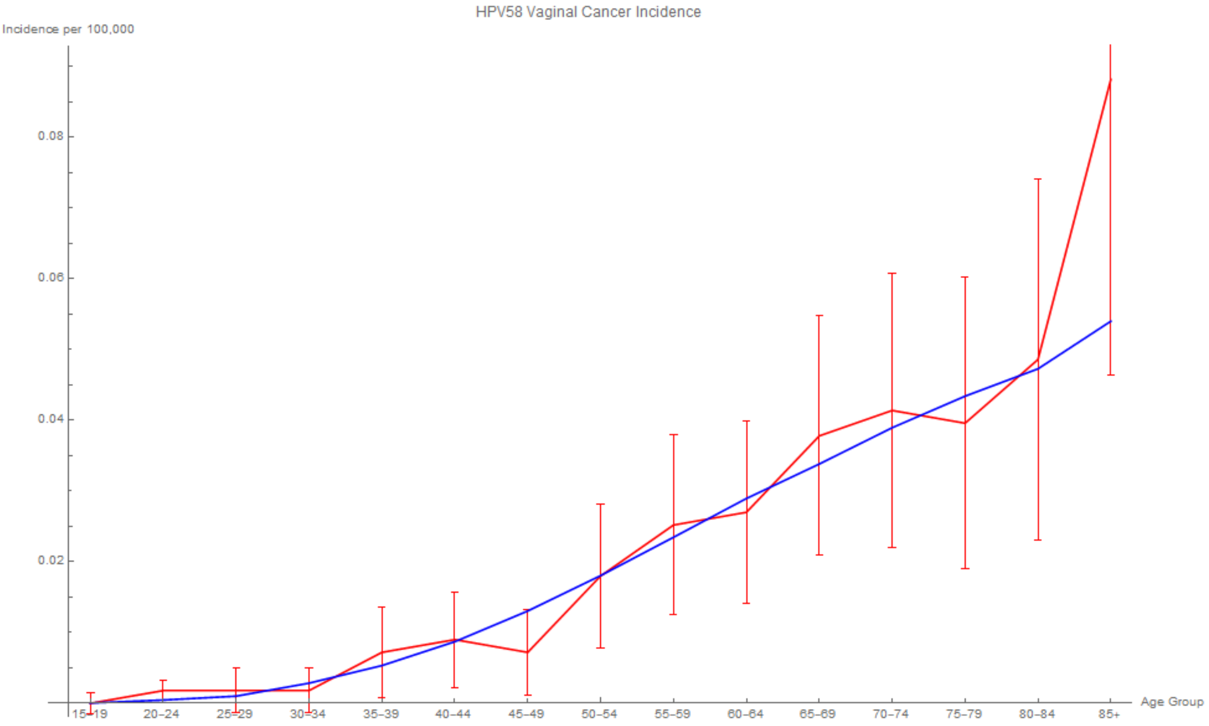

Figure 73. HPV 58 vaginal cancer incidence

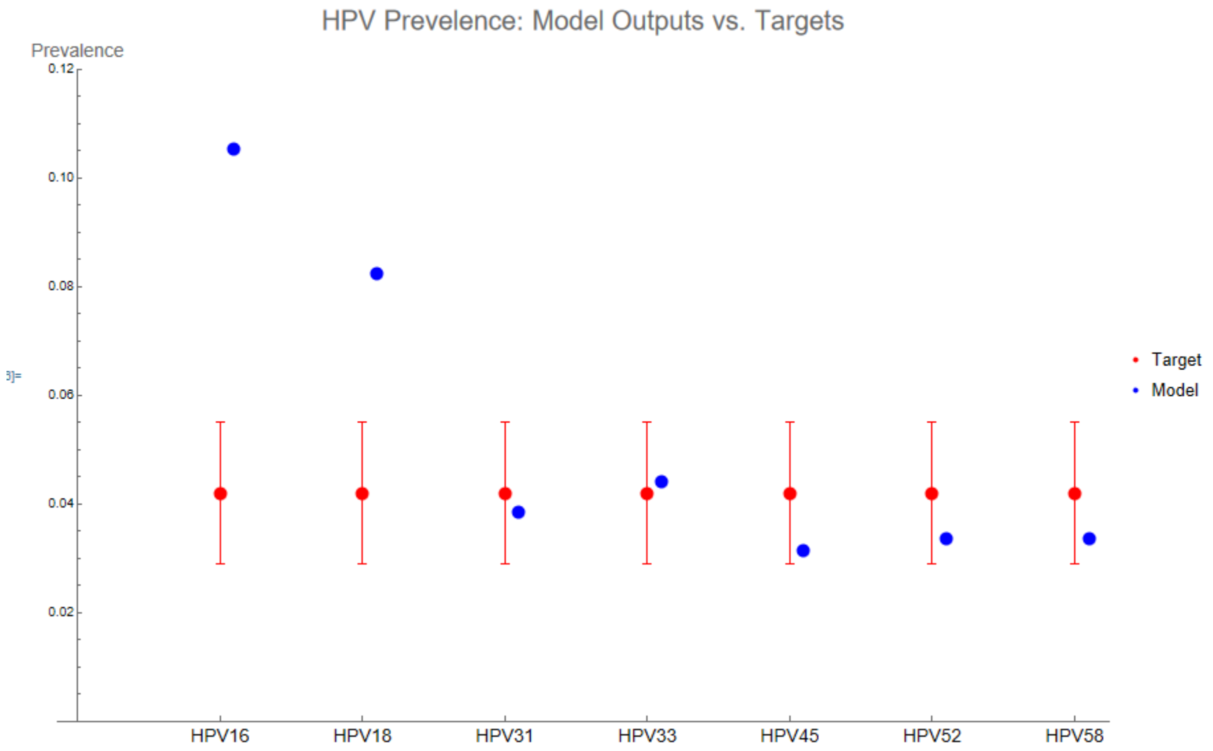

Figure 74. Vaginal infection prevalence by HPV type

11.7 Vulvar

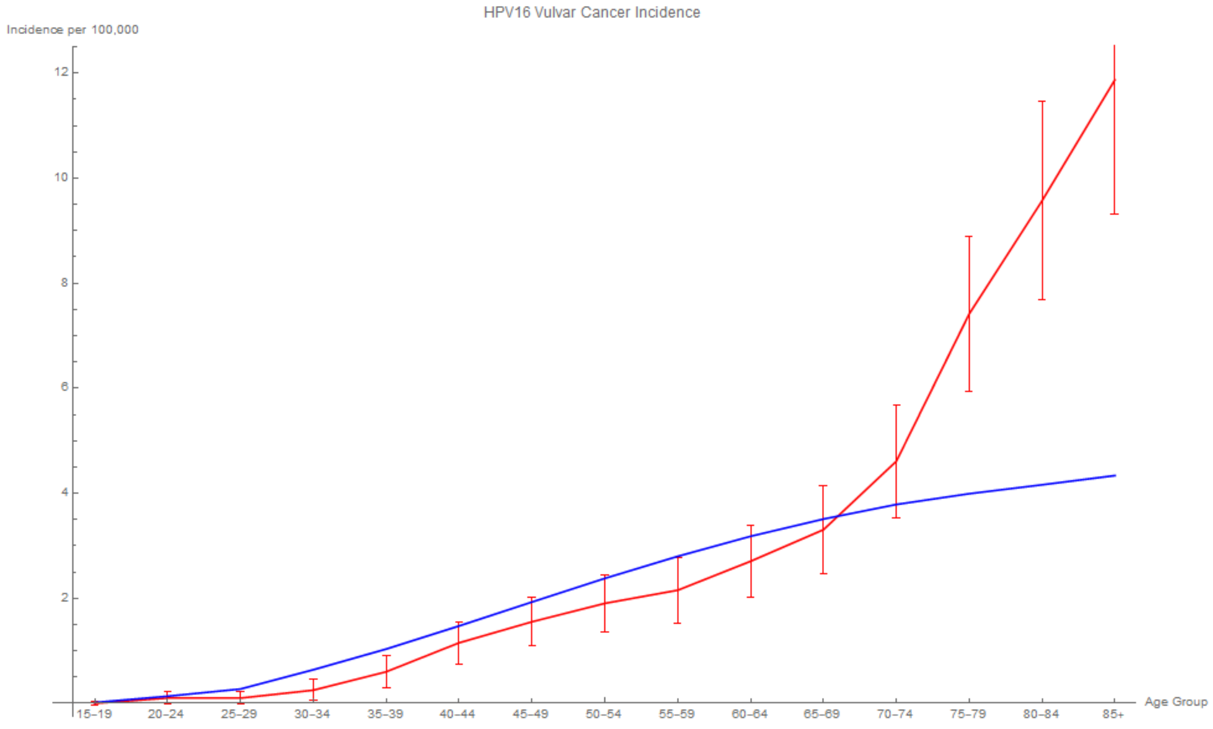

Figure 75. HPV 16 vulvar cancer incidence

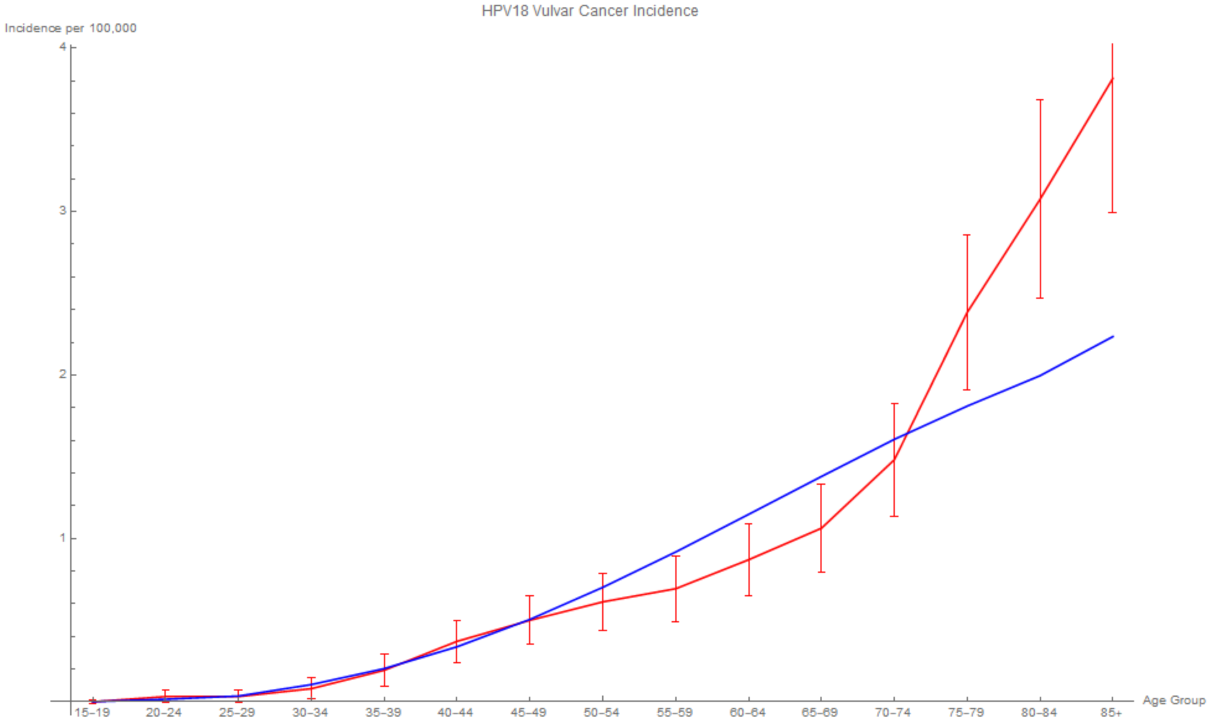

Figure 76. HPV 18 vulvar cancer incidence

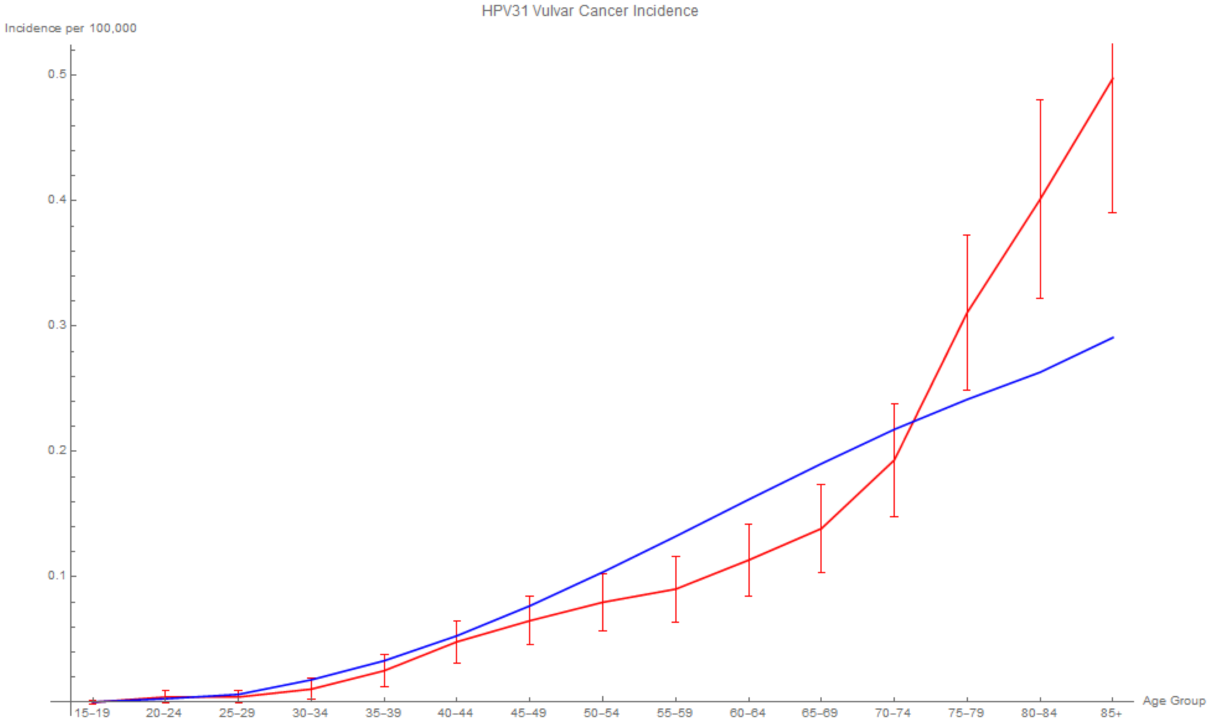

Figure 77. HPV 31 vulvar cancer incidence

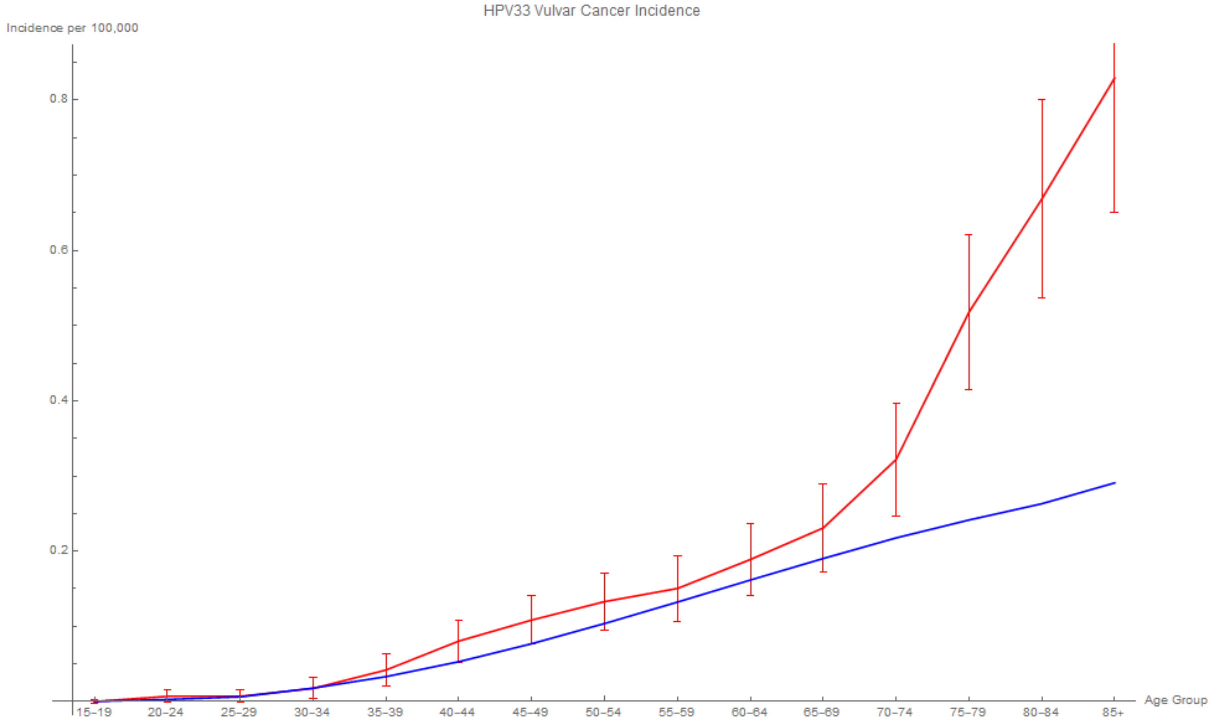

Figure 78. HPV 33 vulvar cancer incidence

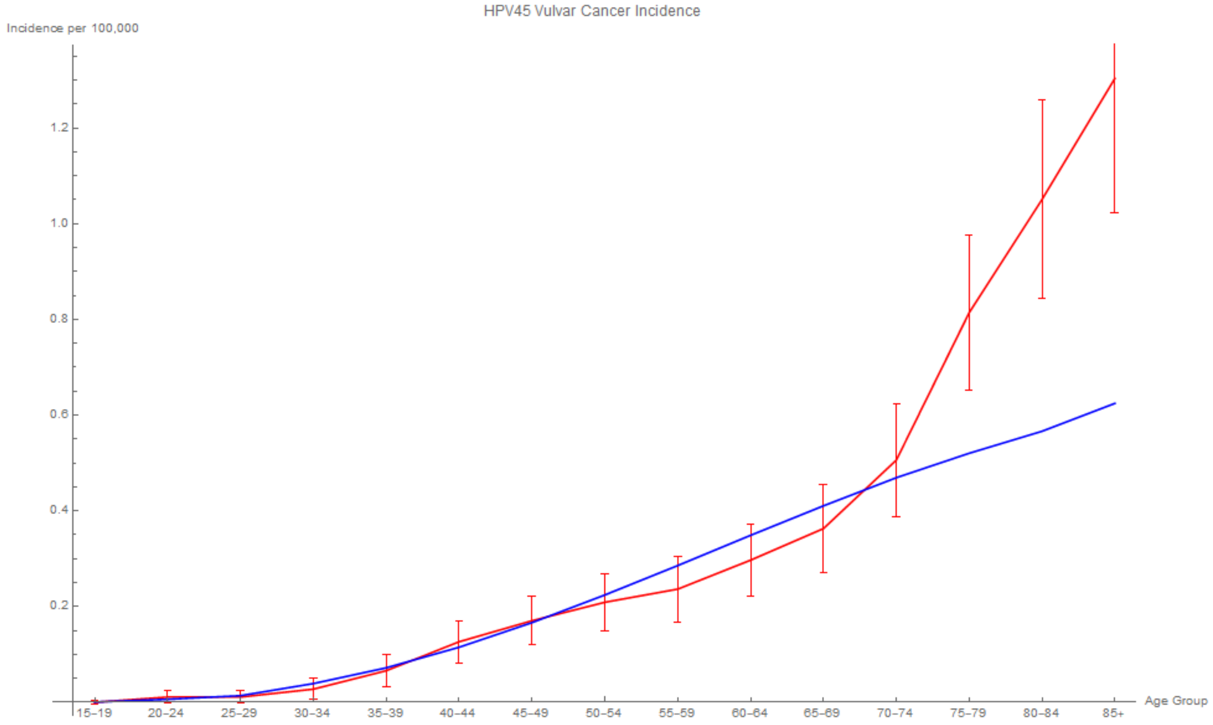

Figure 79. HPV 45 vulvar cancer incidence

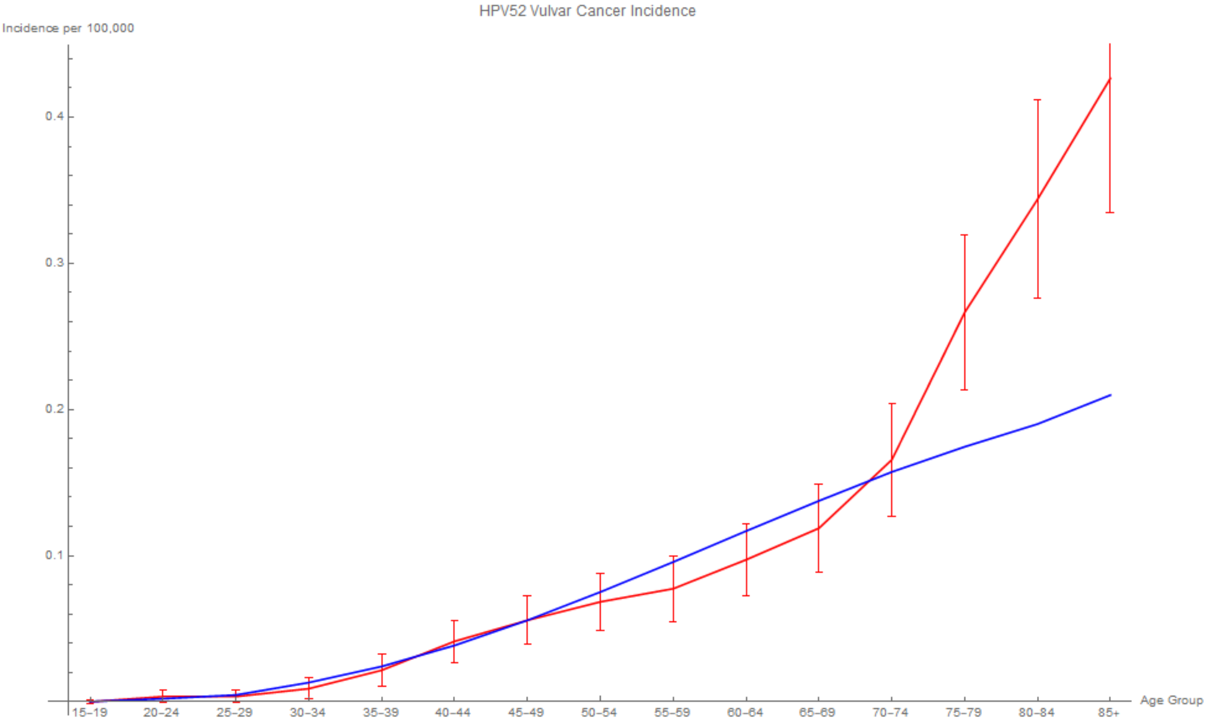

Figure 80. HPV 52 vulvar cancer incidence

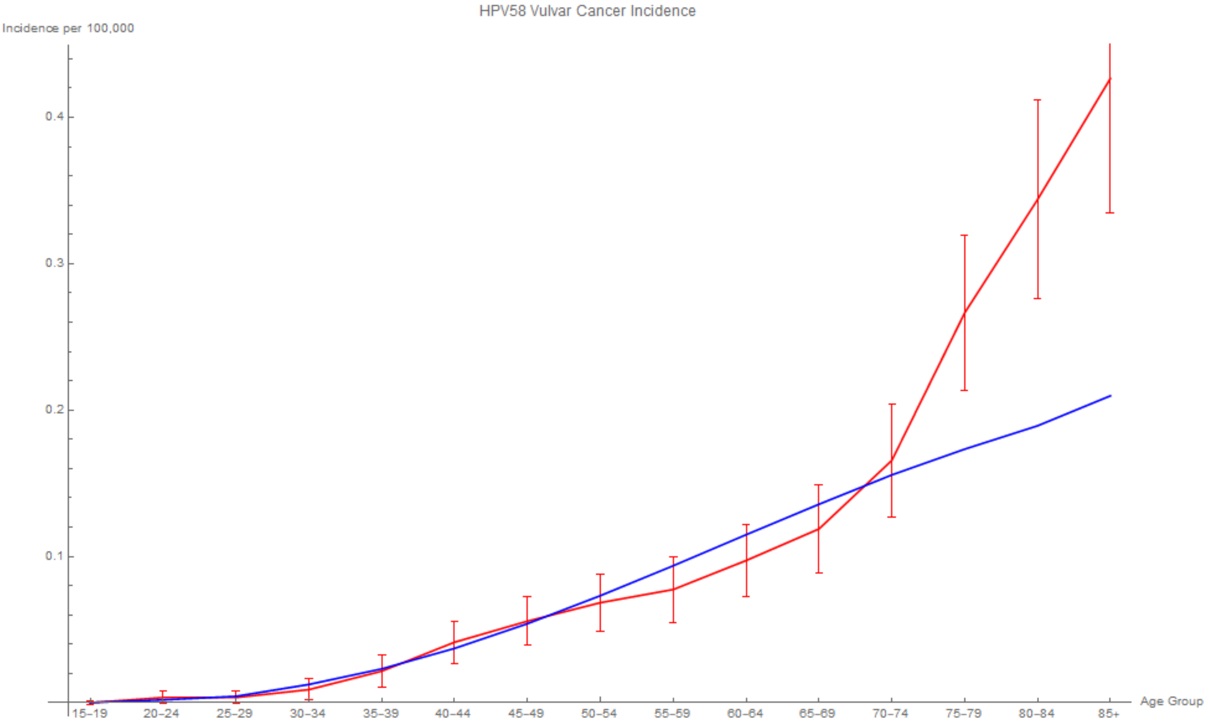

Figure 81. HPV 58 vulvar cancer incidence

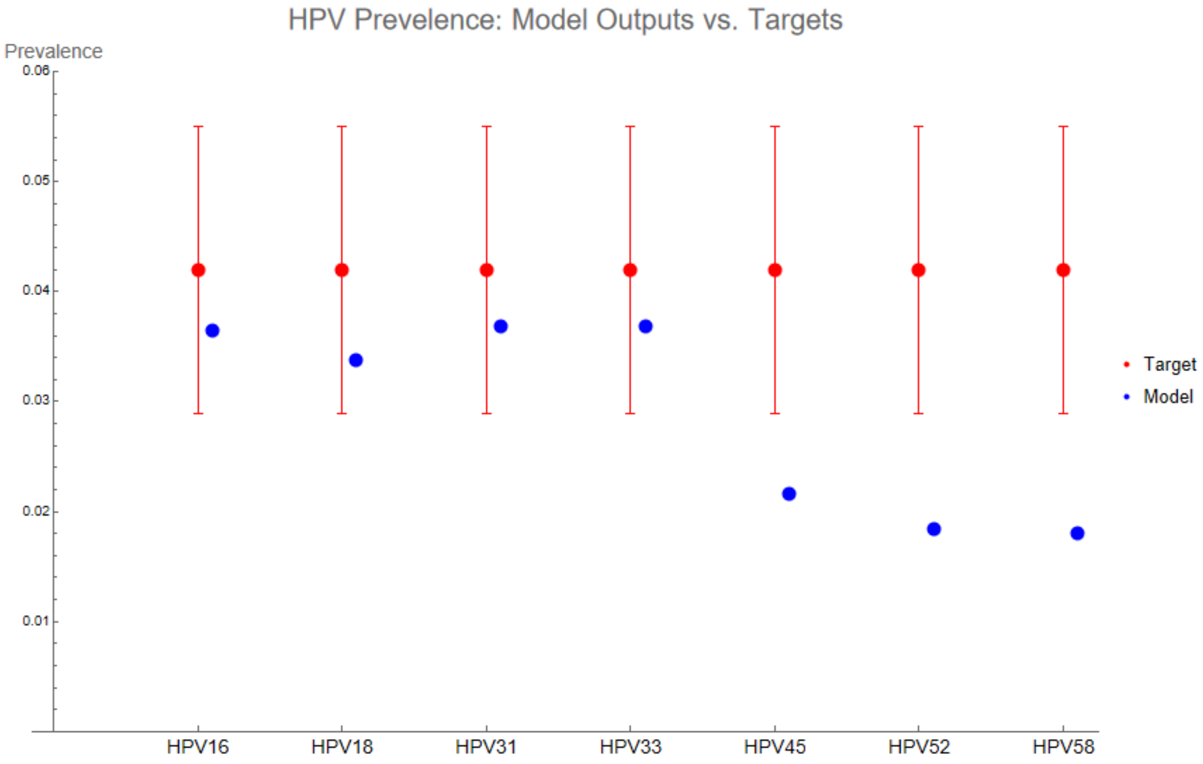

Figure 82. Vulvar infection prevalence by HPV type

12 References

1. Elbasha EH, Dasbach EJ, Insinga RP. Model for assessing human papillomavirus vaccination strategies. *Emerg Infect Dis.* 2007;13:28-41.
2. Elbasha EH, Dasbach EJ, Insinga RP. Supplementary Online Appendix: A Technical Report Accompanying Manuscript: Model for assessing human papillomavirus vaccination strategies. *Emerg Infect Dis.* 2007;13:28-41.
3. Daniels V, Prabhu VS, Palmer C, et al. Public health impact and cost-effectiveness of catch-up 9-valent HPV vaccination of individuals through age 45 years in the United States. *Hum Vaccin Immunother.* 2021:1-9.
4. Choi YH, Jit M, Gay N, Cox A, Garnett GP, Edmunds WJ. Transmission dynamic modelling of the impact of human papillomavirus vaccination in the United Kingdom. *Vaccine*. 2010;28:4091-4102.
5. Dasbach EJ, Insinga RP, Elbasha EH. The epidemiological and economic impact of a quadrivalent human papillomavirus vaccine (6/11/16/18) in the UK. *BJOG*. 2008;115:947-956.
6. Jit M, Brisson M, Laprise JF, Choi YH. Comparison of two dose and three dose human papillomavirus vaccine schedules: cost effectiveness analysis based on transmission model. *BMJ*. 2015;350:g7584.
7. Jit M, Chapman R, Hughes O, Choi YH. Comparing bivalent and quadrivalent human papillomavirus vaccines: economic evaluation based on transmission model. *BMJ*. 2011;343:d5775.
8. Jit M, Choi YH, Edmunds WJ. Economic evaluation of human papillomavirus vaccination in the United Kingdom. *BMJ*. 2008;337:a769.
9. Datta S, Pink J, Medley GF, et al. Assessing the cost-effectiveness of HPV vaccination strategies for adolescent girls and boys in the UK. *BMC Infect Dis.* 2019;19:552.
10. Joura EA, Giuliano AR, Iversen OE, et al. A 9-valent HPV vaccine against infection and intraepithelial neoplasia in women. *N Engl J Med.* 2015;372:711-723.

11. Luxembourg A, Bautista O, Moeller E, Ritter M, Chen J. Design of a large outcome trial for a multivalent human papillomavirus L1 virus-like particle vaccine. *Contemp Clin Trials.* 2015;42:18-25.

12. Merck. Clinical Report V503-P001. A randomized, international double-blinded, controlled with Gardasil®, dose-ranging, tolerability, immunogenicity, and efficacy study of a multivalent Human Papillomavirus (HPV) L1-virus like particle (VLP) vaccine administered to 16- to 26-year-old women – end of study report (at least 42 month follow-up). 2014.

13. Schubat A. HPV "Coverage." *N Engl J Med.* 2015;372:775-6.

14. Merck. Statistical Report V503-002-10. Study of the Immunogenicity, Tolerability, and Manufacturing Consistency of V503 (A Multivalent Human Papillomavirus [HPV] L1 Virus-Like Particle [VLP] Vaccine) in Preadolescents and Adolescents (9 to 15 year olds) with a Comparison to Young Women (16 to 26 year olds)-the V503-002-010-extension 2014.

15. Merck. Clinical Report V503-P003. A Phase III Clinical Trial to Study the Tolerability and Immunogenicity of V503, a Multivalent Human Papillomavirus (HPV) L1 Virus-Like Particle (VLP) Vaccine, in 16- to 26-Year-Old Men and 16- to 26-Year-Old Women 2014.

16. Kosalaraksa P, Mehlsen J, Vesikari T, et al. An open-label, randomized study of a 9-valent human papillomavirus vaccine given concomitantly with diphtheria, tetanus, pertussis and poliomyelitis vaccines to healthy adolescents 11-15 years of age. *Pediatr Infect Dis J.* 2015;34:627-34.

17. Merck. Clinical Study Report V503-P005. A Phase III Open-Label Clinical Trial to Study the Immunogenicity and Tolerability of V503 (A Multivalent Human Papillomavirus [HPV] L1 Virus-Like Particle [VLP] Vaccine) Given Concomitantly with Menactra™ and Adacel™ in Preadolescents and Adolescents (11 to 15 Year Olds) 2012.

18. Merck. Clinical Study Report V503-P007. A Phase III Open-Label Clinical Trial to Study the Immunogenicity and Tolerability of V503, a Multivalent Human Papillomavirus (HPV) L1 Virus-Like Particle (VLP) Vaccine, Given Concomitantly With REPEVAX™ in Preadolescents and Adolescents (11 to 15 Year Olds). 2012.

19. Merck. Clinical Study Report V503-P006. A Phase III Randomized, International, Placebo-Controlled, Double-Blind Clinical Trial to Study the Tolerability and Immunogenicity of V503, a Multivalent Human Papillomavirus (HPV) L1 Virus-Like Particle (VLP) Vaccine, Given to Females 12-26 Years of Age Who Have Previously Received GARDASIL™ (Protocol 006) 2012.

20. Merck. Clinical Study Report V503-P009/GDS01C. A Randomized, Double-Blinded, Controlled with GARDASIL® (Human Papillomavirus Vaccine [Types 6, 11, 16, 18] (Recombinant, adsorbed)), Phase III Clinical Trial to Study the Immunogenicity and Tolerability of V503 (9-Valent Human Papillomavirus [HPV] L1 Virus-Like Particle [VLP] Vaccine) in Preadolescent and Adolescent Girls (9- to 15-year-olds) 2012.

21. Summary of product characteristics Gardasil 9 human papillomavirus 9-valent vaccine (recombinant, adsorbed): European Medicine Agency, Committee for Medicinal Products for Human Use. March 7, 2015.

22. Rottingen JA, Garnett GP. The epidemiological and control implications of HIV transmission probabilities within partnerships. *Sex Transm Dis.* 2002;29:818-827.

23. Elbasha EH, Dasbach EJ. Impact of vaccinating boys and men against HPV in the United States. *Vaccine* 2010;28:6858-67.

24. Cervical Screening Programme, England 2013-14: Health & Social Care Information Centre; 2015 11/25/2015.

25. Population Estimates for UK, England and Wales, Scotland and Northern Ireland, Mid-2013. Office for National Statistics, 2014. Accessed January 19, 2014. http://www.ons.gov.uk/ons/publications/re-reference-tables.html?edition=tcm%3A77-322718.)

26. Elbasha EH, Dasbach EJ. Impact of vaccinating boys and men against HPV in the United States. *Vaccine*. 2010;28:6858-6867.

27. Hospital Episode Statistics, Admitted Patient Care, England—2013-14. Health & Social Care Information Centre. January 28, 2015.

28. Cancer statistics. Cancer Research UK, 2015. http://www.cancerresearchuk.org/cancer-info/cancerstats/types/.)

29. Istituto Nazionale Tumori. Eurocare Survival of cancer patient in Europe: Istituto Nazionale Tumori. 2015.

30. UK CR. Cervical cancer survival statistics. 2014.

31. Gerein V, Rastorguev E, Gerein J, Draf W, Schirren J. Incidence, age at onset, and potential reasons of malignant transformation in recurrent respiratory papillomatosis patients: 20 years experience. *Otolaryngol Head Neck Surg.* 2005;132:392-4.

32. Ault KA. Effect of prophylactic human papillomavirus L1 virus-like-particle vaccine on risk of cervical intraepithelial neoplasia grade 2, grade 3, and adenocarcinoma in situ: a combined analysis of four randomised clinical trials. *Lancet*. 2007;369:1861-1868.

33. Garland SM, Hernandez-Avila M, Wheeler CM, et al. Quadrivalent vaccine against human papillomavirus to prevent anogenital diseases. *N Engl J Med.* 2007;356:1928-1943.

34. Giuliano AR, Palefsky JM, Goldstone S, et al. Efficacy of quadrivalent HPV vaccine against HPV Infection and disease in males. *N Engl J Med.* 2011;364:401-411.

35. Joura EA, Leodolter S, Hernandez-Avila M, et al. Efficacy of a quadrivalent prophylactic human papillomavirus (types 6, 11, 16, and 18) L1 virus-like-particle vaccine against high-grade vulval and vaginal lesions: a combined analysis of three randomised clinical trials. *Lancet*. 2007;369:1693-1702.

36. Palefsky JM, Giuliano AR, Goldstone S, et al. HPV vaccine against anal HPV infection and anal intraepithelial neoplasia. *N Engl J Med.* 2011;365:1576-1585.

37. Garland SM, Hernandez-Avila M, Wheeler CM, et al. Quadrivalent vaccine against human papillomavirus to prevent anogenital diseases. *N Engl J Med*. 2007;356:1928-1943.

38. HPV Immunisation Uptake Statistics: National Services Scotland. September 30, 2014.

39. HPV vaccine uptake 1 September 2013 to 30 June 2014: Public Health England. August 30, 2014.

40. Vaccine Uptake in Children in Wales July to September 2014: Public Health Wales. December 1, 2014.

41. Martin-Hirsch P, Rash B, Martin A, Standaert B. Management of women with abnormal cervical cytology: treatment patterns and associated costs in England and Wales. *BJOG*. 2007;114:408-415.

42. Brown RE, Breugelmans JG, Theodoratou D, Benard S. Costs of detection and treatment of cervical cancer, cervical dysplasia and genital warts in the UK. *Curr Med Res Opin.* 2006;22:663-670.

43. Wade R, Spackman E, Corbett M. Adjunctive colposcopy technologies for examination of the uterine cervix—DySIS, LuViva Advanced Cervical Scan and Niris Imaging System. February 8, 2012.

44. Keeping STT, M. The Broader Burden of Human Papillomavirus: Estimates of the Cost of Treating Penile Cancer in Secondary Care in England. Public Health England Conference. 2014.

45. Keeping ST, Tempest MJ, Stephens SJ, Carroll SM, Nugent KP, O'Dwyer ST. The cost of anal cancer in England: retrospective hospital data analysis and Markov model. *BMC Public Health.* 2014;14:1123.

46. Coles V, Chapman R, Lanitis T, Carroll S. The costs of managing genital warts in the UK by devolved nation: England, Scotland, Wales and Northern Ireland. *Int J STD AIDS*. 2015.

47. Hughes OR, Tsikoudas A, Barr GD. The burden of recurrent respiratory papillomatosis in the United Kingdom: results from the BAPO and ENT-UK National Survey. *Clin Otolaryngol.*

48. Adjunctive colposcopy technologies for examination of the uterine cervix - DySIS and the Niris Imaging: costing template: National Institute for Health and Clinical Excellence; January 8, 2012.

49. Bains I, Choi YH, Soldan K, Jit M. Clinical impact and cost-effectiveness of primary cytology versus human papillomavirus testing for cervical cancer screening in England. *Int J Gynecol Cancer.* 2019;29:669-675.

50. Sullivan PW, Slejko JF, Sculpher MJ, Ghushchyan V. Catalogue of EQ-5D scores for the United Kingdom. *Med Decis Making*. 2011;31:800-804.

51. Dominiak-Felden G, Cohet C, Atrux-Tallau S, Gilet H, Tristram A, Fiander A. Impact of human papillomavirus-related genital diseases on quality of life and psychosocial wellbeing: results of an observational, health-related quality of life study in the UK. *BMC Public Health.* 2013;13:1065.

52. Alemany L, Saunier M, Alvarado-Cabrero I, et al. Human papillomavirus DNA prevalence and type distribution in anal carcinomas worldwide. I*nt J Cancer.* 2015;136:98-107.

53. de Sanjose S, Quint WGV, Alemany L, et al. Human papillomavirus genotype attribution in invasive cervical cancer: a retrospective cross-sectional worldwide study. Lancet Oncol. 2010;11:1048-1056.

54. Castellsague X, Alemany L, Quer M, et al. HPV involvement in head and neck cancers: comprehensive assessment of biomarkers in 3680 patients. *J Natl Cancer Inst.* 2016;108:djv403.

55. Alemany L, Cubilla A, Halec G, et al. Role of human papillomavirus in penile carcinomas worldwide. *Eur Urol.* 2016;69:953-961.

56. Alemany L, Saunier M, Tinoco L, et al. Large contribution of human papillomavirus in vaginal neoplastic lesions: a worldwide study in 597 samples. *Eur J Cancer.* 2014;50:2846-2854.

57. de Sanjose S, Alemany L, Ordi J, et al. Worldwide human papillomavirus genotype attribution in over 2000 cases of intraepithelial and invasive lesions of the vulva. *Eur J Cancer.* 2013;49:3450-3461.

58. Johnson AM, Mercer CH, Beddows S, et al. Epidemiology of, and behavioural risk factors for, sexually transmitted human papillomavirus infection in men and women in Britain. *Sex Transm Infect.* 2012;88:212-217.

59. Donne AJ, Keltie K, Cole H, Sims AJ, Patrick H, Powell S. Prevalence and management of recurrent respiratory papillomatosis (RRP) in the UK: cross-sectional study. *Clin Otolaryngol.* 2017;42:86-91.

60. Cody P, Tobe K, Abe M, Elbasha EH. Public health impact and cost effectiveness of routine and catch-up vaccination of girls and women with a nine-valent HPV vaccine in Japan: a model-based study. *BMC Infect Dis.* 2021;21:11.
